# Molecular and functional profiling of cell diversity and identity in the lateral superior olive, an auditory brainstem center with ascending and descending projections

**DOI:** 10.3389/fncel.2024.1354520

**Published:** 2024-05-23

**Authors:** Ayse Maraslioglu-Sperber, Erika Pizzi, Jonas O. Fisch, Kathrin Kattler, Tamara Ritter, Eckhard Friauf

**Affiliations:** ^1^Animal Physiology Group, Department of Biology, University of Kaiserslautern-Landau, Kaiserslautern, Germany; ^2^Genetics/Epigenetics Group, Department of Biological Sciences, Saarland University, Saarbrücken, Germany

**Keywords:** patch-seq, lateral superior olive (LSO), olivocochlear system, Single-cell RNA sequencing, biophysical properties, sound localization

## Abstract

The lateral superior olive (LSO), a prominent integration center in the auditory brainstem, contains a remarkably heterogeneous population of neurons. Ascending neurons, predominantly principal neurons (pLSOs), process interaural level differences for sound localization. Descending neurons (lateral olivocochlear neurons, LOCs) provide feedback into the cochlea and are thought to protect against acoustic overload. The molecular determinants of the neuronal diversity in the LSO are largely unknown. Here, we used patch-seq analysis in mice at postnatal days P10-12 to classify developing LSO neurons according to their functional and molecular profiles. Across the entire sample (*n* = 86 neurons), genes involved in ATP synthesis were particularly highly expressed, confirming the energy expenditure of auditory neurons. Two clusters were identified, pLSOs and LOCs. They were distinguished by 353 differentially expressed genes (DEGs), most of which were novel for the LSO. Electrophysiological analysis confirmed the transcriptomic clustering. We focused on genes affecting neuronal input–output properties and validated some of them by immunohistochemistry, electrophysiology, and pharmacology. These genes encode proteins such as osteopontin, Kv11.3, and Kvβ3 (pLSO-specific), calcitonin-gene-related peptide (LOC-specific), or Kv7.2 and Kv7.3 (no DEGs). We identified 12 “Super DEGs” and 12 genes showing “Cluster similarity.” Collectively, we provide fundamental and comprehensive insights into the molecular composition of individual ascending and descending neurons in the juvenile auditory brainstem and how this may relate to their specific functions, including developmental aspects.

## Introduction

Brain areas are composed of highly heterogenous cell populations with diverse structure and function. An extensive knowledge about individual cell types is required to better understand the functional organization of neural systems at different levels. Over the past two decades, next-generation RNA sequencing (RNA-seq) has paved new roads to investigate the molecular determinants of cellular diversity (Macaulay and Voet, 2014; Svensson et al., 2018). In the present study, we took advantage of recent advances in RNA-seq technologies and employed patch-seq, a powerful multi-modal single-cell approach, to decipher the biophysical and transcriptomic determinants of neuronal heterogeneity in the lateral superior olive (LSO). The LSO is a conspicuous brainstem nucleus receiving and analyzing binaural input. It contains neurons in the ascending and the descending pathway, namely principal neurons (pLSOs) and lateral olivocochlear neurons (LOCs). pLSOs are involved in sound localization, whereas LOCs enable the central auditory system (CAS) to directly control the cochlear periphery (reviews: Friauf et al., 2019; Yin et al., 2019; Owrutsky et al., 2021).

pLSOs amount to almost $\frac{3}{4}$ of all LSO neurons (Helfert and Schwartz, 1986, 1987; Franken et al., 2018), whose number slightly exceeds 1,600 in mice (Hirtz et al., 2011). By performing a subtraction-like process of excitatory and inhibitory input signals from the ipsi- and contralateral ear, respectively, pLSOs detect interaural level differences (Tollin, 2003). The main inputs are in strict tonotopic register and are glutamatergic, respectively, glycinergic with modulatory GABAergic inputs (Fischer et al., 2019). Collectively, these inputs are tuned for fast and precise synaptic transmission, even during sustained high-frequency stimulation (Krächan et al., 2017; Müller et al., 2022). pLSOs in mice have fusiform somata with a surface area of ~130 μm^2^ (Williams et al., 2022). For juvenile mice, an input resistance (R_in_) of 96 MΩ, a resting potential (V_rest_) of −64 mV, and a membrane capacitance (C_m_) of 11 pF have been reported (Sterenborg et al., 2010). Another study found 70 MΩ, −57 mV and 22 pF for mouse LSO neurons in general (Leao et al., 2006). A striking feature of pLSO neurons is a prominent inward current (I_h_) which is mediated through hyperpolarization-activated and cyclic nucleotide-modulated (HCN) channels (Leao et al., 2006; Sterenborg et al., 2010).

Two subpopulations of pLSOs can be distinguished by their intrinsic firing pattern *in vitro* (rats: Barnes-Davies et al., 2004). In mice, the first subtype, termed single spiking (Sterenborg et al., 2010) or onset-burst pLSOs (Haragopal and Winters, 2023), fires 1–5 action potentials (APs) of short latency during prolonged depolarization. The second pLSO subtype fires throughout the duration of the stimulus and has been termed multiple firing (Sterenborg et al., 2010) or multi-spiking (Haragopal and Winters, 2023).

**Figure 1:**
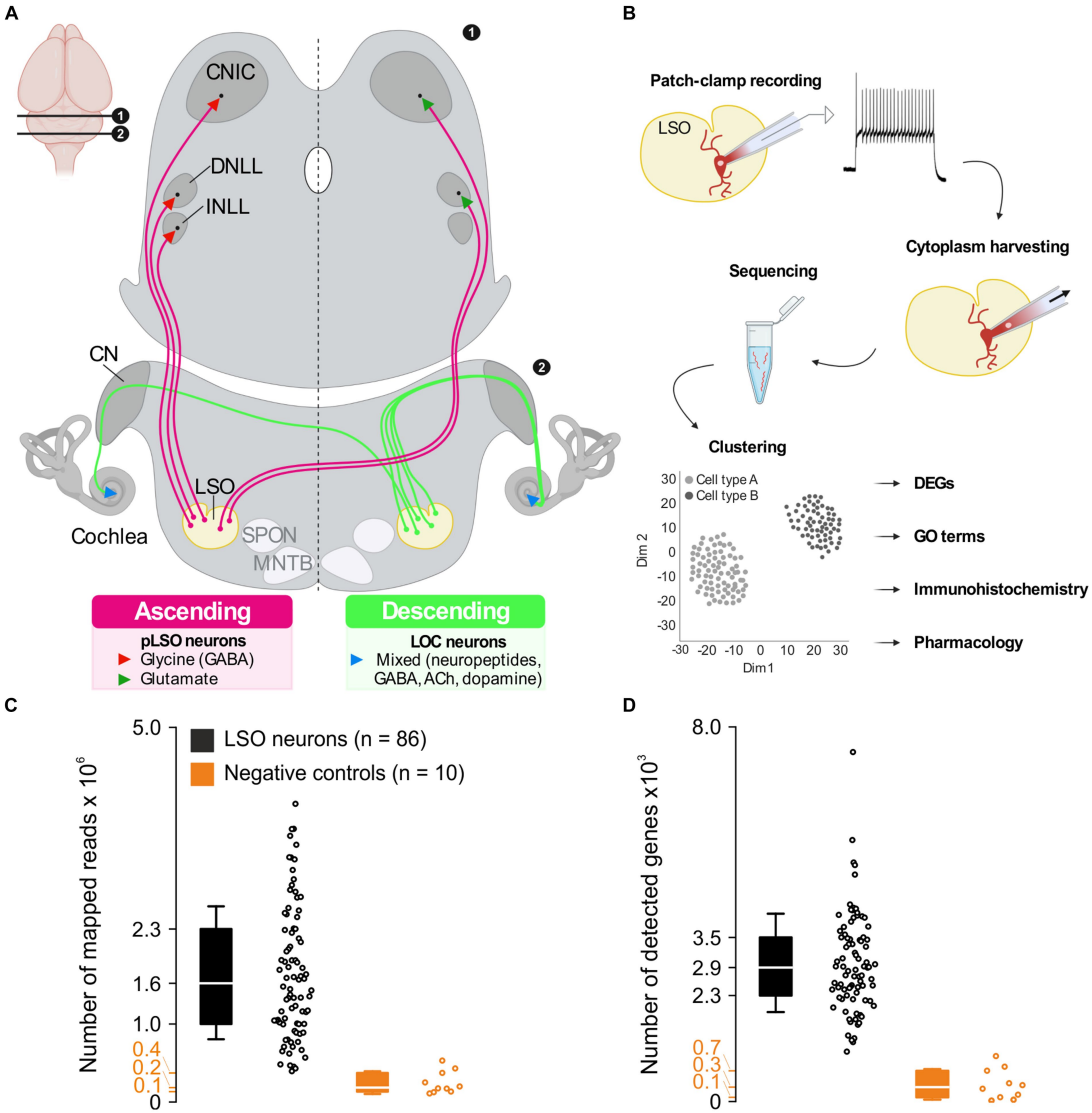
*A priori* quality checks result in 86 LSO patch-seq neurons of sufficient quality. **(A)** Ascending projections (left) and descending projections (right) of LSO neurons. Inset (top left) depicts position of coronal sections in the brain. **(B)** Patch-seq workflow. Whole-cell patch-clamp experiments were performed to assess electrophysiological properties, followed by cytoplasm harvesting. The cytoplasm was used for cDNA library synthesis and sequencing. After processing sequencing reads, unsupervised clustering was done to identify neuron types, and further analyses were performed to characterize these neuron types. **(C)** Number of mapped reads from 86 LSO neurons of sufficient quality and 10 neurons from negative controls (black and orange, respectively). **(D)** As **(C)**, but for detected genes. CN, cochlear nuclear complex; CNIC, central nucleus of the inferior colliculus; DNLL, dorsal nucleus of the lateral lemniscus; INLL, intermediate nucleus of the lateral lemniscus; LSO, lateral superior olive; MNTB, medial nucleus of the trapezoid body; SPON, superior paraolivary nucleus. See also Supplementary Figures S1, S2. In these and all subsequent box plots, center line marks median (Q2), box limits mark first and third quartile (Q1 and Q3), and whiskers represent standard deviation (SD).

pLSO neurons send ipsilateral, glycinergic projections to the intermediate nucleus and the dorsal nucleus of the lateral lemniscus (INLL, DNLL) as well as the central nucleus of the inferior colliculus (CNIC) (Figure 1A; Friauf et al., 2019; Mellott et al., 2022). pLSOs also give rise to contralateral, mainly glutamatergic projections terminating in the DNLL and CNIC (Saint Marie et al., 1989; Saint Marie and Baker, 1990; Glendenning et al., 1992; Fredrich et al., 2009; Abraham et al., 2010). As recently reported, both projection types contribute relatively equally to the pLSO output in mice (Haragopal et al., 2023).

*LOCs* are the second major neuron type in the LSO. They form one of the two branches of efferent projections of the auditory system, the second branch being composed of medial olivocochlear neurons (MOCs, reviews: Brown, 2011; Guinan, 2011). Ontogenetically, LOCs and MOCs have a motoneuronal nature (Karis et al., 2001; Simmons, 2002). The efferent auditory system comprises ~470 neurons (mice), with LOCs exceeding MOCs by ~2-fold (Campbell and Henson, 1988). In mice, LOC somata are round and have a surface area of ~100 μm^2^ (Williams et al., 2022); (Suthakar and Ryugo, 2021, report ~140 μm^2^). Most LOC somata are intrinsic to the LSO and intermingled with pLSO somata (mice); few somata reside in a shell region surrounding the LSO (Wu et al., 2020; Williams et al., 2022).

Axons of LOCs are thin and unmyelinated (Brown, 2011). Virtually all (99%) project into the ipsilateral cochlea (Guinan, 2018). The axons make *en passant* synapses primarily on radial “dendrites” of type I auditory neurons, beneath the basal pole of inner hair cells (Warr et al., 1997; Simmons, 2002). A minority of LOCs contact inner hair cells directly (Liberman, 1990; Sobkowicz and Slapnick, 1994; Sobkowicz et al., 1997). Furthermore, LOCs may project to the ventral cochlear nucleus (Ryan et al., 1990) and have axon collaterals terminating in the LSO itself and slightly outside (Brown, 1993). In line with the unmyelinated nature of their axons, peripheral effects of LOC activity are slower than MOC effects and occur over minutes. LOCs likely modulate auditory nerve fiber activity in a reflex manner (Guinan, 2011) and protect the cochlea against noise-induced hearing loss (Darrow et al., 2007; Fuchs and Lauer, 2019).

Like pLSOs, LOCs receive excitatory and inhibitory inputs, yet with longer latencies and slower kinetics (review: Friauf et al., 2019). They differ from pLSOs in several biophysical properties. For instance, they display a regular firing pattern in response to depolarizing current pulses injected *in vitro*, whereby the first AP occurs with a substantial and variable delay (25–180 ms; Adam et al., 1999). In contrast to pLSOs, LOCs lack I_h_ currents, but they exhibit prominent low-threshold and rapidly activating and inactivating transient outward K^+^ currents. By counteracting depolarizing effects of excitatory synapses, these currents are key determinants of synaptic integration and repetitive AP activity (Rothman and Manis, 2003; Manis, 2008). In accordance with their relatively small somata (Williams et al., 2022), mouse LOCs have an exceptionally low C_m_ (6 pF) (Sterenborg et al., 2010). The authors also reported a very high R_in_ (~300 MΩ) and a relatively positive V_rest_ (−43 mV). Low C_m_ values in LOCs were also obtained by Fujino et al. (1997) and Leijon and Magnusson (2014) (mouse, 15 pF; rat, 40 pF).

The neurochemistry of LOCs is complex. They are predominantly cholinergic but also contain various other transmitters (reviews: Brown, 2011; Sewell, 2011). In mice, GABAergic markers have been described in all cochlear LOC fibers (Maison et al., 2003). A smaller, non-cholinergic fraction of LOCs is dopaminergic (Eybalin et al., 1993; Gil-Loyzaga, 1995; Safieddine et al., 1997; Darrow et al., 2006). Besides these classical transmitters, several neuromodulators have been described, including calcitonin-gene-related peptide (CGRP) (Kresse et al., 1995; Maison et al., 2003; Wu et al., 2018; summaries: Robertson and Mulders, 2000; Le Prell et al., 2021), urocortin (Vetter et al., 2002; Kaiser et al., 2011), and opioid peptides such as dynorphin (Le Prell et al., 2014) or enkephalins (Eybalin, 1993; Safieddine et al., 1997). A cholinergic LOC may co-contain CGRP and opioids, and various opioids can co-localize as well (Altschuler et al., 1988; Safieddine et al., 1997; Frank et al., 2023). Therefore, LOCs belong to the growing category of multi-transmitter neurons. Taken together, LOCs, despite being only a few, dynamically fine-tune auditory nerve activity via the release of excitatory and inhibitory transmitters (Groff and Liberman, 2003). The functions of LOCs in hearing, however, are virtually unknown (Romero and Trussell, 2022). They are much less explored than MOCs, and many issues are still under investigation (Kitcher et al., 2022). For example, direct evidence that LOCs respond to sound is still missing (Fuchs and Lauer, 2019).

Previous research has primarily focused on the electrophysiological properties and differentiation of neurons in the LSO. Some studies explored the molecular determinants influencing these electrophysiological properties but used now outdated techniques such as serial analysis of gene expression (SAGE) (Koehl et al., 2004; Nothwang et al., 2006) or microarrays (Ehmann et al., 2013; Xiao et al., 2013). These methods have not been able to achieve single cell resolution, which is critical in heterogeneous nuclei such as the LSO. Recently, Frank et al. (2023) presented transcriptomic single-cell transcriptomic results for LOC neurons but not for pLSO neurons. Here, we combined electrophysiology and transcriptomics using the patch-seq technique to assess functional and molecular diversity in individual LSO neurons of juvenile mice. We functionally characterized cell types in brain slices, harvested the cytoplasm and performed genome-wide expression profiling. Our goal was to identify molecular determinants of cellular heterogeneity and similarity in the LSO. We identified two clusters, pLSOs and LOCs. Differential expression was found for 353 genes, including known and novel cell type-specific markers. In particular, Kv channel subunits were intensively analyzed. Our comprehensive study identified complex molecular fingerprints at the single-cell level, paving the way for a variety of follow-up studies.

## Methods

### Animals and ethical approval

Animal breeding and experiments were approved by the regional councils of the Land Rhineland-Palatinate according to the German Animal Protection Law (TSchG §4/3) and followed the guidelines for the welfare of laboratory animals. C57BL/6 J mice were bred in the animal facilities of the University of Kaiserslautern, and both sexes were analyzed at postnatal day (P) 11 ± 1, i.e., around hearing onset.

### Brainstem slices preparation and electrophysiology

RNase-free solutions were prepared, and equipment was cleaned as described (Cadwell et al., 2017). Coronal brainstem slices were prepared (Hirtz et al., 2011) and *in vitro* electrophysiology was performed as described (Müller et al., 2019). Briefly, slices were perfused with ASCF (in mM: 125 NaCl, 2.5 KCl, 1 MgCl_2_, 1.25 NaH_2_PO_4_, 2 sodium pyruvate, 3 *myo*-inositol, 0.44 L-ascorbic acid, 25 NaHCO_3_, 10 D-glucose, 2 CaCl_2;_ pH 7.4 when oxygenated with carbogen). Whole-cell patch-clamp recordings from LSO neurons were performed at 36.5 ± 1°C. Patch pipettes were filled with internal solution (in mM: 140 potassium gluconate, 10 HEPES, 5 EGTA, 1 MgCl_2_, 2 Na_2_ATP, 0.3 Na_2_GTP). The internal solution contained 0.8% RNase inhibitor (Invitrogen). The liquid junction potential was 15.4 mV and was corrected online.

#### Current clamp

V_rest_ values are an average over a 30-s period (sample frequency 5 kHz, otherwise 50 kHz). The initial phase of the hyperpolarizing response to a − 200-pA, 200-ms current pulse was fitted mono-exponentially (OriginPro 9.1, OriginLab) to determine the membrane time constant (τ_m_). Current pulses with increasing amplitude (−200–1,000 pA, in 50-pA steps, 200 ms) were used to approximate rheobase, determine AP latency at rheobase, firing pattern (150 pA above rheobase) and voltage sag (at −200 pA). Triangular current pulses (rise to peak 1.5 ms, decay to baseline 3.5 ms, 10 repeats) were injected to determine AP amplitude, threshold, halfwidth, and peak latency. AP halfwidth was measured at 50% of the AP peak amplitude from baseline.

#### Voltage clamp

A 200-ms negative voltage step from the holding potential (V_hold_; −70 mV) to −75 mV was applied to determine series resistance (R_s_) and R_in_ (sampled at 20 kHz). Membrane currents were recorded for 1 min at V_hold_. Peak amplitudes of spontaneous postsynaptic currents (sPSCs) and their kinetics (10–90% rise time, 100–37% decay time constant τ_decay_) were determined at V_hold_ and analyzed using Mini Analysis 6 (Synaptosoft). With a Cl-reversal potential of −112 mV, inhibitory sIPSCs were outward currents. Thus, they could be easily distinguished from excitatory sEPSCs which were inward currents. For each neuron, 100 sIPSCs and 100 sEPSCs were averaged. Data analysis was performed using custom-written IgorPro 6 and IgorPro 8 routines (WaveMetrics), and Clampfit 10 (Molecular Devices). Data visualization was performed using IgorPro, OriginPro 9.1, and CorelDRAW Graphics Suite 2019 (Corel Corporation).

### Cytoplasm harvesting

Electrophysiological characterization was limited to ~10–15 min to minimize mRNA degradation. Total RNA was then extracted into the patch pipette by gently aspirating the neuronal cytoplasm. Shrinkage of the soma and entry of the nucleus into the pipette were visually monitored under DIC optics (Supplementary Figures S1A1–A3). After successful harvesting, atmospheric pressure was restored and the pipette was slowly removed from the soma (Supplementary Figure S1B). In case of contamination of the pipette by extracellular material, absence of a membrane patch at the tip of the pipette, or soma swelling, samples were discarded (Supplementary Figures S1C1,C2,D1,D2). The contents of the pipette were collected in a reaction tube containing 2 μL lysis buffer, 0.2% Triton-X, ERCC RNA Spike-In Mix (Invitrogen, 1:400,000 dilution) and RNase inhibitor. Samples were stored at −80°C and processed further *en bloc* (library preparation and sequencing). From a total of 263 patched neurons, 103 were retained for further analysis. Ultimately, 86 neurons passed the RNA quality control (see below). One negative control was collected per experimental day. A pipette filled with internal solution was moved so that its tip touched the slice. It was then withdrawn, and the contents of the pipette were processed as for harvested cells.

### Library preparation and sequencing

Double-stranded cDNA was generated as described, with minor modifications (Picelli et al., 2013). Briefly, 1 μL of 10 mM dNTPs and 0.5 μL of Oligo-dT primer (Supplementary Table S1) were added to each sample, and samples were incubated at 72°C for 3 min and immediately placed on ice. Reverse transcription was performed using 0.5 μL SuperScript II RT (200 U/μL, Invitrogen) supplemented with 0.25 μL RNasin (40 U/μL, Promega), 2 μL Superscript II first-strand buffer (5x), 0.48 μL 100 mM DTT, 2 μL 5 M betaine, 0.12 μL 0.5 M MgCl_2_, 0.1 μL 100 μM template-switching oligonucleotide and 0.25 μL nuclease-free water. Incubation was performed at 42°C for 90 min, followed by 10 cycles at 50°C for 2 min and another incubation step at 42°C for 2 min. Enzyme inactivation was achieved by incubation at 70°C for 15 min. cDNA was pre-amplified by adding 12.5 μL KAPA HiFi HotStart ReadyMix PCR Kit (Roche), 0.25 μL IS PCR primer and 2.25 μL nuclease-free water with these thermocycling conditions: 98°C for 3 min, 18 cycles at 98°C for 20 s, 67°C for 15 s, 72°C for 6 min and final elongation at 72°C for 5 min. cDNA was purified using 0.8 X Ampure XP Beads (Beckman Coulter) and elution in 7 μL RNase free water. Quality control of randomly selected cells was assessed on an Agilent Bioanalyzer using the HS DNA Kit. On average, 450 pg. of cDNA was used as input for dual-indexed Nextera XT library preparation (Illumina). A total of 9 cycles of library amplification were performed according to the manufacturer’s recommendations. All individual single-cell libraries were pooled in equal amounts prior to purification of 100 μL pooled libraries with 1X Ampure XP Beads (Beckman Coulter) and final elution in 15 μL RNase-free water.

### Library quality control and next generation sequencing

Prior to sequencing, the library pool was quantified using the Qubit dsDNA HS Assay Kit (Invitrogen), and the fragment size distribution was checked using the High Sensitivity DNA Kit (Agilent) on an Agilent 2100 Bioanalyzer. The library pool was additionally quantified with the PerfeCTa NGS Quantification Kit (Quanta Biosciences) and normalized for clustering on a cBot (Illumina). Libraries were sequenced on a HiSeq2500 (Illumina) using dual index reads with 88 bp read length.

### Processing and analysis of single-cell RNA-seq data

Initial quality control of the raw data was performed using FastQC.^1^ Reads were trimmed using Trim Galore! (v0.4.2)^2^ to remove 3′ ends with base quality below 20 and adapter sequences. Reads were aligned to the mouse genome mm10 using STAR (Dobin et al., 2013, v2.5.2a) with a 2-pass mapping strategy per sample. PCR duplicates were detected using MarkDuplicate from the Picard tools (version 1.115).^3^ Reads aligned to mm10 were summarized to Gencode annotation vM2 (Harrow et al., 2012) using featureCounts (Liao et al., 2014, v1.5.0-p3) and counting primary alignments only (Li and Dewey, 2011, v1.3.1).

For quality control, filtering, and normalization of single-cell RNA-seq (scRNA-seq) data, we used the *SingleCellExperiment* R package (version 1.8.0, Amezquita et al., 2020) and the *scran* package (version 1.14.6; Lun et al., 2016) on Bioconductor. The deconvolution method was used to remove cell-specific library size biases, and size factors were calculated for each library using the *ComputeSumFactors* function. Of 103 aspirated cells, 17 (~16%) were discarded due to poor sequencing quality, leaving 86 neurons. Poor quality cells had >3 median absolute differences below the median for number of counts or number of transcripts. Genes with an average of at least one count in at least 5% of the neurons qualified for downstream analysis, resulting in 11,659 expressed genes. Transcripts per million (TPM) values were calculated by normalizing for gene length and sequencing depth (Zheng et al., 2020). The top 500 highly expressed genes were selected for Kyoto Encyclopedia of Genes and Genomes (KEGG) enrichment analysis using gene set enrichment analysis (GSEA) performed with DAVID 2021 (Huang et al., 2009; Sherman et al., 2022).

Clustering was performed using SC3 (Kiselev et al., 2017) and Seurat (Satija et al., 2015). Principal component analysis (PCA) was performed using the Seurat function *RunPCA* (Lun et al., 2016). Normalized log_2_-transformed highly variable gene (HVG) counts were used as input. t-Distributed Stochastic Neighbor Embedding (tSNE) (Kobak and Berens, 2019) plots were generated with *RunTSNE*.

DEGs were detected using the *findMarkers* function from the *scran* package, which uses a *t*-test by default. A gene was defined as a DEG if the normalized count showed a ≥ 2-fold upregulation (log_2_FC ≥ 1) and a false discovery rate (FDR) ≤ 0.05 (-log_10_FDR ≤ 1.3). DEGs were ranked by their Area Under the Receiver-Operating Characteristic curve (AUROC). DEGs were also selected for Gene Ontology (GO) enrichment analysis using GSEA performed with DAVID 2021. GO terms were condensed using REVIGO (Jiang and Conrath algorithm) (Supek et al., 2011). UpSet plots were generated using the UpSetR package (v.1.4.0) (Conway et al., 2017).

#### Super DEGs and cluster similarity

DEG analysis is a widely used method for assessing differences between samples. However, at least one pitfall is associated with very low expression levels. A gene may qualify as a DEG even if the average expression level in the two samples is very low (e.g., *Scn4b*|Navβ4, DEG#173 in pLSO, showed 1.5 vs. 0.0 TPM; Supplementary Figure S8A and Supplementary Table S6). To account for this caveat, we applied a more stringent approach and condensed the DEGs that showed an expression level of ≥10 TPM in ≥1 cluster. If the normalized counts differed ≥4-fold (log_2_FC ≥ 2), these DEGs were defined as “Super DEGs.” In addition, we used the same “≥10 TPM in ≥1 cluster” criterion to determine “Cluster similarity,” defined as ≤2-fold difference between clusters (log_2_FC ≤ 1). We further assessed Cluster similarity by using the overlapping index (OI), a means to quantify the similarity of datasets in terms of their distributions (*cf.* chapter” Super DEGs and Cluster similarity between pLSOs and LOCs”; OI = 0 → no overlap, OI = 1 → perfect overlap; Pastore and Calcagni, 2019).

To make the data sets more explorable, we have added the file in Supplementary Table S6. It lists the complex gene expression profiles for each neuron and allows the user to interactively change the selection criteria, such as the TPM threshold, and to assess how the changes affect the percentage of cells expressing a gene.

### Data availability statement

The single-cell RNA sequencing data generated during this research project has been submitted to NCBI GEO and can be accessed using the accession number GSE241761.

### Immunohistochemistry

Lethally anesthetized mice (7% chloral hydrate, 0.01 mL/g body weight or 10% ketamine, 220 μg/g body weight plus 2% xylazine, 24 μg/g body weight) were transcardially perfused with 100 mM phosphate-buffered saline (PBS, pH 7.4) at room temperature (RT), followed by ice-cold 4% paraformaldehyde (PFA) for 20 min. Brains were removed from the skull, postfixed for 2 h in 4% PFA (4°C) and stored in 30% sucrose-PBS at 4°C overnight. For Kv7.2, Kv7.3, and osteopontin, brain fixation was performed with Zamboni solution. 30-μm-thick brainstem slices were cut with a sliding microtome (HM 400 R, MICROM) and transferred to 15% sucrose-PBS, followed by three rinses in PBS (30 min, RT). Incubation and rinsing steps were performed free-floating unless otherwise noted. For Kvβ3, a heat-induced epitope retrieval protocol was used prior to antibody treatment. For this, slices were immersed in 10 mM sodium citrate buffer (pH 6.0) plus 0.05% Tween 20 (Roth) and heated at 95°C for 20–40 min. They were then returned to RT, followed by three rinses in PBS (5 min, RT).

#### Fluorescence labeling

Sections were incubated in blocking solution (0.3% Triton X-100, 10% goat serum [donkey serum for osteopontin], 3% BSA in KCl-free PBS) for 2 h at RT. Antibodies to osteopontin (1:250, goat, R&D Systems), vGLUT1 (1:500, guinea pig, Synaptic Systems), GlyT2 (1:250, mouse, Synaptic Systems), CGRP (1:500, guinea pig, Synaptic Systems), Kv7.2 (1:500, rabbit, Synaptic Systems), Kv7.3 (1:500, rabbit, Synaptic Systems), Kv11.3 (1:250, rabbit, Alomone) and Kvβ3 (1:200, rabbit, Biomol) were applied overnight at 4°C in carrier solution (0.3% Triton-X-100, 1% goat serum [donkey serum for osteopontin], 1% BSA in KCl-free PBS) solution followed by three rinses in PBS (30 min, RT). The sections were then incubated for 90 min in the dark with secondary antibodies (1,500, goat anti-guinea pig AlexaFluor 488, goat anti-rabbit AlexaFluor 568, Thermo Fisher Scientific; goat anti-rabbit AlexaFluor 488, Invitrogen; goat anti-mouse AlexaFluor 568, donkey anti-goat AlexaFluor 488, Molecular Probes; donkey anti-goat AlexaFluor 568, Invitrogen). After three PBS rinses (30 min, RT), sections were mounted on gelatin-coated slides and coverslipped with mounting medium containing 2.5% 1,4-diazabicyclo [2.2.2] octane (Sigma-Aldrich). Images were captured with a TCS SP5 X confocal microscope equipped with an HCX PL APO Lambda blue 63x oil objective (Leica Microsystems).

#### 3,3′-diaminobenzidine labeling

To block endogenous peroxidase, sections were incubated for 30 min in 10% methanol, 3% H_2_O_2_ KCl-free PBS, rinsed in PBS (three times 10 min, RT), and transferred to blocking solution for 2 h at RT. Primary antibodies against CGRP or osteopontin were applied overnight at 4°C, followed by three rinses in PBS (30 min, RT). The sections were then incubated with biotinylated secondary antibodies (1:100, goat anti-guinea pig, Rockland; 1:100, donkey anti-goat, Jackson) in carrier solution for 90 min at RT and rinsed in PBS (three times 10 min, RT). NeutrAvidin-HRP (1,500, Invitrogen) was applied in carrier solution overnight at 4°C. After three rinses in PBS (30 min, RT), the sections were incubated in DAB solution (0.7 mg/mL, SigmaFast DAB tablets) for 5 min, followed by H_2_O_2_ solution (0.7 mg/mL, SigmaFast urea hydrogen peroxide tablets) for 17 min and rinsed in PBS (three times 10 min, RT). Finally, the sections were mounted on slides and images were captures using a light microscope equipped with Plan-Neofluar objectives (Axioscope 2; 2.5x/0.12; 20x/0.5; 40x/0.75;100x/1.3 oil objective, Carl Zeiss) or an F-View 2 camera (Olympus Soft Imaging Solutions).

#### Calculation of the number of fluorescent cells

The number of immunolabeled cell bodies was determined using the ImageJ Cell Counter plugin. Three LSO sections per animal (*N* = 4) were divided into a medial and a lateral half, in which three arbitrarily placed squares (edge length 100 μm) defined the 30,000-μm^2^ region of interest from which somata were counted. A total of 24 regions of interest were analyzed.

### Pharmacology

The Kv7.2/3 agonist retigabine (N-(2-amino-4-(4-fluorobenzylamino)phenyl) carbamic acid ethyl ester; Sigma-Aldrich) was dissolved in water and stored at 4°C. On the day of the experiment, this stock solution (100 mM) was diluted, and retigabine (30 μM) was applied 10 min before and throughout the electrophysiological measurements. To analyze drug effects, we used the same current step protocol as described above in the electrophysiology section, but sampled at 20 kHz.

### Statistics

Statistical analyses were performed using Excel (Microsoft) or GraphPad Prism 9 (GraphPad Software). Data were tested for normal distribution using the Kolmogorov–Smirnov test. If normally distributed, they were compared in a 2-tailed Student’s *t*-test. Otherwise, a Mann–Whitney-U test (unpaired data) or a Wilcoxon signed-rank test (paired data) was used. The Šidák correction was used for multiple comparisons.

### Allen mouse brain atlas for *in situ* hybridization images

For selected genes, we compared the expression level to *in situ* hybridization results from the AMBA.^4^ We primarily analyzed P56 sections. They were chosen because >40% of the genes of interest were missing data at P14. P14 images in the AMBA were mainly sagittal views and less informative. Therefore, they were analyzed for comparison purposes only. The images show slightly modified coronal or sagittal sections (Supplementary Figures S4, S5). They represent gene expression energy values per 200-μm voxel. To allow direct navigation to an image of interest, we have provided the hyperlinks in Supplementary Table S7.

## Results

### *A priori* checks revealed 86 patch-seq LSO neurons of sufficient quality

The LSO is a brainstem nucleus containing neuron types with ascending and descending projections (Figure 1A). To gain a comprehensive understanding about the molecular and physiological complexity of this prominent nucleus, we performed patch-seq experiments in acute slices (Figure 1B). We recorded from 263 patch-clamped LSO neurons and harvested the cytoplasm in 103 cases (see Methods for exclusion criteria). Of these 103, 86 passed the quality controls and displayed 1.6 × 10^6^ mapped reads (median; interquartile range (IQR) 1.3 × 10^6^; Figure 1C). Negative controls yielded an 8-fold lower number (median: 0.2 × 10^6^; IQR: 0.2 × 10^6^). The mean mapping rate exceeded 80% and was thus >2-fold higher than in negative controls (Supplementary Figure S2A). The largest proportion of reads mapped to exons (45%), an almost 3-fold higher value than in negative controls (Supplementary Figures S2C,D). The median percentage of ribosomal RNA was 3.8% (IQR: 4.5%; Supplementary Figure S2B). With one exception, no single value exceeded 15%, indicating sufficient rRNA depletion (Dobin and Gingeras, 2015). The number of detected genes per neuron or negative control correlated with the number of reads mapped. A mean of 2,863 genes were found per neuron (median: 2.9 × 10^3^; IQR: 1.2 × 10^3^; Figure 1D) and 310 genes in negative controls, a > 9-fold lower value (median; IQR: 565; Figure 1D). The number of mapped reads, number of detected genes, mapping rate, and rRNA rate were consistent with published patch-seq datasets (Cadwell et al., 2017, 2020), demonstrating a high library quality.

### Highest gene expression levels imply high energy demands in LSO neurons

**Figure 2:**
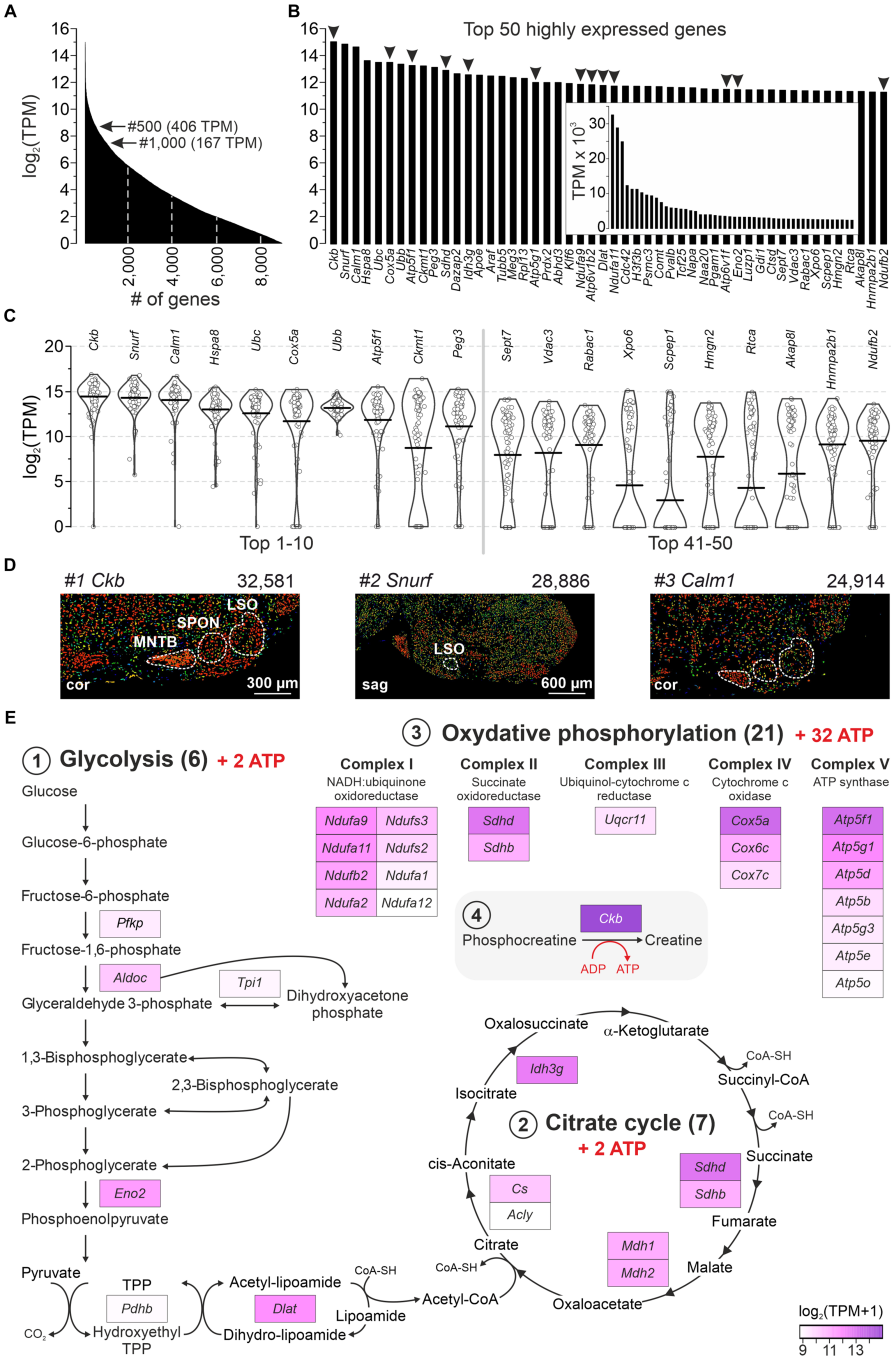
A majority of highly expressed genes reveal high energy demands for LSO neurons. **(A)** Expression levels of 9,082 genes with mean TPM ≥ 1 (log_2_). Arrows point to genes #500 and #1,000. **(B)** Top 50 highly expressed genes ranked by expression level. Arrowheads mark 13 genes involved in ATP synthesis (*cf.* **E**). **(C)** Violin plots for transcripts #1–10 and #41–50 from **(B)** (1 dot per neuron). Horizontal bars correspond to mean values. **(D)** *In situ* hybridization images of the SOC for the top three highly expressed genes (*Ckb, Snurf, Calm1;* modified images from AMBA; for URLs, see Supplementary Table S7). Expression mask image displays highlight cells that have the highest probability of gene expression (from low/blue to high/red). TPM values are also provided. **(E)** GSEA of the 500 most highly expressed genes (≥ 406 TPM). Three KEGG pathways: (1) Glycolysis (M00001, M00307), (2) Citrate cycle (M00009), (3) OxPhos (mmu00190). (4) ATP synthesis-related *Ckb* (#1). cor, coronal; sag, sagittal (see also Supplementary Table S2).

A major advantage of the single-cell patch-seq approach, which is absent from bulk analyses (Koehl et al., 2004; Nothwang et al., 2006; Ehmann et al., 2013; Xiao et al., 2013; Moritz et al., 2015; Kolson et al., 2016), is the ability to focus on neurons instead of globally addressing different cell types (including glia or blood endothelium). We took advantage of this and examined the most abundant transcripts in the 86 LSO neurons. Within the cohort of 9,082 genes with ≥1 TPM, 185 and 60 showed TPM values >1,024 and > 2,048, respectively (Figure 2A). The 50 most highly expressed genes are shown in Figure 2B (32,581–2,460 TPM, inset). For 20 of them, bee swarm and violin plots are depicted in Figure 2C. Three genes showed exceptionally high expression levels, namely *Ckb*, *Snurf*, and *Calm1* (Figure 2B; mean TPM: 32,581, 28,886, 24,914). They encode brain-type creatine kinase B (CKB), SNRPN upstream open reading frame protein (SNURF; SNRPN = small nuclear ribonucleoprotein polypeptide N), and calmodulin 1 (CALM1 aka CaM), respectively. Our findings are corroborated by *in situ* hybridization results from the AMBA which show high signal intensities in the major nuclei of the superior olivary complex (SOC) (Figure 2D). Further information on *Ckb*, *Snurf*, and *Calm1* is provided in the Discussion.

Glucose consumption is a signature of brain activity (Ashrafi and Ryan, 2017) and is particularly high in the CAS (Sokoloff, 1981). As 13 of the 50 most highly expressed genes in our sample encode enzymes involved in catabolism, we examined pathways of energy demand and ATP production in more detail. We analyzed gene enrichment in pathways that completely combust glucose to CO_2_ and H_2_O: (1) Glycolysis (including Pyruvate oxidation), (2) Citrate cycle, and (3) Oxidative phosphorylation (OxPhos; Supplementary Table S2). The analysis included 162 genes, including *Ckb* (4). Among the top 500 highly expressed genes, 7% (33) belonged into these pathways and were distributed across the four categories (Figure 2E). When normalized to the set of 162, the frequency was 20%. Taken together, these numbers emphasize and specify the genes involved in ATP generation and highlight the importance of energy metabolism in the LSO.

Gene enrichment analysis revealed seven genes among the top 500 that encode v-ATPase subunits (*Atp6ap1*, *Atp6v1b2*, *Atp6v6v11*, *Atp6v0c*, *Atp6v0d1*, *Atpv1e1*, *Atp6v1d*; 3,589–438 TPM; #24 to #460). Twenty-three v-ATPase genes have been described (Alexander et al., 2011). v-ATPases are associated with lysosomes and primary-active proton pumps which play an important role in driving transmitter loading into synaptic vesicles (Egashira et al., 2015). Our results indicate that LSO neurons were in a metabolically active state.

### scRNA-seq reveals two clusters of LSO neurons

**Figure 3:**
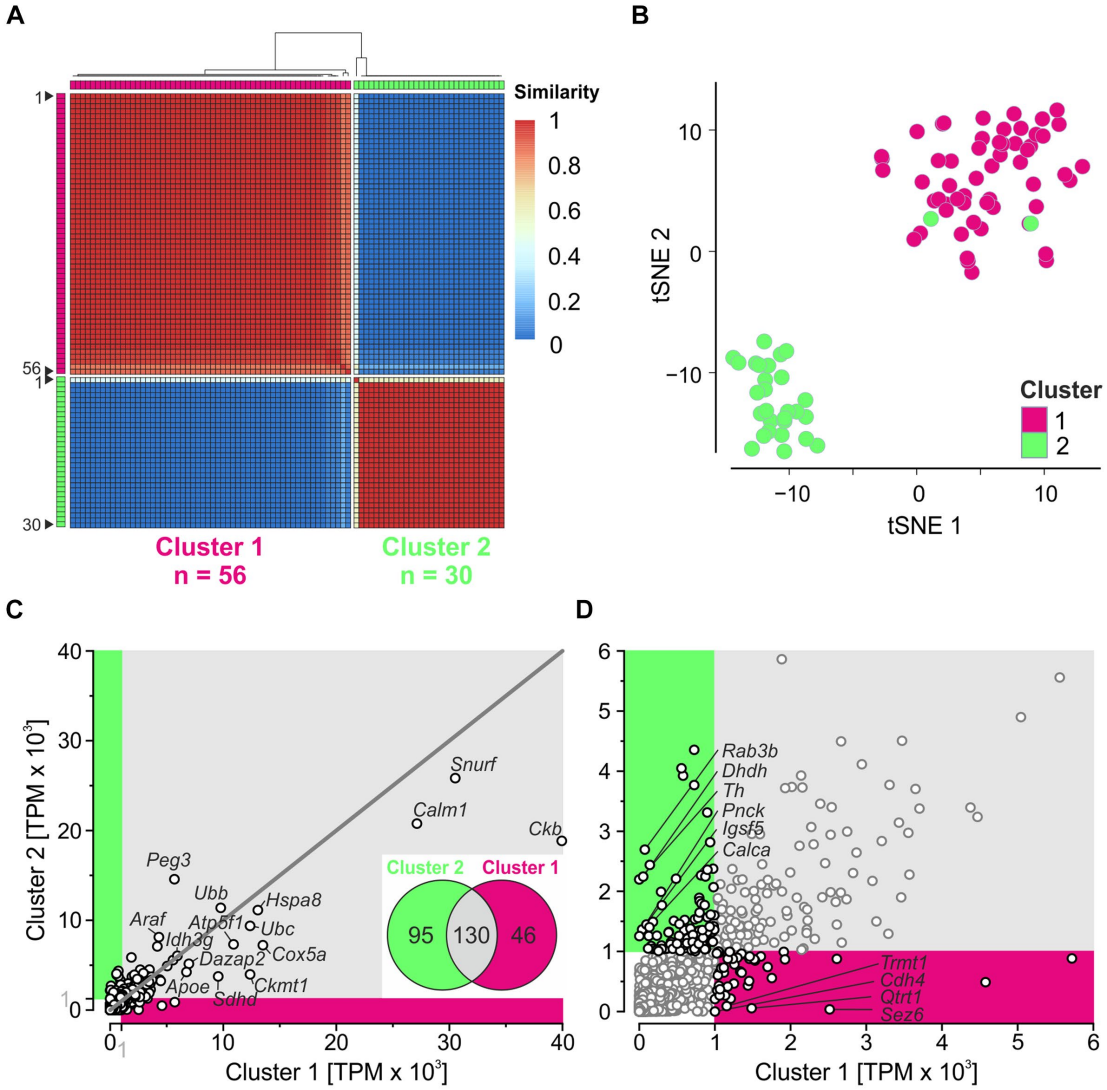
Unsupervised clustering reveals two major clusters. **(A)** Consensus matrix obtained for two clusters. Matrix shows how often each pair of neurons (*n* = 86) is assigned to the same cluster (1 = 100%). **(B)** tSNE plot based on PCs 1–10. **(C)** Correlation analysis of global expression profiles for 11,659 genes. Magenta and green sections include non-intersecting genes with mean TPM ≥ 1,000 in cluster 1 and cluster 2, respectively. The top 15 highly expressed genes (*cf.* Figure 2B) are provided with names. Dots on the 45-degree line imply same gene expression levels in both clusters. Inset: Venn diagram showing the number of intersecting (Cluster 1 ∩ Cluster 2; *n* = 130) and non-intersecting genes (Cluster 1\Cluster 2) ∪ (Cluster 2\Cluster 1; *n* = 46 + 95 = 141). **(D)** Close-up of non-intersecting genes with ≥1,000 TPM (black dots). For genes with <100 TPM in the complementary cluster, names are provided (*n* = 4, 6) (see also Supplementary Figure S3).

To identify neuron types in the LSO, we applied unsupervised clustering. We determined the consensus matrix for 2–10 clusters using SC3 and calculated the average silhouette width for each number of clusters (widths vary from 0 to 1; a width close to 1 represents the optimal number of clusters). We obtained the largest width (0.98) with two clusters (Supplementary Figures S3A,B). Fifty-six of the 86 neurons (~2/3) belonged to cluster 1, and 30 (~1/3) belonged to cluster 2 (Figure 3A). Similarity values were close to 1 for each neuron in its own cluster and close to 0 in the other cluster, except for neuron #1 in cluster 2. We checked for consistent results using the alternative tool Seurat, which assigned 59 neurons to cluster 1 and 27 neurons to cluster 2 (Supplementary Figure S3C). Fifty-six neurons in cluster 1 overlapped between SC3 and Seurat, while 27 neurons overlapped in cluster 2. The substantial overlap demonstrated consistency between the two tools. For three clusters, the results were less consistent and the overlap between SC3 and Seurat was small (Supplementary Figure S3D). Taken together, these results confirm the best results with two clusters.

To visualize the clustering results, we performed a PCA based on HVG (Methods and Supplementary Figure S3E). PC1 explained 11% of the variance and separated both clusters, except for two neurons belonging to cluster 2. Because PC1 plus PC2 together explained only 17% of the variance, we generated a tSNE plot using PC1–PC10. Again, the neurons were clearly separated into two clusters, except for two neurons (Figure 3B; see also Supplementary Figure S3E). Overall, these results demonstrate two major clusters for our sample, regardless of the clustering method used. Below, we explore the two clusters obtained by SC3.

After dividing the 86 patch-seq neurons into two clusters, we assessed the expression level of 11,659 genes (Figure 3C). Two hundred and seventy-one genes exceeded TPM ≥ 1,000 in at least one cluster. Approximately 50% of the genes overlapped (130/271; inset), indicating considerable similarity between the two clusters. Each of the 13 genes involved in ATP synthesis shown in Figure 2B ended up in the intersection, suggesting high metabolic demands in both clusters. Among the 141 non-intersecting genes with ≥1,000 TPM (46 in cluster 1; 95 in cluster 2), only four and six, respectively, showed <100 TPM in the complementary cluster (Figure 3D). This again indicates homogeneity between the two clusters for many transcripts.

### Search for molecular signatures in each cluster

**Figure 4:**
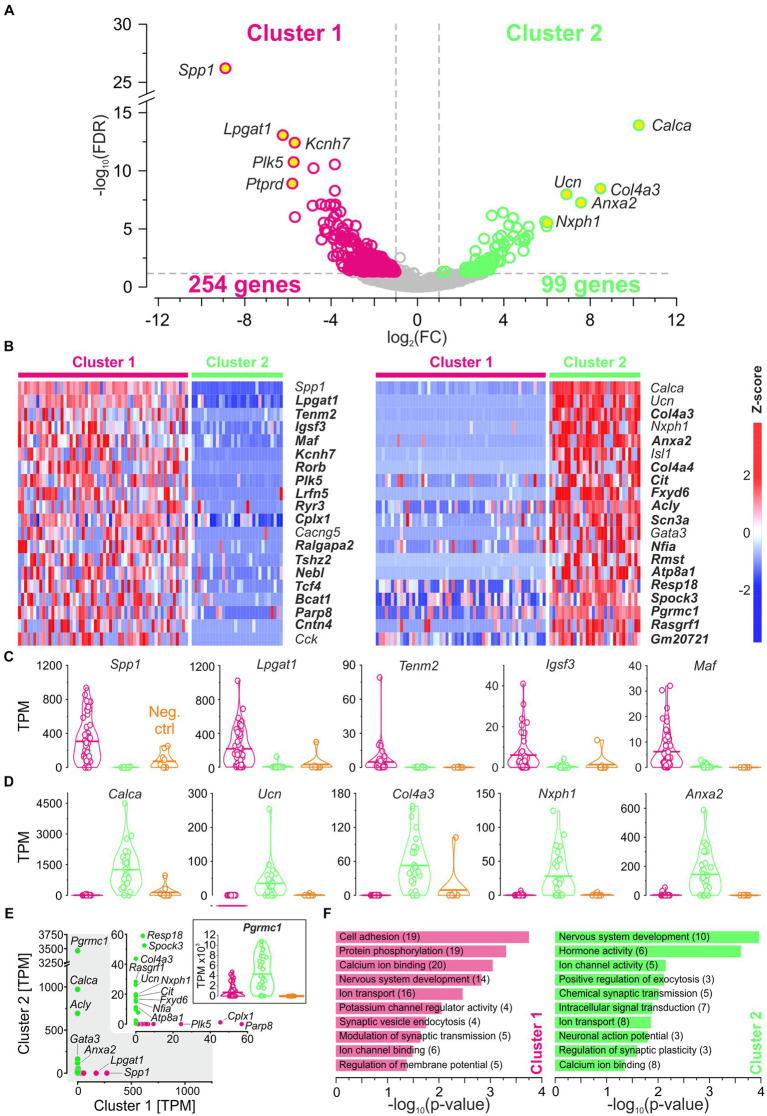
DEGs affiliate cluster 1 with pLSOs and cluster 2 with LOCs. **(A)** Volcano plot depicting DEGs for each cluster. DEGs#1–5, as ranked by FC values, are provided with names (yellow dots). Dashed gray lines depict cut-off levels (FC = 2; FDR = 0.05). **(B)** Heat maps of z-scores for the top 20 DEGs in each cluster. Thirty-two newly detected genes are marked in bold. **(C)** Violin plots for DEGs#1–5 in cluster 1 (magenta). Horizontal bars depict mean values. Values from cluster 2 (green) and negative controls (orange) are shown for comparison. **(D)** As **(C)**, but for cluster 2. **(E)** Scatter plot comparing the median expression levels of DEGs#1–20 from cluster 1 and 2. Inset is a close-up for ≤60 TPM. Genes with >10 TPM are provided with names (5 in cluster 1, 15 in cluster 2). Violin plot for *Pgrmc1*, DEG#18 in cluster 2. **(F)**, Selected enriched GO terms for DEGs in cluster 1 (magenta) and in cluster 2 (green). Number of genes in brackets (see also Supplementary Tables S3, S4).

The above comparisons revealed considerable similarities between the clusters. To identify differences, we focused on DEGs (see Methods for definition). As documented in a volcano plot, 353 DEGs were identified, of which 254 were upregulated in cluster 1 and 99 in cluster 2 (Figure 4A; Supplementary Table S3). To identify the top DEGs, the DEGs were ranked by the AUROC value. In Figure 4B, the expression of the top 40 DEGs, 20 in each cluster, is visualized in heatmaps (56 neurons in cluster 1, 30 in cluster 2). Below and in Supplementary results, we provide some background information for each of these top 40 DEGs.

### Cluster 1, DEGs#1–6

#### *#1—Spp1:* osteopontin

**Figure 5:**
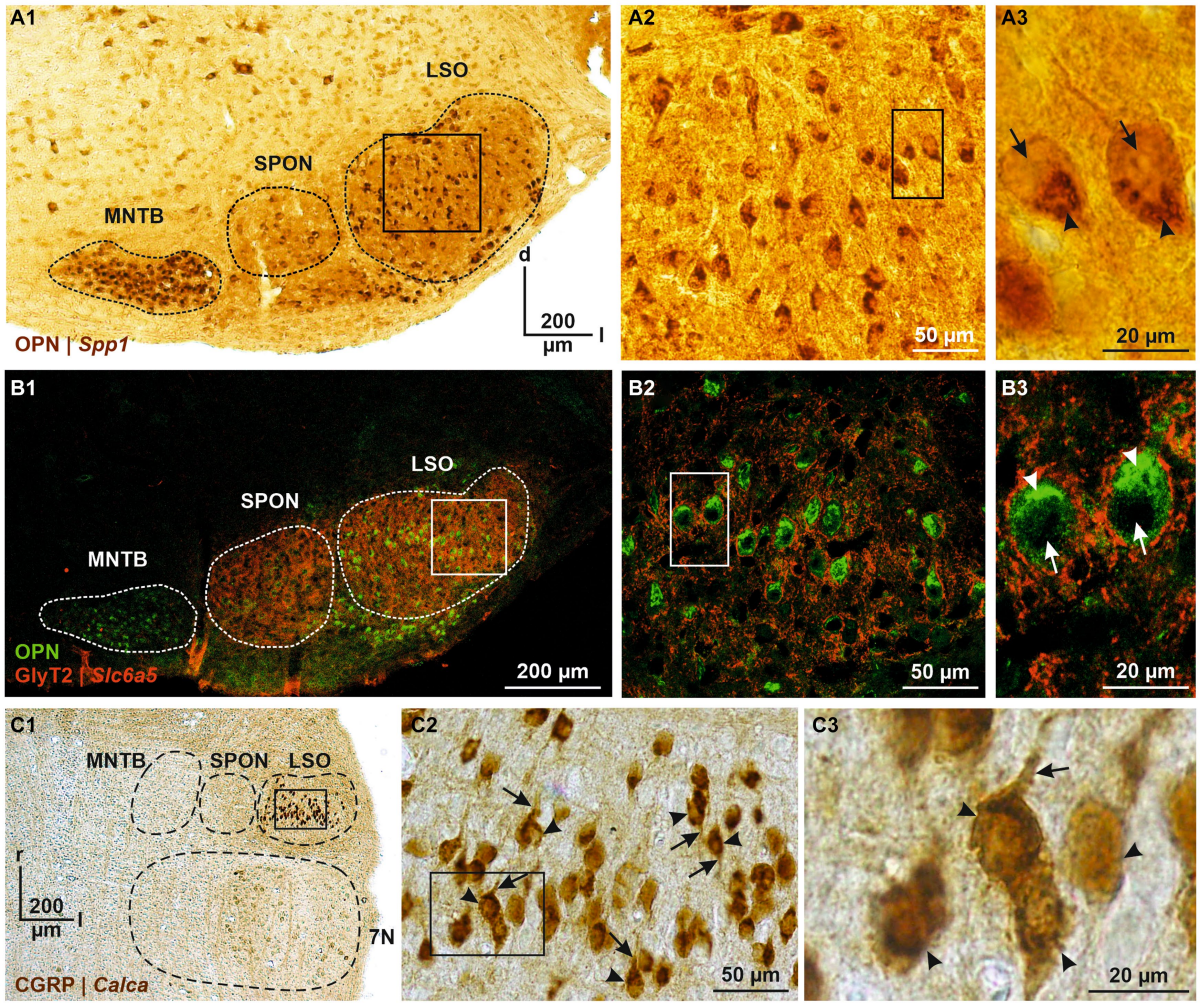
Immunohistochemical analysis of OPN and CGRP, encoded by DEG#1 in cluster 1 and cluster 2 (*Spp1* and *Calca*, respectively). **(A1)** OPN immunoreactivity in the SOC (**A1**, coronal brainstem section) and LSO **(A2)**. DAB-stained coronal brainstem slice at P12. Close-up of two representative neurons in **(A3)**. **(B)** As **(A)**, but double labeling for OPN (green) and GlyT2 (red). In **(A3,B3)**, cytoplasmic immunosignals are marked by arrowheads and nuclei by arrows. **(C)** CGRP immunoreactivity in the SOC (**C1**, horizontal brainstem section) and LSO **(C2)**. DAB-stained horizontal brainstem slice at P12. Close-ups of four representative neurons in **(C3)**. In **(C2,C3)**, somatic and dendritic immunosignals are marked by arrowheads and arrows, respectively. Borders of MNTB, SPON, 7 N, and LSO are indicated by dashed black or white lines. d, dorsal; l, lateral; r, rostral; 7 N, facial nucleus.

DEG#1 in cluster 1 was *Spp1* (Figures 4B,C,E). *Spp1* codes for the secreted phosphoprotein 1 = osteopontin (OPN), a cell adhesion molecule with multiple functions (Denhardt and Guo, 1993). Its expression is reported to be high in >20 brainstem regions, and OPN mRNA has been described in some LSO neurons of P14 rats (see Figure 3C in Lee et al., 2001). Our results from P11 mice are consistent with this pattern (see Figures 5A,B). In the rat cochlear nuclear complex, *Spp1* expression resulted in 5-14-fold higher mRNA levels in the two ventral nuclei than in the dorsal nucleus (see Figure 5 in Friedland et al., 2006). *Spp1* contributes to neuronal development (Jiang et al., 2019), including axon myelination (Selvaraju et al., 2004; Nam et al., 2019; Cappellano et al., 2021) and axon regeneration after injury (Duan et al., 2015). It has been suggested that OPN is involved in the formation and/or maintenance of axons with high conduction velocity (Higo et al., 2010). Notably, pLSO and MNTB neurons are myelinated (as are neurons in the ventral cochlear nuclei and motoneurons), whereas LOCs are not. The AMBA shows high to intermediate expression levels for *Spp1* in the LSO at P14 and at P56 (Supplementary Figure S4). Corresponding immunohistochemical views of the OPN protein pattern are shown in Figures 5A,B. For *Spp1* and subsequent genes, the expression level and numerical details are provided in Supplementary Table S3. In summary, our *Spp1*|OPN results support the idea that pLSOs belong to cluster 1, whereas LOCs do not.

#### *#2—Lpgat1*: lysosphatidylglyceral acyltransferase 1

DEG#2 in cluster 1 was *Lpgat1* (Figures 4B,C,E). This gene codes for lysophosphatidylglycerol acyltransferase 1 (LPGAT1), an enzyme associated with the endoplasmic reticulum and involved in lipid metabolism (Yang et al., 2004). LPGAT1 regulates triacylglycerol synthesis which is critical to maintain the integrity of mitochondrial membranes (Wei et al., 2020). In the vestibular organ of chickens, the protein has been exclusively identified in hair cells (Herget et al., 2013). The AMBA shows high *Lpgat1* expression in the LSO at P56 (Supplementary Figure S4, no AMBA data available for P14).

**Figure 6:**
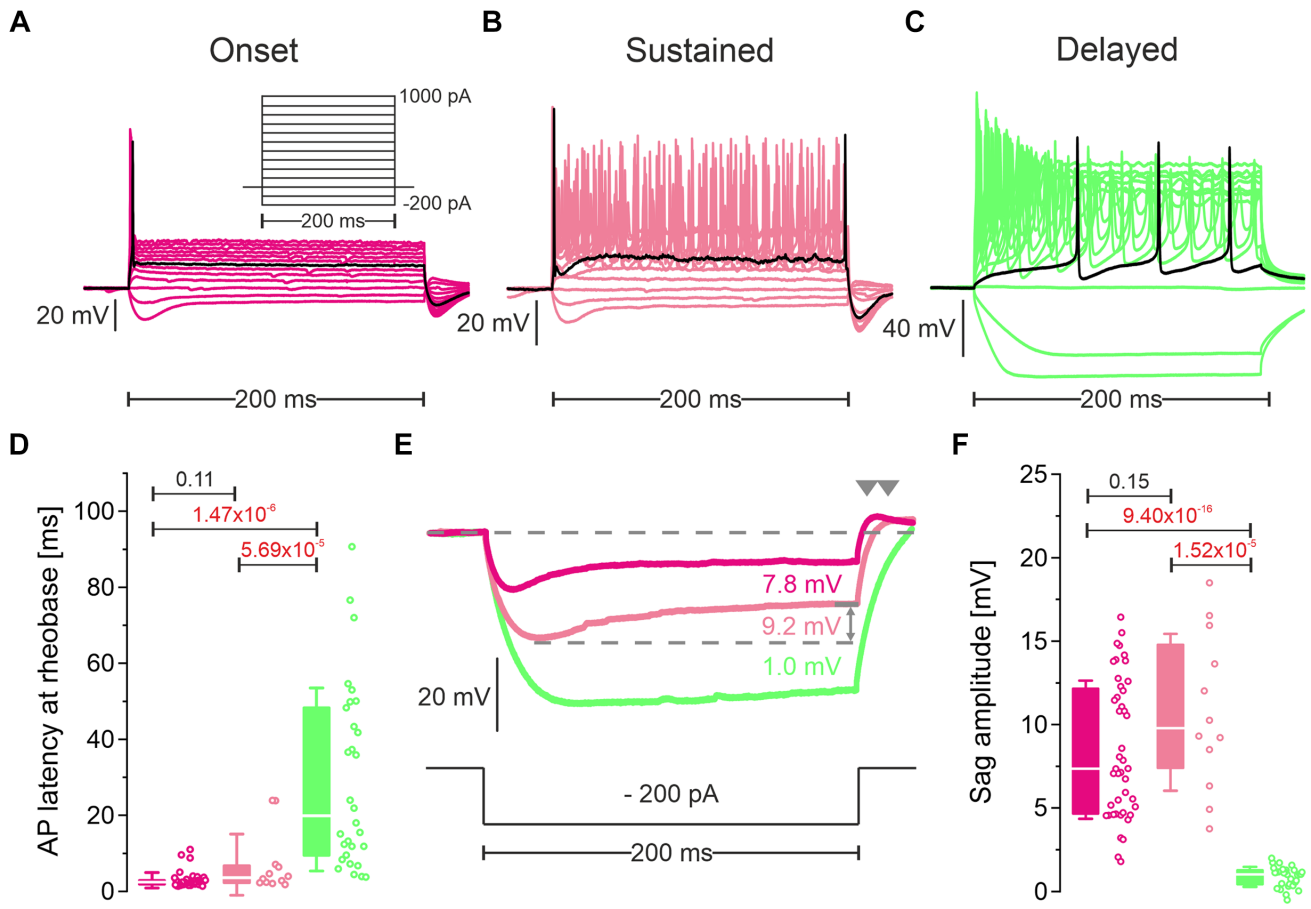
Cluster 1 neurons and cluster 2 neurons differ in their firing pattern and their response to hyperpolarization. **(A–C)** Representative voltage traces in response to rectangular current pulses (inset in **A**; −200 to +1,000 pA, 100 pA increments, 200 ms duration) of an onset firing cluster 1 neuron (pLSO—Onset; **(A)**, a sustained firing cluster 1 neuron (pLSO—Sustained; **(B)**, and a delayed firing cluster 2 neuron (LOC—Delayed; **(C)**. Black traces depict responses at rheobase. Notice different voltage scaling. **(D)** Statistics for the peak latency of the first AP at rheobase. Open dots depict single neurons. Numbers above bars represent *p*-values (in red if significant). **(E)** Voltage traces in response to a − 200-pA|200-ms current pulse highlighting sag behavior. Same color code as in **(A–C)**. Sag amplitudes were quantified as indicated by the gray double arrow. Rebound behavior after the hyperpolarizing current pulse is marked by gray arrowheads. **(F)** As **(D)**, but for sag amplitude (see also Supplementary Table S5).

#### #3—*Tenm2:* teneurin transmembrane protein 2

DEG#3 in cluster 1 was *Tenm2* (Figures 4B,C). *Tenm2* belongs to the teneurin family that comprises four members (*Tenm1-4*). It codes for teneurin transmembrane protein 2 (TEN2, aka TENM2), an axon guidance and adhesion molecule that interacts across the synaptic cleft with presynaptic latrophilin1, thus mediating Ca^2+^ signaling and synapse formation (Vysokov et al., 2016). Teneurin proteins are abundant throughout the central nervous system and play a role in regulating synaptic partner matching (Hong et al., 2012; Leamey and Sawatari, 2014). A distinct role of *Tenm2* in the generation of binocular projections has been demonstrated in the mouse visual system, and *Tenm2* knockout resulted in specific wiring deficits in the retinogeniculate pathway (Young et al., 2013). In the auditory system, high *Tenm2* expression is present in spiral ganglion neurons of the Ib subtype, whereas type II neurons highly express *Tenm3* (Petitpré et al., 2018). As to date, information about *Tenm2* in the CAS is missing. The AMBA shows a few cells with low *Tenm2* expression in the LSO at P56 (Supplementary Figure S4, no AMBA data available for P14).

#### #4*—Igsf3:* immunoglobulin superfamily member 3

DEG#4 in cluster 1 was *Igsf3* (Figures 4B,C). It belongs to the Ig superfamily whose members are major regulators of many developmental processes, such as differentiation, axogenesis, dendritogenesis, and synaptogenesis. Like the other three members of the little studied EWI Ig subfamily, IGSF3 (aka EWI-3) contains a Glu-Trp-Ile (EWI) motif. IGSF3 is a neuron-specific membrane protein that is produced in various neuronal populations when neural circuits are formed (Usardi et al., 2017). It is transiently found in cerebellar granule cells before their final maturation and highly concentrated in axon terminals. The AMBA shows only two cells expressing *Igsf3* in the LSO at P56 (Supplementary Figure S4, no AMBA data available for P14).

#### #5—*MAF:* MAF bZIP transcription factor

DEG#5 in cluster 1 was *Maf* (Figures 4B,C). The *Maf* family encodes b leucine zipper (bZIP)-containing transcription factors which act as homo-or hetero-dimers. Depending on the binding site and binding partner, the encoded proteins are transcriptional activators or repressors. cMAF controls eye and lens development (Ring et al., 2000) and directs the development of low-threshold touch receptors (Wende et al., 2012). Pacinian corpuscles, which are essential to detect small-amplitude high-frequency vibrations, become severely atrophied in *c-Maf* mutants. Several genes encoding K^+^ channels are downregulated in such mutants (*Kcna1, Kcng4, Kcnh5, Kcnq4*). In the cerebral cortex, c-MAF regulates the potential of GABAergic interneurons to acquire a somatostatin-positive identity, shortly after these neurons become postmitotic (Mi et al., 2018). The AMBA shows high expression levels for *Maf* in the LSO at P14 and at P28 (Supplementary Figure S4).

#### #6—*Kcnh7:* potassium voltage-gated channel subfamily H member 7

DEG#6 in cluster 1 was *Kcnh7* (Figure 4B). Kv11.3, the encoded protein, is the third member in the family of ERG channels (ether-a-go-go-related). Our study demonstrates no gene expression for Kv11.2 and only low expression for Kv11.1 (*cf*. chapter “Potassium channels”). Consistent with our results, the AMBA shows low to intermediate *Kcnh7* expression in the LSO at P14 and P56 and few cells with high signal intensity at P56 (Supplementary Figure S4). Below, we further analyze *Kcnh7*|Kv11.3 on the protein level (*cf*. chapter “Immunohistochemical analysis of Kv11.3 and Kvβ3”), and we refer to the Discussion for an elaborate treatise.

#### #7–20

Information on DEGs#7–20 is provided in Supplementary results.

### Cluster 2, DEGs#1–6

#### *#1—Calca*: calcitonin gene-related peptide

DEG#1 in cluster 2 was *Calca* (Figures 4B,D,E). *Calca* codes for CGRP, the calcitonin gene-related peptide. As elaborated in the Introduction, CGRP is present in LOCs but absent from pLSOs. Therefore, our DEG results provide initial hints that cluster 2 may comprise LOCs. To assess whether the protein follows mRNA, we performed immunohistochemical analysis of CGRP and demonstrated intense labeling in the LSO (Figure 5C), therewith confirming previous results (Safieddine and Eybalin, 1992; Robertson and Mulders, 2000). AMBA shows LSO cells with high gene expression at P14 and intermediate to high expression at P56 (Supplementary Figure S5).

#### *#2—Ucn*: urocortin

The tentative conclusion that cluster 2 comprises LOCs was reinforced by our findings on DEG#2, which was *Ucn* (Figures 4B,D,E). *Ucn* codes for urocortin, one of three members in the urocortin family composed of stress-related corticotropin-releasing hormone neuropeptides. In rats, urocortin was demonstrated in a small subset of LSO neurons (Vaughan et al., 1995; Kaiser et al., 2011). It is reportedly absent from other auditory nuclei (Bittencourt et al., 1999). *Ucn* expression appears to be specific for LOCs, and urocortin is abundant in their axon terminals in the cochlea (Vetter et al., 2002; Kaiser et al., 2011). Consistent with our results, AMBA shows LSO cells with high gene expression levels at P14. Interestingly, *Ucn* expression P56 is almost absent (Supplementary Figure S5). As LOCs modulate the excitability of auditory nerve fibers, urocortin may balance physiological reactions to acoustic stress via high affinity binding to corticotropin-releasing factor receptor CRFR1 during development (Graham and Vetter, 2011).

#### *#3—Col4a3*: collagen type IV alpha 3 chain

DEG#3 in cluster 2 was *Col4a3* (Figures 4B,D,E). *Col4a3* codes for the α3 domain of type IV collagen. Type IV collagens are the major structural component of basement membranes, including the strial capillary basement membranes in the inner ear. *Col4a3* mutations affect the structure and function of the cochlea and result in syndromic deafness (Mochizuki et al., 1994; Angeli et al., 2012; Kolla et al., 2020). Thus, *Col4a3* is considered a deafness gene (Müller and Barr-Gillespie, 2015). As to date, the literature is virtually devoid of reports on *Col4a3* in combination with neurons. Type IV collagens inhibit cell proliferation and astroglial differentiation while promoting neuronal differentiation (Ali et al., 1998). In this context, it is of some interest that DEG#7 in cluster 2 was *Col4a4*, which encodes the α4 domain of type IV collagen (Figure 4B). AMBA shows low *Col4a3* expression in a few cells in the LSO at P56 (Supplementary Figure S5, no AMBA data available for P14). However, *Col4a3* was identified very recently as a marker for LOCs in a study that employed single-nucleus sequencing in mice (Frank et al., 2023). Our results confirm these findings.

#### *#4—Nxph1*: neurexophilin 1

DEG#4 in cluster 2 was *Nxph1* (Figures 4B,D,E). *Nxph1* codes for the neuronal glycoprotein neurexophilin1. Four isoforms of neurexophilin (Nxph1-Nnxph4) form a conserved family of neuropeptide-like molecules that interact with neurexins, crucial presynaptic cell-adhesion molecules (Missler and Südhof, 1998; Ding et al., 2020). Nxph1 is a specific ligand for α-neurexin 1 (aka NRXN1α) and essential for transsynaptic activation. Neurexins (NRXN1-3) consist of an α-and a β-protein and play important roles in synaptic plasticity and synapse maturation. By binding to the postsynaptic ligand, they can directly influence ligand-binding receptors, thereby altering a neuron’s excitatory or inhibitory ability. A restricted *Nxph1* expression was described in subpopulations of inhibitory neurons (Petrenko et al., 1996; summary: Born et al., 2014). Conjunctively, these transsynaptic cell adhesion molecules coordinate synapse formation, restructuring, and elimination in both directions. From in the Petrenko paper, one can deduce that LSO neurons express neurexophilin. AMBA shows a low to intermediate expression level in the LSO at P56 (Supplementary Figure S5, no AMBA data available for P14).

#### *#5—Anxa2*: annexin A2

*Anxa2*, DEG#5 in cluster 2 (Figures 4B,D,E), is one of 13 annexin A genes expressed in vertebrates. Members of this subfamily are soluble proteins that are recruited to membranes in the presence of elevated Ca^2+^ where they bind phospholipids. Among the multiple roles of annexins are membrane aggregation, exocytosis, and endocytosis regulation. *Anxa2* is highly expressed in nociceptors where AnxA2 regulates TRPA1-dependent acute and inflammatory pain (Avenali et al., 2014). Gene expression was also demonstrated in surrounding cells in the developing mouse cochlea (Scheffer et al., 2015). To our knowledge, relevant papers on auditory neurons do not exist (Pubmed search: “Anxa2 auditory” or “Annexin A2 auditory”; 2023–10-22). AMBA shows only a few LSO cells with low *Anxa2* expression at P14 and P56 (Supplementary Figure S5).

#### *#6—Isl1:* insulin gene enhancer protein ISL1

*Isl1* (aka *Islet-1*), DEG#6 of cluster 2 (Figure 4B), is a homeobox gene encoding the insulin gene enhancer protein ISL1. The gene product, a transcription factor, promotes motoneuron development (Ericson et al., 1992) and affects efferents to the inner ear. *Isl1* expression has been reported in olivocochlear efferents (Frank and Goodrich, 2018). Our patch-seq results are consistent with these reports. As the inner ear efferents originate in a progenitor zone for motoneurons, it is not surprising that factors assigning a motoneuron identity (e.g., ISL1 and ChAT) overlap considerably between such efferents and facial motoneurons. In fact, inner ear efferents represent a subset of motoneurons, although they form no synapses with muscle fibers (Frank and Goodrich, 2018). AMBA shows few LSO cells with intermediate to high *Isl1* expression at P56 (Supplementary Figure S5, no AMBA data available for P14).

#### #7–20

Information on DEGs#7–20 is provided in Supplementary results.

### Summary for DEGs#1–20 of each cluster

Taken together, DEGs#1–20 in cluster 1 plus cluster2 contain eight genes (20%) whose expression was previously described in the LSO (cluster 1: *Spp1*, *Cacng5*, *Cck*; cluster 2: *Calca*, *Ucn*, *Nxph1*, *Isl1*, *Gata3*). We now attribute their expression to neurons. The results for the remaining 80% of the genes are new. Each of the three known DEGs in cluster 1 can be associated with pLSOs. Similarly, four genes from cluster 2 can be associated with LOCs (*Calca*, *Ucn*, *Isl1*, *Gata3*). Thus, the transcriptomic results provide substantial and consistent evidence to conclude that cluster 1 and cluster 2 contain pLSOs and LOCs, respectively. If our conclusion is correct (see below), we identified 17 novel marker genes for pLSOs and 15 for LOCs (highlighted in bold in Figure 4B).

Figure 4B shows 40 DEGs ranked by the z-score. Corresponding expression levels are detailed in violin plots (Figures 4C,D), highlighting significant expression differences. We also plotted the corresponding TPM values for both clusters (Figure 4E and inset). The highest expression in cluster 2 was seen for *Pgrmc1* (3,462 TPM). However, this DEG did not end up higher than #18 (Figure 4B), because 50% of cluster 1 neurons also expressed it, at levels up to 4,763 TPM (framed inset in Figure 4E). In cluster 1, TPM values were relatively low, and the highest value was found for *Spp1* (273; 0 in cluster 2). Overall, 20 of the 40 DEGs showed TPM values ≤10 (15 in cluster 1; 5 in cluster 2). For these, we did not provide names (Figure 4E and inset, gene names provided only if >10 TPM).

We compared our data with AMBA’s P14 and P56 data, covering 17 of the top 40 DEGs at P14 (42.5%) and 38 DEGs at P28/P56 (95%). A developmental assessment revealed virtually no change in the expression profile of six DEGs in pLSO neurons, while two transcription factors (*Rorb*, *Tcf4*) and *Cacng5* appeared to be downregulated with age. Among the marker genes for LOC neurons, seven genes (*Calca, Ucn, Cit, Fxyd6, Gata3, Nfia, Atp8a1*) showed lower levels at P56, with only two showing no change, indicating developmental downregulation.

### Search for functional groups among DEGs

Next, we analyzed the 353 DEGs according to GO annotations. Several GO terms were enriched (Figure 4F; Supplementary Table S4). More specifically, the terms “Nervous system development,” “Calcium ion binding,” and “Ion transport” appeared in both clusters. Within the GO term “Hormone activity,” which was unique for cluster 2, *Calca* and *Ucn* (DEG#1 and #2) encode CGRP and urocortin, established neurotransmitters for LOCs (*cf.* Introduction). The third gene in this GO term was *Uts2b* (DEG#25), which encodes the neuromodulator urotensin 2B (Supplementary Table S3). Urotensin 2B is involved in motoneuron function and stimulates ACh release (Huitron-Resendiz et al., 2005; Nothacker and Clark, 2005). The fourth “Hormone activity” gene was *Calcb* (DEG#34). It is a paralog of *Calca* and codes for the calcitonin related polypeptide β-CGRP (formerly known as CGRP-II; Alexander et al., 2021). β-CGRP enables calcitonin receptor binding activity and participates in regulating the cytosolic Ca^2+^ concentration. Recently, *Calcb* expression has been associated with a subset of LOCs (Frank et al., 2023). The fifth gene in the GO term was *Igf1* (DEG#58), which encodes the insulin growth factor 1 (IGF-I). IGF-I is involved in differentiation and maturation of neurons and synapses (Nieto-Estévez et al., 2016). Finally, the sixth “Hormone activity” gene was *Vgf* (DEG#77), which encodes the neurosecretory protein VGF (= VGF nerve growth factor inducible). VGF is a neuropeptide precursor involved in neurogenesis and metabolic regulation.

Collectively, the results from the unsupervised clustering analysis clearly affiliate the two clusters with pLSOs and LOCs.

### Immunohistochemical detection of proteins complies with gene expression (*Spp1*|OPN and *Calca*|CGRP)

We identified *Spp1*|OPN as DEG#1 in cluster 1 (presumably pLSOs) and *Calca*|CGRP as DEG#1 in cluster 2 (presumably LOCs). To assess correspondence between gene expression and protein abundance, we performed immunohistochemical experiments. Immunosignals for OPN were intense in the LSO and other SOC nuclei (Figures 5A1,B1). They were spread throughout the LSO and were associated with somata and proximal dendrites of cells reminiscent of pLSOs, because of their fusiform shape and size (Figures 5A2,A3,B2,B3). Labeling was particularly strong in a cup-shaped structure within the cytoplasm and virtually absent from the cells’ nucleus. Double labeling with glycine transporter type 2 (GlyT2), used to mark the perisomatic zone, demonstrated cytoplasmic OPN localization, rather than an association with the plasma membrane (Figures 5A3,B3). Conjunctively, the results demonstrate a correspondence between high *Spp1* expression and a high OPN abundance, implying a close correlation between mRNA and protein level. Moreover, OPN appears to be associated with pLSOs.

Because of the elaborate demonstration of CGRP in coronal sections (references in Introduction), we characterized the protein in horizontal sections (Figure 5C). CGRP immunosignals were strong in the LSO, as for OPN. In contrast, they were virtually absent from other SOC nuclei (Figure 5C1). Labeled somata were located along the tonotopic axis (from lateral to medial), and their size was a bit smaller than for OPN-positive somata (Figures 5C2,C3). Immunopositive dendrites were oriented perpendicular to the tonotopic axis, as described for LOCs (Friauf et al., 2019). Cytoplasmic labeling was more homogeneous than for OPN. Together, the *Calca*|CGRP findings imply a close relationship between gene expression and protein abundance. They also provide additional evidence that cluster 2 comprises LOCs. In the following, we will exploit the electrophysiological properties of our patch-seq neurons to assess whether cluster 1 comprises pLSOs, whereas cluster 2 comprises LOCs.

### Electrophysiology: pLSOs form cluster 1, LOCs form cluster 2

A great advantage of the patch-seq method is its potential to characterize electrophysiological parameters prior to scRNA-seq. We determined 16 physiological features prior to cytoplasm harvesting. Because of the comprehensive knowledge about such features and the differences between pLSOs and LOCs (Sterenborg et al., 2010), we assessed whether the two clusters are also distinguishable by electrophysiological parameters.

One important biophysical feature is the firing pattern. Among cluster 1 neurons, we observed two types of firing pattern. At rheobase, ~80% (44/56) neurons displayed a single AP with a short latency (Figures 6A,D; Supplementary Figure S6B; Supplementary Table S5.1). We refer to them as “pLSO—Onset.” The remaining 20% of cluster 1 neurons fired ~2 APs near rheobase (Figure 6B; Supplementary Figure S6B). The latency of the first AP was longer than for Onset neurons (7.0 ms vs. 2.9 ms; Supplementary Table S5.1). We refer to these neurons as “pLSO—Sustained.” Cluster 2 neurons displayed a delay-type repetitive firing pattern that clearly distinguished them from cluster 1 neurons (Figure 6C, Supplementary Figure S6B; Supplementary Table 5.1). Near rheobase, ~4 APs were generated, and the latency of the first AP (29.4 ms) was several-fold longer than for Onset and Sustained neurons. These neurons are named “LOC—Delayed.” A delay-type repetitive firing pattern is characteristic for LOCs, identified as such via retrograde labeling from the cochlea (Fujino et al., 1997). The authors correlated the firing pattern to A-currents.

The approximated rheobase values differed statistically between the three neuron types (Supplementary Figure S6A). With increasing current pulse amplitudes, pLSO-Onset neurons kept firing only few APs (Supplementary Figure S6B). In contrast, Sustained neurons increased their firing rate linearly, whereas Delayed neurons peaked and declined thereafter. These findings are in accordance with previous studies on pLSOs and LOCs in gerbils (Sanes, 1990; Walcher et al., 2011), rats (Kandler and Friauf, 1995; Fujino et al., 1997; Adam et al., 1999, 2001), and mice (Sterenborg et al., 2010; Walcher et al., 2011; Haragopal and Winters, 2023). Therefore, they provide independent physiological evidence that cluster 1 consists of pLSOs whereas cluster 2 is composed of LOCs.

pLSOs and LOCs can be distinguished by a sag behavior of the membrane potential in response to hyperpolarizing current steps. We found prominent sag behavior in Onset and Sustained neurons, but a virtual absence in Delayed neurons (Figures 6E,F). These findings confirm published results. pLSOs specifically show a depolarizing rebound behavior after the termination of hyperpolarizing current pulses (Kandler and Friauf, 1995; Walcher et al., 2011). Indeed, we found a rebound behavior in Onset and Sustained neurons, but not in the Delayed type (Figure 6E).

We further compared V_rest_, R_in_, τ_m_, C_m_ as well as the AP peak amplitude, halfwidth, and threshold across the three neuron types. Of these seven biophysical features, R_in_ and τ_m_ were significantly higher in Sustained neurons than in Onset neurons, whereas six parameters differed between Delayed neurons and each pLSO subtype (rheobase, V_rest_, R_in_, τ_m_, AP halfwidth, AP threshold; Supplementary Figures S6C–G,I,J). The AP amplitude did not differ significantly between the three groups (Supplementary Figures S6G,H). In a last step, we assessed six synaptic parameters, namely the peak amplitude, the rise time, and the τ_decay_ of sEPSCs and sIPSCs. Whereas none of the six parameters was statistically distinguishable between Onset and Sustained neurons, Delayed neurons differed significantly in 9/12 cases when compared to the two subtypes of pLSOs (Supplementary Figure S7).

Overall, the electrophysiological results, together with our findings on DEGs, provide sufficient evidence to conclude that pLSOs belong to cluster 1, whereas LOCs belong to cluster 2. Moreover, the differences between the two subtypes in cluster 1 are too small to warrant a subcategorization into two subclusters.

### Transcriptional pLSO and LOC clusters comply with electrophysiological differences

**Figure 7:**
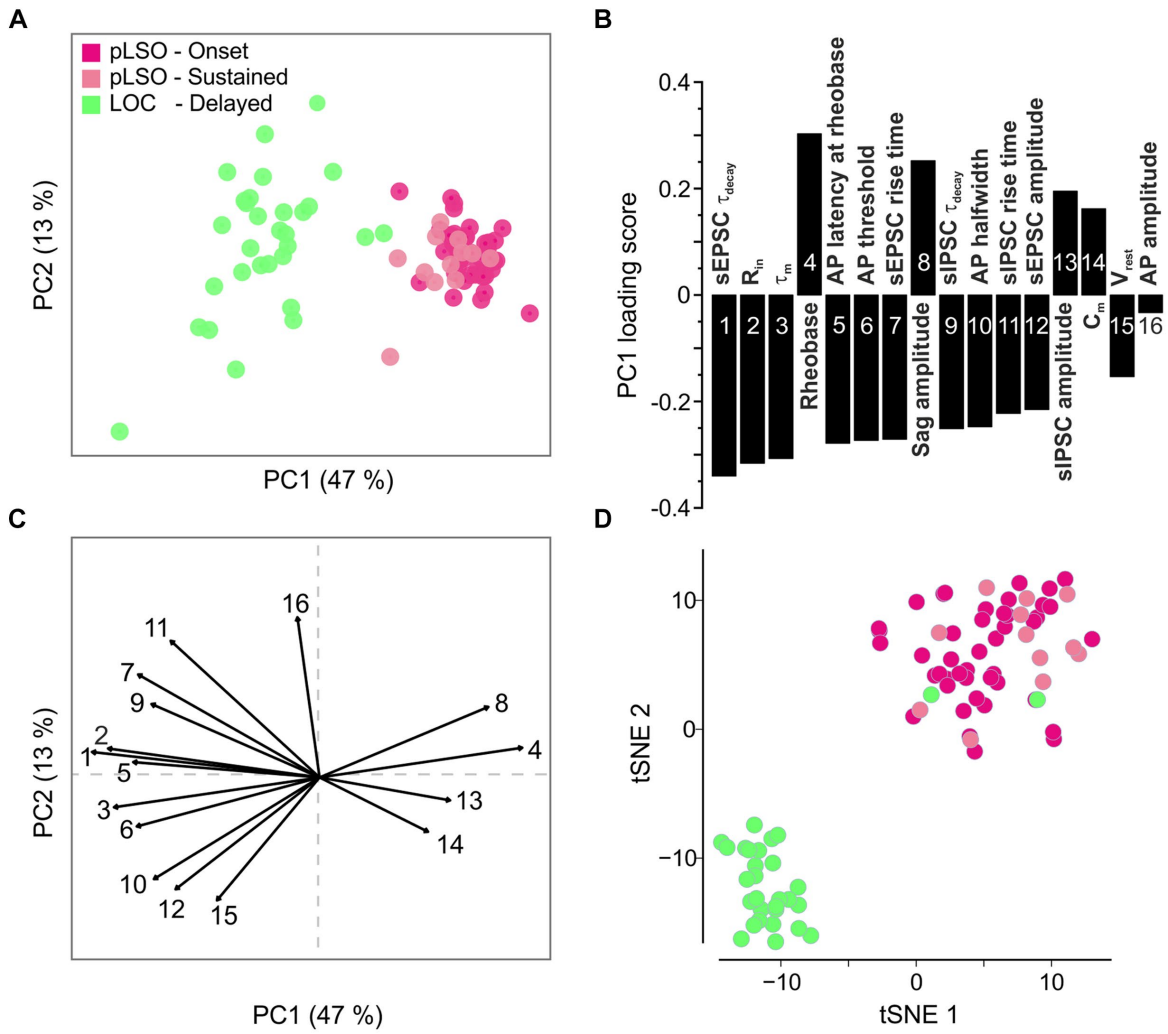
Principal component analysis (PCA) of electrophysiological features confirms the two clusters identified via transcriptomics. **(A)** PCA plot based on 16 electrophysiological features. **(B)** Loading scores for PC1 shown in **(A)**. **(C)** Vector factor map showing the features depicted in **(B)** and their contribution to PC1 and PC2. **(D)** Same tSNE plot as in Figure 3B, but color-coded according to the firing pattern (see also Supplementary Figures S6, S7).

The above electrophysiological characterization comprised 16 features. To reduce the dimensionality of the multivariate data and to search for potentially novel subclusters present in our electrophysiological data sets, we performed a PCA based on the 16 electrophysiological features. In such analyses, novel subclusters may arise due to a combination of differences that need not be significant, but together reveal distinct cell types. The results were in accordance with—and thus confirmed—the two clusters of LSO neurons identified via mRNA sequencing. The two subtypes of pLSOs (Onset and Sustained) clustered together (Figure 7A). The PCA also showed higher heterogeneity for cluster 2 than for cluster 1 where virtually all neurons were spotted in close vicinity to each other.

PC1 explained almost 50% of the cluster formation (Figure 7A). To determine the contribution of each feature to PC1, we evaluated the loading scores (Figure 7B). Four of the 16 features described time-related PSC characteristics (feature 1, 7, 9, 11). Thus, specific kinetics of both excitatory and inhibitory ligand-gated receptors appear to be a solid physiological means to distinguish pLSOs from LOCs (faster values in pLSOs). Moreover, R_in_, feature 2 on the loading score, may also be used to distinguish the two major types of LSO neurons rapidly and easily during electrophysiological characterization (*cf.* Supplementary Figure 6D). The lowest influence on PC1 was provided by the AP amplitude, followed by V_rest_ (Figure 7B; Supplementary Figures S6C,H). A vector factor map analysis showed a positive correlation of rheobase, sag amplitude, sIPSC amplitude, and C_m_ with PC1, whereas all other features were negatively correlated (Figures 7B,C). Interestingly, PC2 was mainly influenced by the AP amplitude and V_rest_, with both vectors being almost antiparallel to one another.

To assess a linkage of the three types of firing pattern with the gene expression pattern, we reused the tSNE plot from Figure 3B and mapped the data from the electrophysiological clusters onto the corresponding neurons (Figure 7D). Remarkably, there was absolute congruency between the transcriptional cluster assignment and the electrophysiological parameters. Hence, we feel safe to conclude that cluster 1 represents pLSOs and cluster 2 LOCs. Within the pLSOs, we identified two subtypes by their biophysical differences.

### pLSOs and LOCs express different sets of ion channel genes

**Figure 8:**
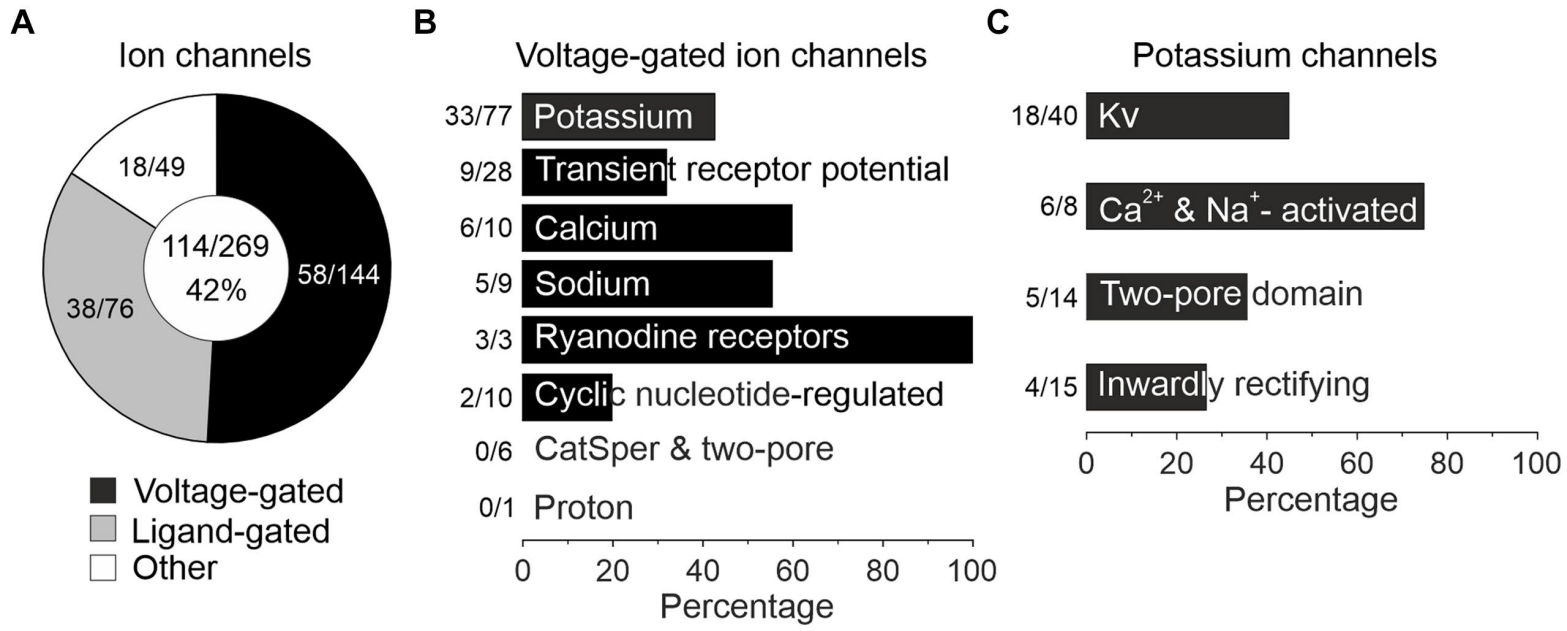
pLSOs and LOCs express a wide array of ion channels. **(A)** Forty-two percent (114/269) of ion channel genes (voltage-gated, ligand-gated, other) listed in the IUPHAR/BPS Guide to Pharmacology are expressed in the LSO. **(B)** Across the eight family members of voltage-gated ion channels, genes encoding potassium channels are most highly represented (43%; 33/77 genes). **(C)** The majority of potassium channel genes encode Kv channels (45%, 18/40).

Auditory brainstem neurons possess a plethora of morphological and functional features as well as molecular specializations that enable them to reliably process information in an ultrafast and temporally precise way (Oertel, 1997; Trussell, 1997, 1999; Golding and Oertel, 2012). Among morphological features are the well-known presynaptic terminals of the calyx-or endbulb-type (Held, 1893). Functional features include AP firing behavior and synaptic fidelity. Molecular specializations comprise an elaborate repertoire of voltage-gated ion channels (Hardman and Forsythe, 2009; Oertel, 2009; Reijntjes and Pyott, 2016). Of the 269 mouse genes for ion channels listed in the International Union of Basic and Clinical Pharmacology/British Pharmacological Society Guide to Pharmacology (IUPHAR/BPS GTP) (Alexander et al., 2021), our sample expressed 42% (114/269; Figure 8A; Supplementary Table S6 for cohort-related results). More specifically, 40% of voltage-gated ion channel genes were expressed (58/144; Figure 8A). For ligand-gated and other ion channels, values were 50 and 36%, respectively (38/76 and 18/49). Dividing the category “Voltage-gated ion channels” into eight subdivisions revealed expression of 43% of the potassium channels, by far the highest number (33/77; Figure 8B). Together, the results illustrate a broad repertoire of ion channels in LSO neurons, particularly potassium channels.

### Potassium channels

As potassium channels are crucial determinants of multiple physiological properties (V_rest_, AP threshold, firing rate, AP duration, postsynaptic potential duration, timing), and because of the immense interest auditory brainstem neurons have received concerning those channels, we focused on this subcategory (Figure 8C). Among 33 expressed K^+^ channel genes, 18 encode pore-forming Kv αsubunits (18/40 = 45%), six Ca^2+^- or Na^+^-activated (KCa or KNa) channels (6/8 = 75%), five two-pore domain K2P channels (5/14 = 36%), and four inwardly rectifying Kir channels (4/15 = 27%).

**Figure 9:**
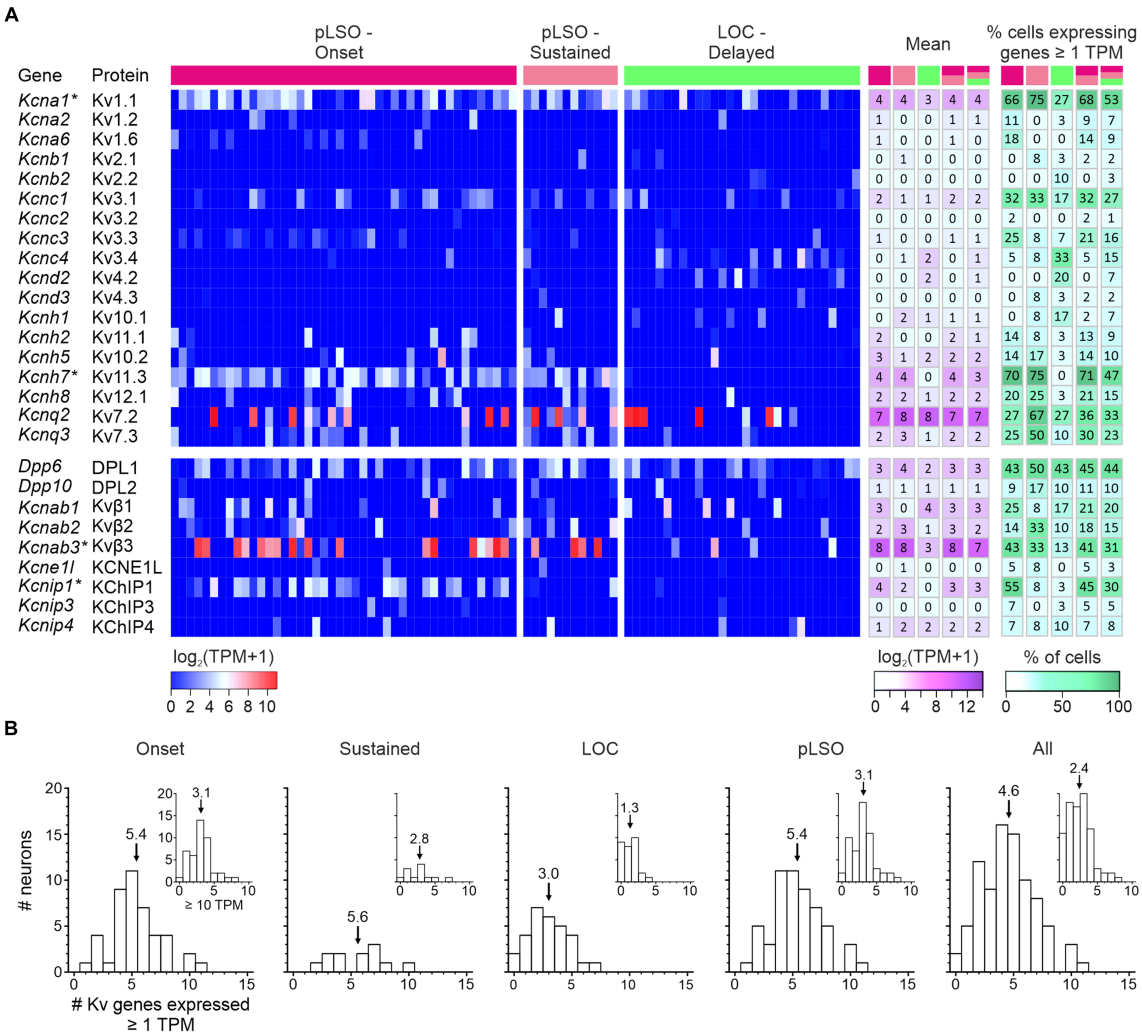
Expression of genes encoding voltage-gated potassium (Kv) α subunits and regulatory subunits (*n* = 18, 9). **(A)** Heat maps show expression levels [log_2_(TPM + 1)] for single neurons (three cohorts). To the right, the mean and the percentage of cells expressing a given gene are depicted for five cohorts (pLSO—Onset, pLSO—Sustained, LOC—Delayed, pLSO, all neurons; *n* = 44, 12, 30, 56, 86). DEGs are marked with asterisks. **(B)** Frequency histograms show distribution of gene expression for five cohorts (criteria: ≥ 1 TPM; ≥ 10 TPM for insets). Mean values are marked by arrows (see also Supplementary Table S6).

Transcript levels for the 18 Kv α subunits and the spread across single neurons are shown in Figure 9A and Supplementary Table S6. The panel also depicts expression for nine genes encoding “Potassium voltage-gated channel regulatory subunits” (of 14 listed,^5^ Gray et al., 2015). Overall, the LSO neurons expressed a high number of Kv genes (50%; 27/54). For five genes, the mean TPM values exceeded 10 (i.e., log_2_[TPM + 1] > 3.5) in at least one cluster (white-magenta heat map in Figure 9A; Supplementary Table S6). These genes were: pLSOs: *Kcnab3*|Kvβ3, *Kcnq2*|Kv7.2, *Kcna1*|Kv1.1, *Kcnh7*|Kv11.3; LOCs: *Kcnq2*|Kv7.2*, Kcnab1*|Kvβ1. Across all LSO neurons, the by far highest TPM values were seen for *Kcnq2* and *Kcnab3* (mean: 166 and 141). Four Kv genes qualified as DEGs in pLSOs (marked by asterisks in Figure 9A; pLSOs: *Kcna1*|Kv1.1, DEG#92; *Kcnh7*|Kv11.3, DEG#6; *Kcnab3*|Kvβ3, DEG#146; *Kcnip1*|KChIP1, DEG#67). We also determined the percentage of neurons expressing a given gene (white-green heat map in Figure 9A). *Kcna1*|Kv1.1 and *Kcnh7*|Kv11.3 were expressed by ~70% of cluster 1 neurons. For cluster 2, the percentage was relatively low: ~40% of the neurons expressed *Dpp6*|DPL1, the highest value observed (Figure 9A). Cluster 1 neurons displayed similarly high values for this gene.

We wondered how many Kv genes a single LSO neuron may express and assessed the heat maps vertically (threshold ≥1 TPM). On average, a single neuron expressed 17% of the Kv genes expressed in the whole sample (4.6/27; range: 0–11; Figure 9B). More specifically, values for the four cohorts were: 5.4 Onset; 5.6 Sustained; 3.0 LOCs (= cluster 2); 5.4 pLSOs (= cluster 1). Thus, pLSOs appear to express a 1.8-fold higher number of Kv genes than LOCs, implying higher complexity in the ascending LSO branch for this ion channel family. Taken together, pLSOs and LOCs show a broad and complex repertoire of Kv transcripts for α and regulatory subunits. Even within a cohort, molecular individuality seems to be a prominent feature. It appears that the complexity of the Kv channel profile in pLSOs is higher than in LOCs. Fifteen percent of the 27 Kv channel genes are DEGs, with no exception in pLSOs, emphasizing the importance of Kv channels particularly in this neuron type. To validate the gene expression results, we performed immunohistochemistry for four Kv subunits (next chapter). In one case, we also performed a series of pharmacological experiments.

### Immunohistochemical and pharmacological analysis of Kv7.2 and Kv7.3

**Figure 10:**
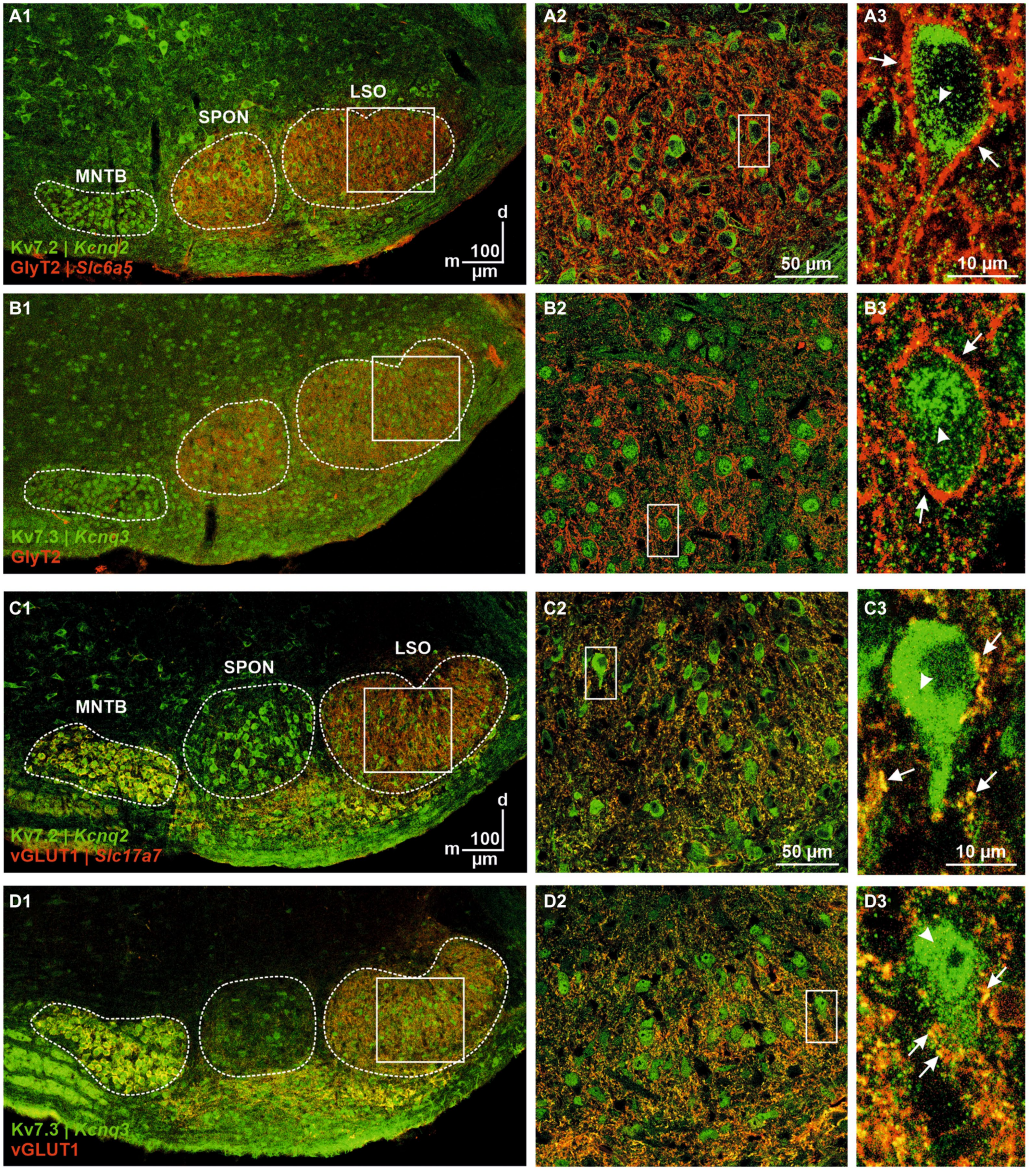
Immunohistochemical analysis of Kv7.2 and Kv7.3. **(A)** Kv7.2- (green) and GlyT2-immunolabeling (red) of the SOC **(A1)** and LSO **(A2)**. Coronal brainstem slice at P12. Close-up of a representative neuron in **(A3)**. Punctate glycinergic terminals surrounding the soma are marked by arrows; cytoplasmic Kv7.2 immunosignals are marked by an arrowhead. **(B)** As **(A)**, but for Kv7.3. **(C)** Kv7.2- (green) and vGLUT1-immunolabeling (red) of the SOC **(C1)** and LSO **(C2)**. Coronal brainstem slice at P12. Close-up of a representative neuron in **(C3)**. Punctate glutamatergic terminals surrounding the soma are marked by arrows; cytoplasmic Kv7.2 immunosignals are marked by an arrowhead. **(D)** As **(C)**, but for Kv7.3.

Kv7.2 (aka KCNQ2) immunoreactivity was detectable in all major SOC nuclei, and labeling was predominantly somatic (Figures 10A1,A2). GlyT2 colabeling helped to identify pLSOs by their soma shape and the punctate perisomatic and peridendritic pattern described before (Figure 10A3; Friauf et al., 2019). Kv7.2 immunosignals were strong in the cytoplasm, whereas a neurons’ nucleus was virtually immunonegative. We also analyzed Kv7.3 immunoreactivity (Figure 10B). The overall pattern resembled that seen for Kv7.2, except that labeling was more prominent in the nucleus than in the cytoplasm (Figure 10B3). For both Kv7 subunits, we observed no co-localization with GlyT2 in the LSO (Figures 10A2,A3,B2,B3), indicating that Kv7.2 and Kv7.3 are not present in glycinergic axon terminals. In contrast, immunosignals for Kv7.2 and Kv7.3 co-localized with vGLUT1-immunopositive axon terminals contacting LSO neurons at the soma and in the neuropil (Figures 10C,D). Together, these results suggest that the two Kv7 subunits are specific for glutamatergic synapses and absent from glycinergic terminals. This difference between the ipsilateral excitatory and the contralateral inhibitory input may be of physiological relevance for transmitter release in the context of sound localization and should be addressed in future studies.

**Figure 11:**
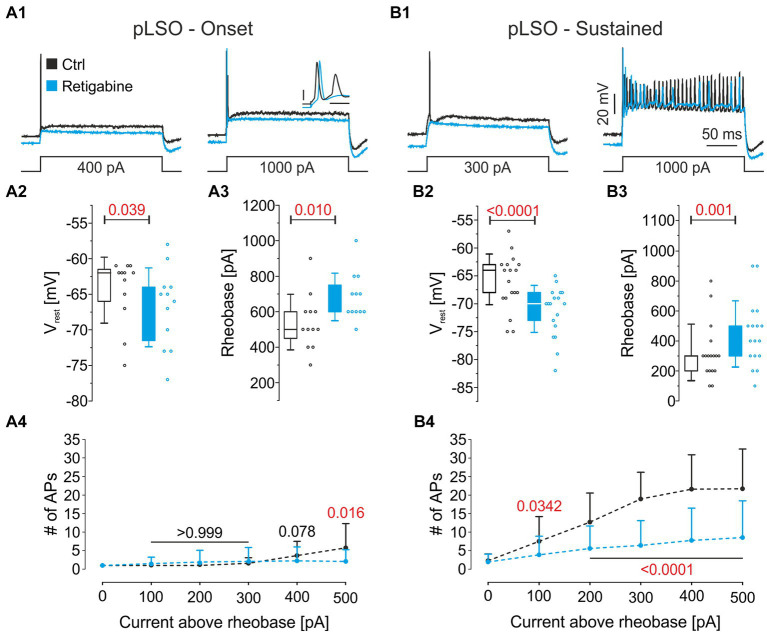
Pharmacological analysis of Kv7.2 and Kv7.3. **(A1)** Representative voltage traces from a pLSO—Onset neuron before (black) and after application of the Kv7.2/3 agonist retigabine (30 μM; light blue). Traces are in response to rectangular current pulses (left: control rheobase; right: 1,000 pA; 200 ms). Inset: close-up of the first 4 ms (scale bars: 20 mV; 2 ms). **(A2,A3)** Statistics for V_rest_ and rheobase in pLSO—Onset neurons. Open dots depict single neurons. **(A4)** Mean number of APs elicited as a function of the current amplitude above rheobase in pLSO—Onset neurons. Bars depict SD. Numbers depict *p*-values (in red when significant). **(B)** As **(A)**, but for pLSO—Sustained neurons (see also Supplementary Table S5).

To address functional Kv7.2/7.3 channels in pLSOs, we performed pharmacological experiments in which retigabine was applied while assessing biophysical properties from pLSOs. The drug is a Kv7.2/3 agonist that shifts channel activation toward more negative potentials (Main et al., 2000; Wickenden et al., 2000). In both pLSO subtypes, Onset and Sustained, retigabine hyperpolarized V_rest_ by ~4 mV (Figures 11A1,A2,B1,B2; Supplementary Table S5.2). Moreover, it increased the rheobase by ~30%, thus reducing multiple-firing behavior (Figures 11A1,A3,A4,B1,B3,B4). In summary, the immunohistochemical and pharmacological analyses validated our patch-seq data and obtained some interesting results concerning transmitter-type association. To our knowledge, this study is the first detailed report on Kv7.2 and Kv7.3 proteins in the LSO (*cf.* Cooper et al., 2001; for Kv7.5, see Caminos et al., 2007).

### Immunohistochemical analysis of Kv11.3 and Kvβ3

**Figure 12:**
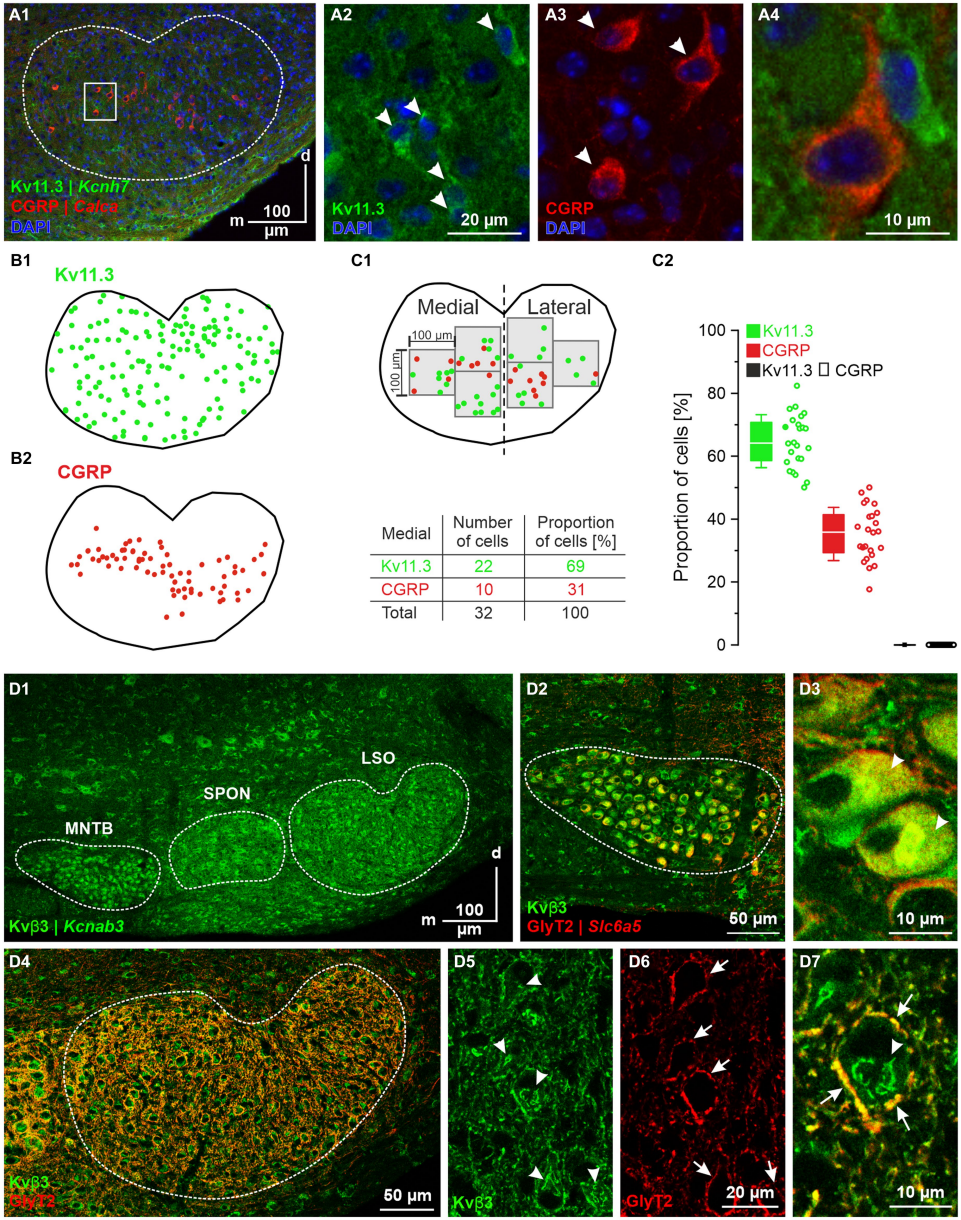
Immunohistochemical analysis of Kv11.3 and Kvβ3. **(A1–A4)** Kv11.3- (green) and CGRP-immunolabeling (red) in the LSO. Coronal brainstem slice at P12. Close-ups of representative neurons in **(A2–A4)**. Cell nuclei are DAPI-stained (blue). **(B1)** Distribution of Kv11.3-immunopositive somata (overlay from three sections). **(B2)** As **(B1)**, but for CGRP. **(C1)** Region of interest (3 squares, edge length: 100 μm) in the medial and lateral half of an LSO section in which immunopositive somata were counted. Table exemplarily shows number of somata obtained in the medial half. **(C2)** Statistics of the quantification shown in **(C1)**. The two solid dots relate to the example in **(C1)**. Kv11.3 ∩ CGRP depicts absence of double labeled somata. **(D1)** Kvβ3-immunolabeling (green) in the SOC. Coronal brainstem slice at P12. **(D2)** Kvβ3- and GlyT2-immunosignals (red) in the MNTB. **(D3)** Close-up of two representative MNTB neurons. Arrowheads mark cytoplasmic Kvβ3 signals. **(D4)** Double labeling for Kvβ3 and GlyT2 in the LSO. **(D5)** Five representative Kvβ3 immunopositive LSO neurons (marked by arrowheads). **(D6)** Perisomatic GlyT2-immunosignals (marked by arrows). Same neurons as in **(D5)**. **(D7)** Close-up of a double-labeled LSO neuron. Arrowhead marks cytoplasmic Kvβ3 signals; arrows mark presumptive MNTB axon terminals surrounding the soma. d, dorsal; m, medial.

We also validated expression for *Kcnh7*|Kv11.3 (DEG#6). It was expressed by ~70% of the pLSOs, whereas LOCs completely lacked expression (*cf.* Figure 9A). Double-immunofluorescent labeling of SOC sections for Kv11.3 and CGRP demonstrated Kv11.3 immunosignals throughout the LSO, whereas CGRP-positive cells were restricted to the center of the LSO, stretching along the tonotopic axis (Figures 12A1,B1,B2). For both proteins, immunosignals were localized in the somata and proximal dendrites (Figures 12A2–A4; see also Figure 5C3). In central aspects of the LSO, Kv11.3-positive somata outnumbered CGRP-positive ones ~2-fold (65% vs. 35%; Figure 12C2). Kv11.3 signals did not overlap with CGRP signals (Figures 12C1,C2), confirming the mRNA results and demonstrating exquisite cell-type specificity.

We finally performed immunohistochemical validation experiments for *Kcnab3*|Kvβ3 (DEG#146 in pLSOs). Kvβ3 immunolabeling was heavy in MNTB, SPON and LSO (Figure 12D1). The somata of MNTB neurons displayed intense immunosignals in the cytoplasm, but not in the nuclei. An opposite pattern occurred in LSO and SPON neurons (Figures 12D2–D7). LSO and SPON neurons displayed perisomatic labeling, suggesting the presence of Kvβ3-positive MNTB axon terminals. Indeed, double labeling demonstrated co-distribution of perisomatic Kvβ3 signals with GlyT2 signals, confirming presynaptic Kvβ3 in inhibitory synapses.

### Ca^2+^ and Na^+^-activated, two-pore domain, and inwardly rectifying Kv channels

Besides the Kv channels, three other classes of potassium channels are known (Figure 8C). They are encoded by 37 genes, and we found expression for 15 of them (Supplementary Table S6). In LOCs, *Kcnn3*|KCa2.3 and *Kcnj3*|Kir3.1 expression was high and moderate, respectively (427 vs. 30 TPM). pLSOs showed a high expression for *Kcnn1*|KCa2.1 and a moderate one for *Kcnj3*|Kir3.1 (134 vs. 18 TPM). None of the genes was a DEG. KCa2.1 and KCa2.3 form small-conductance, Ca^2+^-activated K^+^ channels (SK channels) that have high Ca^2+^ affinity and mediate the SK currents (Bond et al., 2005; Faber, 2009; Adelman et al., 2012). They are a major contributor to the afterhyperpolarization phase of APs and regulate AP firing, including AP precision (Engel et al., 1999; Hallworth et al., 2003). Their Ca^2+^ sensitivity is provided by CaM (Saimi and Kung, 2002), for which our study shows the third highest expression level among 11,659 genes (Figure 2B; Supplementary Table S3.2). SK2 channels in outer hair cells are activated via Ca^2+^ influx through ionotropic ACh receptors activated by cholinergic MOC neurons, ultimately resulting in hyperpolarization, and turning down the cochlear amplifier (Housley and Ashmore, 1991; Guinan, 2011; Maison et al., 2013). To our knowledge, nothing is known about the role of KCa2 channels in auditory brainstem neurons.

### Voltage-gated sodium (Nav) channels

Nav channels play a fundamental role in generating, shaping, and conducting APs (Catterall et al., 2005; de Lera Ruiz and Kraus, 2015; Hull and Isom, 2018). LSO neurons in total expressed nine Nav genes (Supplementary Figure S8A; Supplementary Table S6), of which five encoded α subunits and four regulatory β subunits (13 genes are listed altogether: Nav1.1—Nav1.9 plus Navβ1—Navβ4). Thus, 69% (9/13) of Nav genes were expressed in our sample. Mean expression levels of ≥10 TPM in at least one cluster were seen for four genes (pLSOs: *Scn1a*|Nav1.1, *Scn8a*|Nav1.6, *Scn1b*|Navβ1; LOCs: *Scn1a*|Nav1.1, *Scn3a*|Nav1.3, *Scn8a*|Nav1.6, *Scn1b*|Navβ1). Across all neurons, highest expression occurred for *Scn1b*|Navβ1, followed by *Scn8a*|Nav1.6, and *Scn1a*|Nav1.1 (mean TPM: 92, 47, 16). Three genes qualified as DEGs (pLSOs: *Scn4b*|Navβ4, DEG#173; LOCs: *Scn2a*|Nav1.2, DEG#49*; Scn3a*|Nav1.3, DEG#11). *Scn4b* transcripts were found in 25% of pLSOs and in not at all in LOCs. *Scn2a* was expressed by ~60% of LOCs and ~ 20% of pLSOs. Likewise, *Scn3a* was expressed by ~70% of LOCs and 20% of pLSOs.

At the single cell level, an average neuron expressed 33% (3.0/9) Nav genes displayed in the whole sample (range: 0–7; Supplementary Figure S8B). Corresponding values for the four cohorts were: 2.9 Onset; 2.9 Sustained; 3.2 LOCs [= cluster 2]; 2.9 pLSOs [= cluster 1]. Thus, ~3 Nav genes appear to be expressed per LSO neuron, regardless of the subtype, indicating similar numerical complexity. Nevertheless, the fact that 33% of the genes are DEGs, together with the broad percent range, implies a considerable amount of molecular individuality at the cellular level.

### Voltage-gated calcium (Cav) channels

Of 10 listed genes encoding Cav channel α subunits, six were expressed by the LSO neurons (Supplementary Figure S9A; Supplementary Table S6). In the group of regulatory subunits (α2δ, β, γ), we found expression for 12/16 genes. Collectively, LSO neurons expressed ~70% of the listed Cav genes (18/26). Therefore, gene expression for Cav channels was more elaborate than for Kv channels, but very similar to Nav channels (Kv: 50%; Nav: 69%). In general, expression levels were also considerably higher than for Kv or Nav subunits. Across all LSO neurons, mean TPM values exceeded 200 for Cav2.3, Cav2.2, and Cavβ4 (639, 372, 218), values not seen for Kv and Nav channels. Whereas mean TPM values ≥10 in at least one cluster occurred for five Kv and four Nav genes, 11 Cav genes fulfilled these criteria. On the α subunit side, these were *Cacna1b*|Cav2.2, *Cacna1d*|Cav1.3, *Cacna1e*|Cav2.3, and *Cacna1g*|Cav3.1. Across all LSO neurons, by far the highest mean TPM value was observed for Cav2.3, followed by Cav2.2 (1,491 and 372). For α2δ subunits, genes with ≥10 TPM were *Cacna2d2*|Cavα2δ2 and *Cacna2d3*|Cavα2δ3. For the β subunits, we identified *Cacnb1*|Cavβ1, *Cacnb3*|Cavβ3 and *Cacnb4*|Cavβ4, and for the γ subunits, we found *Cacng2*|Cavγ2 and *Cacng4*|Cavγ4. Only two Cav genes were DEGs, one gene per cluster (LOCs: *Cacna1e*|Cav2.3, DEG#26; pLSOs: *Cacng5*|Cavγ5, DEG#12). Concerning the percentage of cells, Cav2.3 was expressed by ~70% of the LOC cohort and Cavγ5 by ~65% of the pLSO cohort.

An average LSO neuron expressed 25% Cav genes (4.5/18; α plus regulatory subunits; range: 1–9; Supplementary Figure S9B). Corresponding values for the four cohorts were: 4.5 Onset; 5.4 Sustained; 4.3 LOCs [= cluster 2]; 4.7 pLSOs [= cluster 1]. The numbers indicate a similar complexity across clusters. In summary, a large fraction of Cav genes is expressed in our LSO sample (~70%). The expression profile differs substantially between pLSOs and LOCs as well as between the two pLSO subtypes. However, the similar number of expressed genes per neuron indicates similar complexity across cohorts.

### Transient receptor potential channels

Mammalian TRP channels form a superfamily comprising six families (Venkatachalam and Montell, 2007; Talavera et al., 2008). The patch-seq LSO neurons expressed ~$\frac{1}{3}$ of the listed TRP genes (9/28; Supplementary Figure S10A; Supplementary Table S6). Five of them displayed a mean of ≥10 TPM in at least one cluster, namely *Mcoln1*|TRPML1, *Pkd2l2*|TRPP5, *Trpm2*|TRPM2, *Trpm7*|TRPM7, and *Trpv2*|TRPV2. For three genes, TPM values were ≥ 100 in at least one cohort (TRPML1, TRPP5, TRPM2). The highest level (374 TPM) was observed for TRPML1 in Sustained neurons. None of the TRP genes was a DEG, implying only minor differences between both clusters. We found only very low transcript levels, if any, for TRPC channels. This slightly contrasts with earlier findings of mGluR-activated TRP-like channels that mediate Ca^2+^ influx in the developing LSO upon high-frequency stimulation (Ene et al., 2003, 2007). The authors concluded that these Ca^2+^ channels are likely TRPC channels.

An average LSO neuron expressed 1.4 TRP genes (5% of 28 listed; range: 0–5; Supplementary Figure S10B). Corresponding values for the four cohorts were: 1.2 Onset; 1.8 Sustained; 1.6 LOCs [= cluster 2]; 1.3 pLSOs [= cluster 1]. In summary, ~1/3 of all TRP genes (9/28) were expressed in our sample. There is a remarkable similarity between the two pLSO subgroups which was not evident for Kv, Nav, or Cav channels.

### Cyclic nucleotide-regulated channels

CNR channels translate concentration changes of cyclic nucleotides into electrical signals (Craven and Zagotta, 2006; Napolitano et al., 2021). They are closely homologous to Kv channels and form two families, HCN channels and cyclic nucleotide-gated (CNG) channels (Craven and Zagotta, 2006). In contrast to Kv, Nav or Cav channels, CNG channels are poorly ion selective and only weakly voltage dependent. CNG channels are tetramers formed by CNGA and CNGB subunits, whereas HCN channels are tetramers formed by four subunits (HCN1–4). The latter open with hyperpolarization, are weakly K^+^ selective, and are regulated by cyclic nucleotides, e.g., cAMP. Of 10 *Cng/Hcn* genes listed in the IUPHAR/BPS GTP (Alexander et al., 2021), LSOs exclusively expressed HCN channels (20%, 2/10; Supplementary Figure S11A; Supplementary Table S6). None qualified for a DEG. Mean expression levels for *Hcn2*|HCN2 were low (< 5 TPM); only *Hcn1*|HCN1 displayed a mean of ≥10 TPM. The highest percentage of neurons expressing a HCN gene was 50% in Onset neurons (white-green heatmap). The other cohorts displayed substantially lower values, particularly for HCN2.

An average LSO neuron expressed 20% of the HCN genes (0.4/2; range: 0–2; Supplementary Figure S11B). Corresponding values for the four cohorts were: 0.6 Onset; 0.1 Sustained; 0.3 LOCs; 0.5 pLSOs. Collectively, gene expression for HCN channels was not high in the LSO. However, HCN channel-mediated voltage sags have been described in pLSO—Onset and Sustained neurons (Leao et al., 2006). Our recordings confirm voltage sags in both pLSO types, but we find HCN channel expression only in Onset neurons. It is plausible that mRNA levels for HCN channels (and protein turnover) are too low in Sustained neurons to be detected. This is supported by a study indicating that HCN channels are sequestered within endosomes which can serve as an alternative reservoir for HCN surface expression, independent of their biosynthesis (Hardel et al., 2008).

### Ligand-gated ionotropic receptors (GluA, GluD, GluK, GluN, GlyR, GABAAR, nAChR)

**Figure 13:**
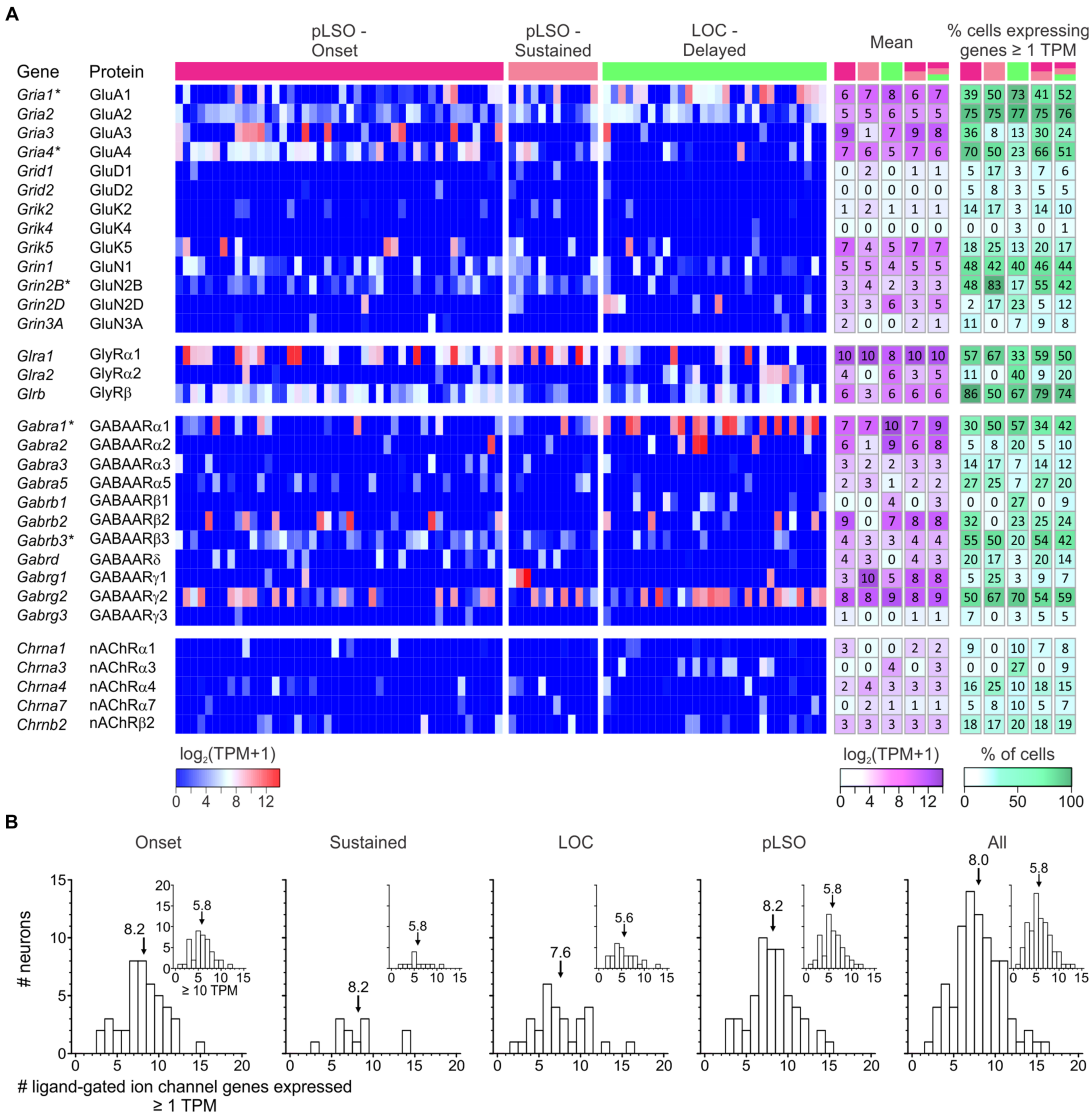
Expression of genes encoding ligand-gated ion channels (*n* = 32). **(A)** Heat maps show expression levels [log_2_(TPM + 1)] for single neurons (three cohorts). To the right, the mean and the percentage of cells expressing a given gene are depicted for five cohorts (pLSO—Onset, pLSO—Sustained, LOC—Delayed, pLSO, all neurons; *n* = 44, 12, 30, 56, 86). DEGs are marked with asterisks. **(B)** Frequency histograms show distribution of gene expression for five cohorts (criteria: ≥ 1 TPM; ≥ 10 TPM for insets). Mean values are marked by arrows (see also Supplementary Table S6).

LSO neurons receive fast synaptic input through multiple neurotransmitter systems targeting ligand-gated ion channels. Our electrophysiological data showed that the kinetics of excitatory and inhibitory sPSCs are solid means to distinguish pLSOs from LOCs (Figures 7B,C). To further assess this difference on the transcript level, we analyzed gene expression for subunits of six major channel types: glutamatergic AMPA, NMDA, kainate, and “orphan” receptors (GluA, GluN, GluK, GluD), glycine receptors (GlyRs), γ-amino-butyric acid receptors (GABAARs), and nicotinic acetylcholine receptors (nAChRs). We found expression for 55% of the receptor subunit genes (32/58 listed in IUPHAR/BPS GTP; Figure 13A). Thirteen (67%) of them code for ionotropic glutamate receptors, three for GlyRs, 11 for GABAARs, and five for nAChR subunits (Figure 13A; Supplementary Table S6; listed are 18, 5, 19, and 16). For 19 genes, the mean expression was ≥10 TPM in at least one cluster (15 in pLSOs, 17 in LOCs).

Across all 86 neurons, the highest expression occurred for *Glra1*|GlyRα_1_ (756 TPM), followed by *Gabra1*|GABAARα_1_, *Gabrg2*|GABAARγ2, *Gria3*|GluA3, and *Gabrb2*|GABAARβ2. In comparison, GluK5, GluN, or nAChR gene expression was substantially lower (90 TPM for GluK5, the highest value). Expression for four glutamate receptor subunits of the GluD and GluK subtype was generally low, and it was absent for 5 of 18 genes listed. Slightly higher expression levels were seen for nAChRs. Five genes turned out to be DEGs (pLSOs: *Gria4*|GluA4 DEG#40, *Grin2b*|GluN2B DEG#36, *Gabrb3*|GABAARβ3 DEG#72; LOCs: *Gria1*|GluA1 DEG#68, *Gabra1*|GABAARα1 DEG#89). The diversity emphasizes the importance of diverse ligand-gated ionotropic receptors in LSO neurons and points to specific functions, even within a cluster. This is highlighted by the percentage of pLSOs expressing a DEG:66% for *Gria4*, 55% for *Grin2b*, and 54% for *Gabrb3* (Figure 13A, white-green heatmap; 23, 17, and 20% for LOCs). Values in LOCs were 73% for *Gria1* and 57% for *Gabra1* (41 and 34% for pLSOs).

Concerning the question of how many genes encoding ligand-gated receptor subunits are expressed in a single LSO neuron, the mean number was 8.0 (range: 2–14; Figure 13B). Corresponding values for the four cohorts were: 8.2 Onset; 8.2 Sustained; 7.6 LOCs; 8.2 pLSOs, implying similar transcript complexity. Taken together, more than 50% of the subunits in ligand-gated ion channels are detectable at the mRNA level. The high number of DEGs (5 in 58) demonstrates clear differences between pLSOs and LOCs.

### G protein-coupled receptors (mGluR, GABABR, mAChR)

G protein-coupled receptors (GPCRs) constitute the largest family of cell-surface proteins involved in signal transmission (Rosenbaum et al., 2009). Upon agonist binding, they activate specific heterotrimeric G proteins which results in the modulation of distinct downstream effector proteins, including ion channels. The receptors are characterized by the presence of seven membrane-spanning segments in a monomer. We focused on three major types of GPCRs being activated by glutamate, GABA, or acetylcholine. For metabotropic glutamate receptors (mGluRs), there are three groups comprising eight members (group I: mGluR1, mGluR5; group II: mGluR2, mGluR3; group III: mGluR4, 6–8). Whereas group I receptors are primarily present at postsynaptic sites, group II and III mGluRs localize predominantly presynaptically. Metabotropic GABA receptors have two members (GABABR1 and GABABR2), whereas metabotropic ACh receptors (mAChRs) have five (M1—M5, encoded by *Chrm1—Chrm5*).

LSO neurons in general expressed 67% of the named GPCR genes (10/15; Supplementary Figure S12A; Supplementary Table S6). Four genes displayed a mean expression of ≥10 TPM in at least one cluster (GABABR1, mGluR1, mGluR3, mGluR8). By far the highest expression was seen for mGluR3 (all LSO: 521 TPM). In contrast, mGluR4 transcripts were almost absent, and those for mGluR2 and mGluR6 were undetected. Within GABABRs, transcripts prevailed for GABABR1 (25 TPM). Among the mAChRs, expression was absent for three genes and low for M2 and M4. Nevertheless, *Chrm2*|M2 was DEG#93 for LOCs (the only DEG in the CPCG group; TPM: 5 vs. 0). It was expressed by 27% of LOCs and 2% of pLSOs.

An average LSO neuron expressed 2.0 GPCR genes (range: 0–6; Supplementary Figure S12B). Corresponding values in the cohorts were: 1.5 Onset; 2.3 Sustained; 2.6 LOCs; 1.7 pLSOs. Together, the results demonstrate a higher GPCR complexity in LOCs.

### Neurotransmitter-associated genes (excitatory, inhibitory, modulating)

**Figure 14:**
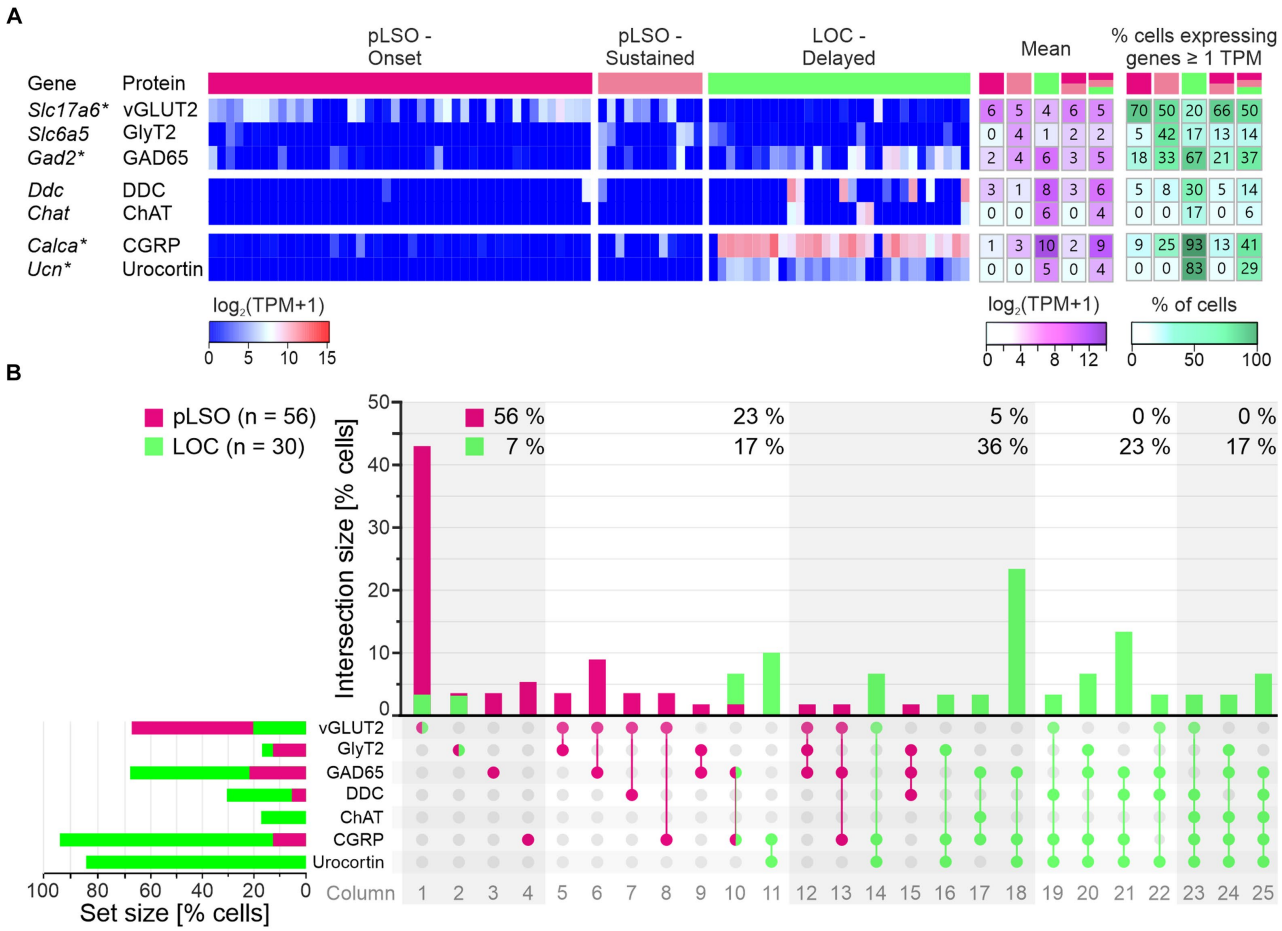
Expression of neurotransmitter-associated genes (*n* = 7). **(A)** Heat maps show expression levels [log_2_(TPM + 1)] for single neurons (three cohorts). To the right, the mean and the percentage of cells expressing a given gene are depicted for five cohorts (pLSO—Onset, pLSO—Sustained, LOC—Delayed, pLSO, all neurons; *n* = 44, 12, 30, 56, 86). DEGs are marked with asterisks. **(B)** UpSet plot shows percentage of neurons expressing genes for neurotransmitter-associated proteins in pLSOs (magenta) and LOCs (green). Vertical bars show the percentage of neurons expressing one or a combination of genes (Intersection size; 100% relates to 56 pLSOs and to 30 LOCs). Values at the top represent the sum of the gray/white shaded areas, indicating the percentage of concurrent expression of either one, two, three, four or five neurotransmitter-associated genes. Note that we did not find neurotransmitter-associated gene expression in all pLSOs. Dots (and connecting lines) mark where the overlap occurs. Horizontal bars (Set size) show the percentage of neurons expressing a given gene (see also Supplementary Table S6).

LSO neurons display a high degree of transmitter diversity (*cf.* Figure 1A). To explore the diversity at the single-cell level, we analyzed gene expression of a subset of neurotransmitter-associated molecules, including neuropeptides to explore neurotransmitter phenotype. Figure 14A and Supplementary Table S6 illustrate seven neurotransmitter-associated genes expressed in the LSO (*Slc17a6*|vGLUT2; *Slc6a5*|GlyT2; *Gad2*|GAD65; *Ddc*|DOPA decarboxylase = DDC; *Chat*|choline acetyltransferase = ChAT; *Calca*|CGRP; *Ucn*|urocortin). The highest expression was seen for CGRP (LOCs: 1,256 TPM), followed by DDC (LOCs: 231 TPM), GAD65 (LOCs: 87 TPM), and vGLUT2 (pLSOs: 59 TPM). Four genes qualified for DEGs (LOCs: *Calca*|CGRP DEG#1; *Ucn*|urocortin DEG#2; *Gad2*|GAD65 DEG#32; pLSOs: *Slc17a6*|vGLUT2 DEG#26). Transcripts for vGLUT2, indicative of a glutamatergic phenotype, were found in 66% of the pLSOs, and transcripts indicative of a glycinergic and GABAergic phenotype in 13 and 21%, respectively (Figure 14A, white-green heatmap). Thus, the predominant transmitter in pLSOs seems to be glutamate. The opposite appears to hold for LOCs, as 67% of them showed transcripts for GAD65 and 17% for GlyT2, whereas only 20% were associated with a glutamatergic phenotype (sum >100% because of multi-transmitter neurons; see below). Our results also indicate an excitatory, rather than an inhibitory, phenotype for most Onset neurons (70% vGLUT2, only 5% GlyT2 and 18% GAD65), whereas Sustained neurons appear to substantially utilize inhibitory transmitters (42% GlyT2 plus 33% GABA vs. 50% vGLUT2). Reportedly, membrane properties of excitatory pLSOs favor integrative level coding, whereas those of inhibitory pLSOs favor time coding (Haragopal and Winters, 2023).

As the above results implied multi-transmitter properties, we analyzed our samples in more detail. In total, the 86 LSO neurons displayed 25 combinations of neurotransmitter-associated gene expression (Figure 14B, columns 1–25; see also Supplementary Table S6). Of those, 13 combinations were seen in pLSOs and 15 combinations in LOCs. A single transmitter phenotype was obvious in 56% of the pLSOs (columns 1–4). Forty-three percent of the pLSOs appeared to be purely glutamatergic (column 1), whereas 23% co-expressed one to two neurotransmitter-associated genes in addition to vGLUT2 (columns 5–8, 12, 13). Thus, glutamate was by far the predominant transmitter type in pLSOs (66%; see Set size diagram). The remaining 34% were vGLUT2 negative (columns 2–4, 9, 10, 15). Seven pLSOs (12.5%) expressed the CGRP gene, which was a bit surprising because the neuropeptide is usually affiliated with LOCs. Transcripts for ChAT and urocortin were completely absent from the pLSO cohort. The maximum number of neurotransmitter-associated genes expressed in an individual pLSO was three (columns 12, 13, 15; 9% of the neurons).

LOCs displayed a substantially higher degree of multi-transmitter properties than pLSOs. Seventeen percent of them co-expressed five genes (Figure 14B, columns 23–25). Forty percent of the LOCs co-expressed >3 genes (columns 19–25), whereas no pLSO did so. On the other side, only 7% of the LOCs displayed a single-transmitter phenotype (Figure 14B, columns 1, 2; vs. 56% pLSOs). The dominant subtype of LOCs (23%) co-expressed genes coding for GABA, CGRP, and urocortin (column 18). Across all combinations, 93 and 83% of the LOCs showed transcripts for CGRP and urocortin, respectively (see Set size diagram). This pair of neuropeptides is clearly a hallmark of LOCs. Transcripts for GAD65 occurred in 67% of the LOCs. They were always associated with co-expression, in 10 neurons for three to four additional genes (columns 20–22, 24, 25). Thirty percent of LOCs expressed *Ddc*|DDC, indicative of a dopaminergic phenotype (vs. 6% of pLSOs). *Ddc* expression was always associated with three to four other transcripts, in each case for CGRP and urocortin. At a frequency of 17% (3 neurons), LOCs expressed *Chat*, a surprisingly low number (see Discussion for explanation). *Chat* expression was invariably flanked by CGRP transcripts and in some cases also with GAD65 (columns 17, 24, 25) and/or urocortin transcripts (columns 23–25).

An average LSO neuron expressed 1.9 neurotransmitter-associated genes (range: 0–5; Supplementary Table S6). Corresponding values for the four cohorts were: 1.1 pLSO—Onset; 1.6 pLSO—Sustained; 3.3 LOCs; 1.2 pLSOs. The almost 3-fold higher value in LOCs compared to pLSOs further highlights the complex transmitter repertoire in the LOC system. Collectively, the results on neurotransmitters add further facets to the overall picture that pLSOs and LOCs differ considerably in their transmitter repertoire. Altogether, the UpSet plot in Figure 14B shows that only 3 of the 25 combinations are shared between the two clusters (columns 1, 2, 10). This implies a very clear distinction between pLSOs and LOCs concerning their transmitter repertoire. pLSOs are characteristic in that they express mainly glutamatergic or GABAergic genes. In contrast, LOCs display multi-transmitter properties, most frequently GABA plus two neuropeptides.

### Super DEGs and cluster similarity between pLSOs and LOCs

**Figure 15:**
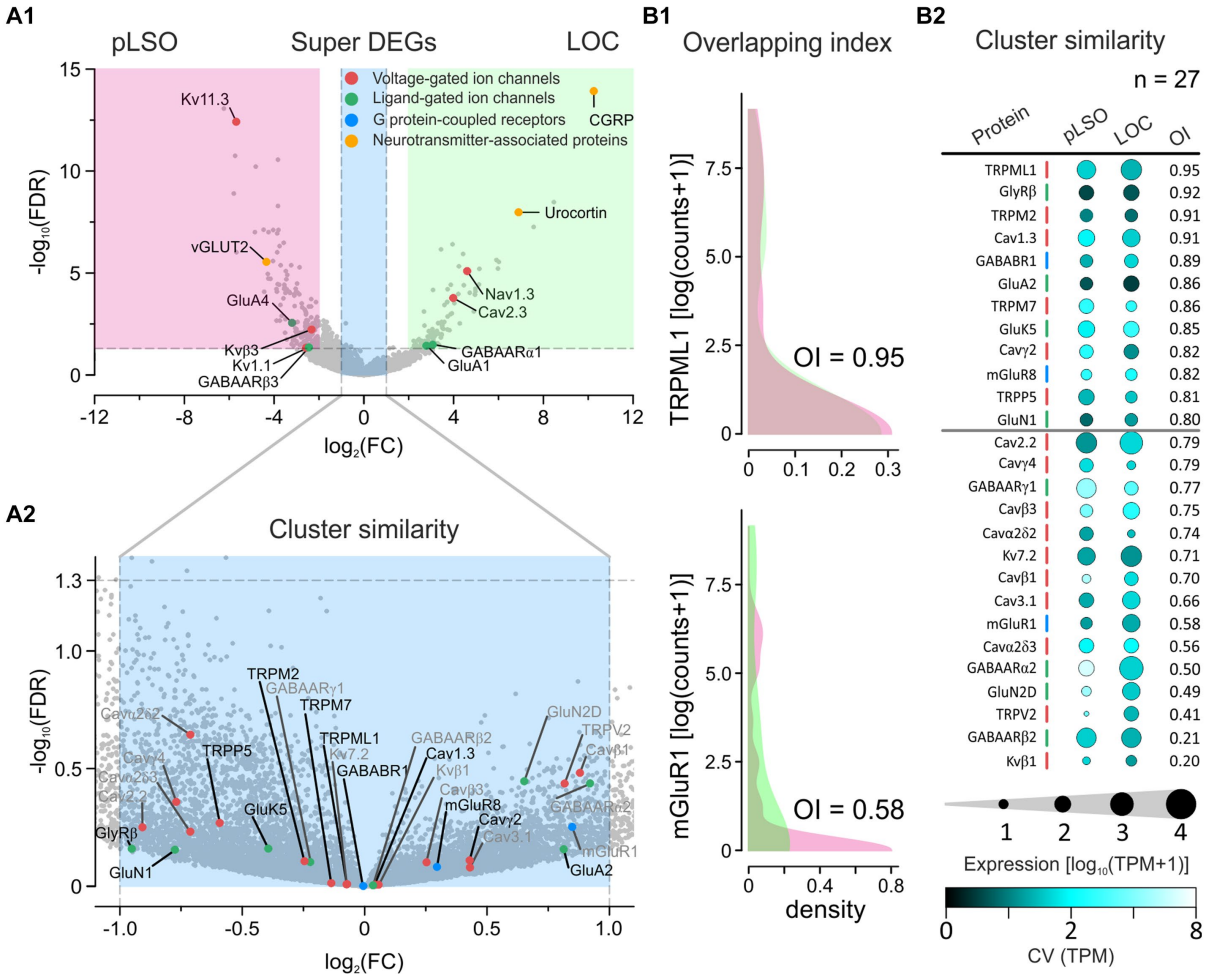
Super DEGs and cluster similarity. **(A1)** Volcano plot depicting “Super DEGs” involved in neurotransmission (set from Figure 4A). Four categories were assessed, together comprising 114 genes: Voltage-gated ion channels, Ligand-gated ion channels, G protein-coupled receptors, Neurotransmitter-associated proteins. Criteria for cluster specificity: expression level ≥ 10 TPM in at least one cluster, FC ≥ 4, red and green sector. For the 12 Super DEGs, the protein name is provided. **(A2)** Volcano plot depicting “Cluster similarity” (criteria: expression level ≥ 10 TPM in at least one cluster, FC ≤ 2; blue sector). For the 27 genes demonstrating cluster similarity, the protein name is provided [in gray if overlapping index (OI) < 0.8]. **(B1)** Density distributions for two example genes and the resulting OI. Distribution for pLSOs in magenta, for LOCs in green. **(B2)** Ranking the 27 genes by their OI. In the balloon plot, the dot size indicates TPM and the color code the coefficient of variance (CV). High OIs occur if dot size and colors are similar between clusters. Congruent density distributions yield an OI of 1.0. Colored vertical bars mark the same category shown in **(A1,A2)**. The horizontal line depicts the arbitrary threshold of 0.8 (see also Supplementary Table S6).

In a final analysis, we assessed “Super DEGs” and “Cluster similarity” (details in Methods). We restricted the analysis to the field of neurotransmission, namely voltage-gated ion channels, ligand-gated ion channels, GPCRs, and neurotransmitter-associated proteins (Figure 15). The input comprised 114 genes, of which 18 were DEGs. About 10% of the genes (12/114) were Super DEGs (6 in each cluster; Figure 15A1). Five of them encode subunits of voltage-gated ion channels (pLSOs: Kv11.3, Kvβ3, Kv1.1; LOCs: Nav1.3, Cav2.3), four encode subunits of ligand-gated ion channels (pLSOs: GluA4, GABAARβ3; LOCs: GABAARα1, GluA1), and three genes encode neurotransmitter-related proteins (pLSOs: vGLUT2; LOCs: CGRP, urocortin). No Super DEG was revealed for GPCRs.

Cluster similarity was found for ~25% of the genes (27/114; Figure 15A2). We further quantified this finding by determining an overlapping index (OI) (Pastore and Calcagni, 2019). OI values ranged from 0.95 to 0.2 and allowed to rank the genes. When the stringency was increased (by setting the OI threshold to 0.8), 12 genes remained. Six of them encode subunits of voltage-gated ion channels (Cav1.3, Cavγ2, TRPML1, TRPM2, TRPM7, TRPP5), four encode subunits of ligand-gated ion channels (GlyRβ, GluA2, GluK5, GluN1), two encode GPCRs (GABABR1, mGluR8), and none encodes neurotransmitter-related proteins.

Taken together, ~80% (90/114) of the genes involved in neurotransmission are neither Super DEGs nor do they fulfill the stringent criteria for Cluster similarity. The remaining 24 genes categorize equally to Super DEGs and Cluster similarity (12 + 12). Moreover, the 12 Super DEGs are equally split between pLSOs and LOCs. Thus, we crystallized three manageable sets of neurotransmitter-associated genes whose expression pattern differs between pLSOs and LOCs or, on the other hand, is closely similar.

## Discussion

**Figure 16:**
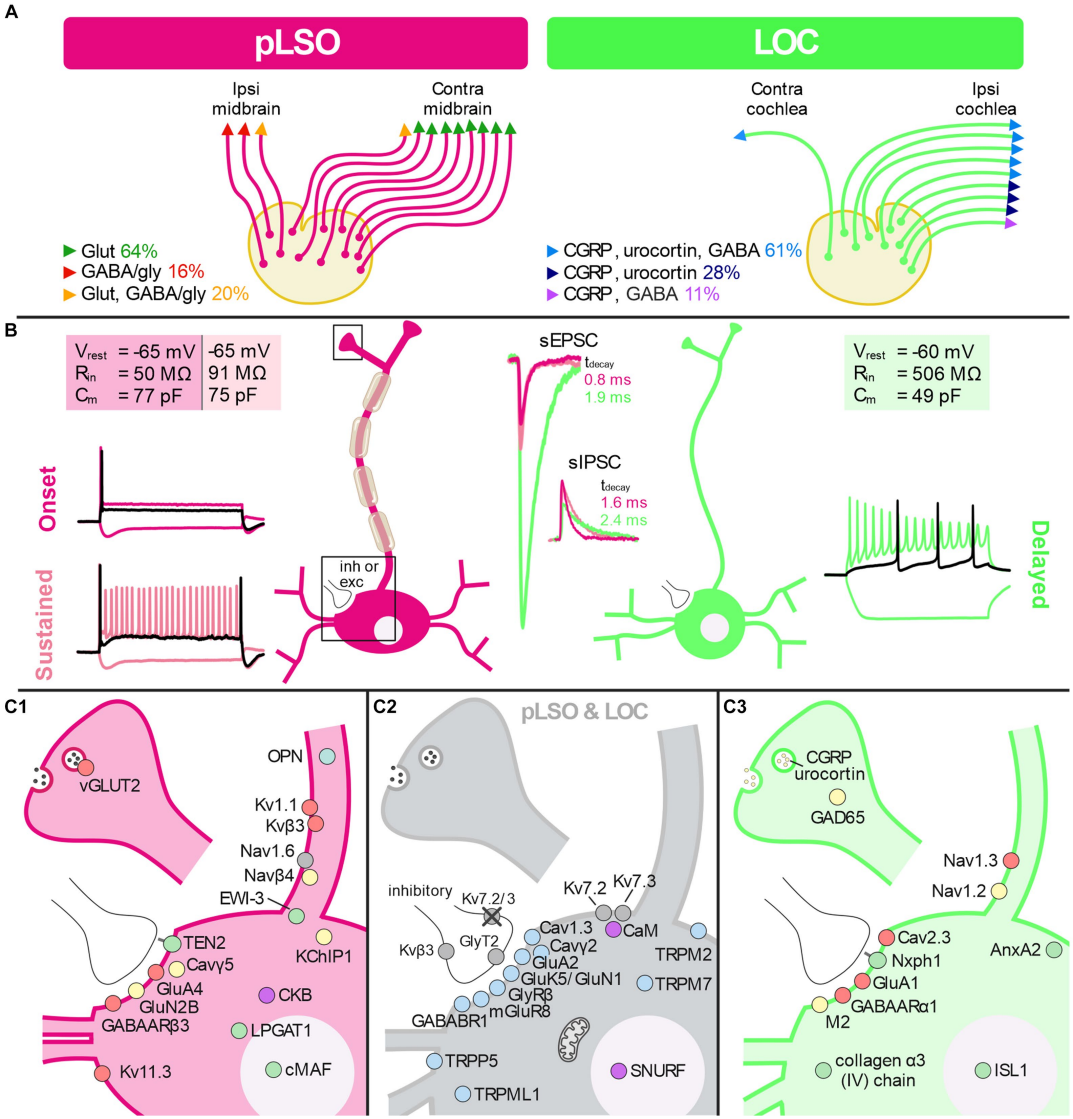
Graphical summary of the major results from the present study. Schemata depict differences and similarities between pLSOs and LOCs. **(A)** Gene expression for the three classical transmitters glutamate, glycine, and GABA demonstrates an excitatory phenotype in 64%, an inhibitory in 16%, and a mixed excitatory + inhibitory in 20% of pLSOs. In LOCs, the prevailing three transmitter profiles are CGRP + urocortin + GABA, CGRP + urocortin, and CGRP + GABA (61, 29, 11%). Overall, the transmitter repertoire of LOCs is more complex (see also Figure 13 and Supplementary Table S6). **(B)** Each subtype of pLSOs (onset or sustained firing) has a slightly lower V_rest_, a drastically lower R_in_, and a slightly higher C_m_ than LOCs. Moreover, each subgroup displays sag behavior. LOCs show a delayed firing pattern and no sag behavior. pLSOs have fusiform somata and thick, myelinated axons, whereas LOCs have round somata and thin, unmyelinated axons. Postsynaptic currents (sEPSCs and sIPSCs) show considerably faster kinetics in pLSOs than in LOCs (notice τ_decay_) (see also Figure 6 and Supplementary Figures S6, S7). **(C)** Cluster-specific genes (pLSO vs. LOC) as well as genes displaying inter-cluster similarity (pLSO and LOC). For the sake of clarity, the three panels depict only selected gene products (pLSO: 17, both: 18, LOC: 13). **(C1)** For pLSOs, we plotted the end products of six Super DEGs (red), five top DEGs (green), four DEGs involved in neurotransmission (yellow), the top one highly expressed gene (purple), and Nav1.6 (gray) (see also Figures 2–4, 5–9, 11–15). **(C2)** Across both neuron types, we plotted the end products of 12 genes demonstrating Cluster similarity (light blue), top two and top three highly expressed genes (purple), and four proteins for which we obtained confirmative results via immunohistochemistry or pharmacology (gray). The mitochondrion represents genes involved in ATP synthesis (see Figure 2E; see also Figures 2, 9–15). **(C3)** For LOCs, we plotted the end products of six Super DEGs, six top DEGs, and three DEGs involved in neurotransmission (same color code as in **(C1)**, see also Figures 4, 5, 12–15). The scenario in **(C1–C3)** is hypothetic as schemata show proteins at their presumptive location. glut, glutamate; gly, glycine; inh, inhibitory; exc, excitatory.

Brain function depends on the action of individual neurons. Neurons are relatively easy to track when a brain region consists of a homogeneous population of neurons (e.g., a motor nucleus). The scenario is much more complex and complicates analysis when the area of interest is a heterogeneous nucleus, such as the LSO. Here we characterized different neuron types of the juvenile mouse LSO using the patch-seq method to identify transcriptional differences and similarities. By choosing a juvenile age (at the onset of hearing), we were able to capture genes that are relevant for maturation. We obtained five main results (graphically summarized in Figure 16). (1) LSO neurons show high gene expression for ATP synthesizing proteins (Figure 2). (2) There are two prominent clusters: pLSOs and LOCs, the major types involved in the ascending (afferent) and descending (efferent) auditory pathways, respectively (Figure 3). (3) pLSOs and LOCs differ in 353 DEGs (254 + 99; Figure 4A). (4) Among the top 40 DEGs (20 in each cluster), 80% were hitherto undescribed (17 in pLSOs; 15 in LOCs; bold in Figure 4B). (5) Among the 114 listed genes involved in neurotransmission, 12 (~10%) are Super DEGs, of which three encode Kv channel subunits typical for pLSOs (Figure 15). Another 12 show Cluster similarity, highlighting the overlap of neurotransmission-related molecules between the two neuron types.

### Extraordinarily high expression for three genes: *Ckb, Snurf*, *Calm1*

The three most highly expressed genes showed expression levels of ~25,000 TPM and more, >2-fold higher than the fourth gene (Figure 2). The related proteins are brain-type creatine kinase B (CKB), SNRPN upstream open reading frame protein (SNURF; SNRPN = small nuclear ribonucleoprotein polypeptide N), and calmodulin 1 (CALM1 also known as CaM).

CKB is involved in energy homeostasis. The cytosolic enzyme transfers phosphate between ATP and various phosphogens, for example creatine, thus generating phosphocreatine and ADP and establishing a readily available ATP-buffering system. It is not surprising that *Ckb* expression correlates very well with gene expression for Na^+^/K^+^-ATPase α1 and α3 subunits (Jiao et al., 2021; NB: α3 is exclusive to neurons). The CKB-mediated buffer system appears to play a pivotal role in the hair bundle of vestibular hair cells (Shin et al., 2007). Reduced CKB activity is associated with hearing impairment in mice with Huntington disease (Lin et al., 2011). Interactions of CKB with the potassium-chloride transporter KCC2 have been demonstrated (Inoue et al., 2004, 2006; Li et al., 2007; Salin-Cantegrel et al., 2008). The interactions facilitate KCC2-mediated Cl-extrusion from neurons and maintain the low intracellular Cl-concentration required for effective inhibitory neurotransmission, i.e., hyperpolarization (for LSO, Balakrishnan et al., 2003). As robust inhibitory input to LSO neurons is crucial for sound localization, high CKB abundance is not surprising. Indeed, high *Ckb* expression in the LSO is also shown in the AMBA (Figure 2D), thus validating our data.

SNURF is a 71 amino acid-long polypeptide encoded by the two-gene locus “s*nurf/snrpn*.” This locus also encodes the small nuclear ribonucleoprotein (associated) polypeptide N (SNRPN) (Ozçelik et al., 1992). Across seven tissues, SNRPN is most abundant in the brain (rat; McAllister et al., 1988). Extensive alternative splicing and promoter usage occurs for SNURF and SNRPN, and the full-length nature of several transcripts remains to be determined. Like other imprinted genes, the *SNURF/SNRPN* domain is involved in neurodevelopment (Ribeiro Ferreira et al., 2019). Developmentally related changes of DNA methylation have been described for the mouse gene (Miyazaki et al., 2009). Loss of imprinted gene expression leads to disorders, e.g., severe intellectual disability (Nicholls et al., 1998). Presumably, SNURF plays a role in pre-mRNA splicing, and in line with this, SNURF is localized to the cell’s nucleus. Despite decades of research, the biological role of SNURF is not fully understood (Basak and Basak, 2022). A Pubmed search (“snurf brain” and “snurf neuron”; 2023–10-22) revealed 14 and nine results, respectively. None was associated with papers on the auditory system, the brainstem, or the cortex. Taken together, our study discovered *Snurf* as a novel gene that probably plays an important role in the CAS. The intermediate expression level for *Snurf* in the LSO and other auditory brainstem regions, as demonstrated in the AMBA, strengthens our conclusion.

CaM is one of three calmodulin proteins in the family of EF-hand Ca^2+^-binding proteins. These ubiquitous proteins exhibit a high degree of evolutionary conservation (Wren et al., 2019). CaM-Ca^2+^ complexes mediate the control of many structurally different proteins, such as voltage-gated ion channels (e.g., Cav1.3: Johny et al., 2013; Cav2.1: Lee et al., 2002; Jiang et al., 2008; KCa2: Bildl et al., 2004; Kv7.1: Sachyani et al., 2014; Kv7.2: Alaimo and Villarroel, 2018; Nav1.5: Gabelli et al., 2014). CaM appears to be the most relevant transducer of Ca^2+^ signals (Levitan, 1999; Faas et al., 2011). Moreover, irrespective of the intracellular Ca^2+^ concentration, CaM is constitutively tethered to Kv7.2/3 channels and presumably plays a key role in Kv7 channel folding and trafficking (Wen and Levitan, 2002; review: Soldovieri et al., 2011). Notably, the present study demonstrates substantial gene expression for Kv7.2 and Kv7.3 (Figures 9, 10), pointing to such an interaction.

In the CAS, mostly analyzed at calyx of Held synapses, CaM controls several forms of synaptic plasticity (Nakamura et al., 2008; Lee et al., 2010; Lipstein et al., 2013). *Calm1* expression increases significantly upon tone rearing in auditory cortex interneurons, implying a role in experience-and activity-dependent circuit refinement during the tonotopic critical period (Kalish et al., 2020). We are not aware of a CaM study in the LSO. Based on our results, we suggest investigating interactions between CaM and Kv7.2/3 channels in pLSOs. Consistent with our findings, the AMBA shows intermediate to high expression levels in the LSO (Figure 2D).

### Energy metabolism: ATP synthesis

Besides *Ckb*, 32 other genes among the top 500 were involved in ATP synthesis (Figure 2). The prevalence of transcripts for catabolic pathways is consistent with classic reports of high energy consumption in auditory brainstem neurons that fire APs at particularly high rates (e.g., Sokoloff, 1981). Previous reports on the LSO have focused on a few metabolic proteins (Trattner et al., 2013; Brosel et al., 2018). Our study is the first to comprehensively address LSO neurons and link ATP demand to a variety of specific enzymes. In a recent scRNA-seq study in the mouse cochlea, 33 genes among the top 500 in class I spiral ganglion neurons were associated with energy metabolism (Petitpré et al., 2018). Nine of them overlapped with the 33 genes detected in LSO neurons, all of them in the OxPhos group (*Ndufa11*, *Ndufs2*, *Ndufs3*, *Sdhb*, *Sdhd*, *Cox5a*, *Atp5b*, *Atp5f1*, *Atp5g3*). The overlap of ~30% indicates similarity between the two groups of auditory neurons, but also considerable dissimilarity, as previously shown for mitochondrial molecules (Fecher et al., 2019). In this lineage, only 3 of the 33 genes were DEGs (cluster 1: *Pfkp*, DEG#21, *Ckb*, DEG#254; cluster 2: *Acly*, DEG#10), suggesting a similar efficiency of ATP synthesis in pLSOs and LOCs. An attractive hypothesis is that ATP may also be used as an extracellular messenger by maturing LSO neurons (Dietz et al., 2012; Babola et al., 2020).

There is considerable controversy over whether neurons use lactate—rather than glucose—to fuel oxidative phosphorylation (Pellerin and Magistretti, 2012; Nortley and Attwell, 2017; Li and Sheng, 2021). Lactate would be released from glial cells via the astrocyte-neuron lactate shuttle. Neuronal computation is energetically expensive, with most of the energy used to reverse ion entry that has generated synaptic potentials and APs (Harris et al., 2012; Meyer et al., 2022). LSO neurons generate APs at unusually high rates of several hundred Hertz (Brosel et al., 2018). We examined transcripts for key enzymes and obtained evidence that LSO neurons do not primarily use lactate as a carbon source for ATP generation. The expression of *Slc16A2*, which encodes the neuronal monocarboxylate transporter MCT2, was absent from our sample. In contrast, the expression of *Slc2a3*, which encodes the neuronal glucose transporter GLUT3, was high (gene #201, 955 TPM). Expression levels of other genes (neuronal *Ldha*|lactate dehydrogenase A, #5,658, 4 TPM; astrocytic *Glo1*|lactoylglutahione lyase, #1,521, 82 TPM; *Glo2* = *Hagh*|hydroxyacylglutathione hydrolase, #119, 1,225 TPM; astrocytic *Slc16A1*|MCT1, #6,252, 3 TPM) confirm our conclusion that lactate-fueled OxPhos is not the major ATP producer in LSO neurons. This does not mean that LSO neurons exclusively utilize glucose (Brosel et al., 2018), and developmental changes may also be considered (Lujan et al., 2021). Collectively, our results support the conclusion of an extraordinary ATP demand in LSO neurons and suggest glucose—rather than lactate—as the primary energy source.

### The two clusters cover ~90% of the LSO neurons

Our patch-seq LSO neurons clearly separated into two clusters, with 65% belonging to pLSOs and 35% to LOCs. Literature data suggest that pLSOs and LOCs together comprise ~90% of all LSO neurons (~70% + ~20%). In addition to pLSOs and LOCs, several other neuron types have been described morphologically. No categorization data are available for mice, but five types have been described in gerbils and cats and seven in rats (Helfert and Schwartz, 1987; Rietzel and Friauf, 1998). A prominent subtype are the LSO marginal cells, so named because of the location of the somata along the edges of the LSO (review: Friauf et al., 2019). Marginal cells appear to comprise only ~5% of the population, regardless of the species analyzed (Ollo and Schwartz, 1979; Helfert and Schwartz, 1986, 1987; Rietzel and Friauf, 1998). Small neurons tend to be underrepresented in patch-clamp recordings, and we did not specifically target small somata or for somata located in the marginal zones of the LSO. Nevertheless, we are confident that we did not miss any major cell type.

Based on our results, we also find it unlikely that pLSOs and LOCs form further subclusters, although we did find differences in membrane properties between pLSOs —Onset and Sustained neurons. Interestingly, Onset and Sustained neurons have been described in both vGLUT2-positive and vGLUT2-negative pLSOs (they are presumably excitatory and inhibitory, respectively; Haragopal and Winters, 2023). This complicates the identification of distinct expression profiles for excitatory and inhibitory pLSO subclusters. Nevertheless, activity-dependent maturation could manifest differences in the expression profile and result in different membrane properties between Onset and Sustained pLSOs.

### Super DEGs in pLSOs

*Kcnh7*|Kv11.3 was DEG#6 in pLSOs (Figure 9A). The gene encodes ERG3, a pore-forming subunit of ether-a-gogo-related channels. ERG channels have a predominantly somatic location, are most active after intense electrical activity, and effectively modulate neuronal excitability, thereby complementing Kv1 channels (Hardman and Forsythe, 2009). The channels are inward rectifiers with unusual gating kinetics. They open at relatively negative membrane potentials, thus affecting the depolarization phase of an AP, and they inactivate very rapidly. The highest conductance is generated during repolarization (Shepard et al., 2007; Bauer and Schwarz, 2018), when the fast recovery from inactivation takes place (Bauer and Schwarz, 2018). Several studies showed a role of ERG channels in the nervous system (Sacco et al., 2003; Niculescu et al., 2013). In ventral cochlear nucleus neurons, they play a role in setting AP threshold and AP frequency (Yildirim and Bal, 2018). In the mouse MNTB, where ERG1 and ERG3 subunits are predominant, ERG currents modulate Kv1 currents and limit AP generation around AP threshold voltages (Hardman and Forsythe, 2009).

*Kcna1*|Kv1.1 was DEG#92 in pLSOs (Figure 9A). Kv α subunits have received extensive attention in the auditory brainstem, and immunoreactivity has been demonstrated in several species in somata, dendrites, and axon terminals (mouse: Wang et al., 1994; Brew et al., 2003; rat: Barnes-Davies et al., 2004; gerbil: Nabel et al., 2019); three bat species: (Rosenberger et al., 2003; Pätz et al., 2022). Kv1.1 channels were most intensively analyzed. They carry a rapidly activated, low-threshold, and sustained K^+^ current (I_KL_) that determines the 1st AP latency as well as the AP waveform (Kole et al., 2007; Fischl et al., 2016). MNTB neurons in Kv1.1 knockout mice display a reduced I_KL_ and are hyperexcitable (Brew et al., 2003). In line with this, pharmacological blockade of Kv1.1 channels in pLSOs with α-dendrotoxin (NB: blocks also Kv1.2 and Kv1.6; Oak and Yi, 2014) convert a single-firing to a multiple-firing pattern (Barnes-Davies et al., 2004). Kv1.1 knockout mice display behavioral deficits in sound localization (Robbins and Tempel, 2012; Karcz et al., 2015), and their LSO neurons show a weaker capability of encoding interaural level differences (Karcz et al., 2011). pLSOs clearly differ from LOCs regarding Kv1.1 channels. The results provide further evidence for the importance of temporal integration of excitation and inhibition during sound localization, especially in the ascending branch of the LSO.

The Kvβ3-encoding gene *Kcnab3* was DEG#146 in pLSOs. Three Kvβ isoforms have been described in the brain (Heinemann et al., 1995; Rhodes et al., 1996). They have oxidoreductase activity and interact with the pore-forming α subunits of Kv1 and Kv4 channels (Dwenger et al., 2022). The assembly generally increases whole cell Kv current and modifies voltage sensitivity (Pongs and Schwarz, 2010). We are not aware of a study on Kvβ3 in the auditory system. Our results show high protein abundance in the major SOC nuclei and co-distribution of Kvβ3 and GlyT2 in axon terminals terminating on pLSO somata, most likely originating from MNTB neurons (Figure 12D).

*Slc17a6*|vGLUT2 was DEG#26 in pLSOs. Vesicular glutamate transporters are involved in the storage and release of glutamate. Our patch-seq results for genes expressing vGLUT confirm *in situ* hybridization results showing the absence of vGLUT1 and the presence of vGLUT2 in mouse LSO somata (Ito et al., 2011). They are also consistent with the detection of vGLUT2 in the LSO of rat, mouse, and owl monkey at the protein level (Blaesse et al., 2005; Haragopal and Winters, 2023) and at the mRNA level (Hackett et al., 2011). The association of vGLUT2 with pLSOs is consistent with findings in rats that vGLUT2-positive LSO neurons project into the contralateral inferior colliculus (IC; virtually no ipsilateral projection), thus representing ascending neurons (Ito and Oliver, 2010). Moreover, concerning the three classical fast neurotransmitters (glutamate, glycine, GABA; expressed by 44 pLSOs in different combinations), we show a surprisingly high 4:1 ratio between a purely excitatory and a purely inhibitory phenotype (64% vs. 16%; Figure 16; Supplementary Table S6). The remaining 20% show a mixed phenotype (predominantly glutamate plus GABA; notice rounding effect). Thus, the present results indicate a dominant glutamatergic phenotype in mouse pLSOs, whereas glycinergic and/or GABAergic neurons are much less abundant. A mixed phenotype (glutamate plus glycine) has also been described for the projections of LSO neurons into the IC of rats (Fredrich et al., 2009). The authors found that all ipsilaterally projecting neurons were glycinergic, with 60% of them also staining for glutamate. Similar to mice, ¾ of the LSO neurons in gerbils are excitatory (Mellott et al., 2022). In contrast, excitatory and inhibitory LSO neurons appear to be balanced in cats (Glendenning et al., 1992) and guinea pigs (Helfert et al., 1989). We propose that species differences underlie the different ratios.

*Gria4*|GluA4 was DEG#40 in pLSOs. In contrast, GluA1 subunits appear to be a marker for LOCs (DEG#68). Kinetics of glutamatergic ionotropic receptors can be in the submillisecond range (Geiger et al., 1997), and the fastest kinetics are obtained when GluA4 subunits are present (auditory brainstem: Geiger et al., 1995; Yang et al., 2011; Rubio et al., 2017). By contrast, GluA1-containing receptors are slow-gating. At calyx of Held synapses, GluA4 is the main determinant for the fast synaptic responses and indispensable for driving high-fidelity neurotransmission and conveying precise temporal information (Yang et al., 2011). GluA4 is also expressed in auditory nerve synapses (Rubio and Wenthold, 1997), and its loss impairs the acoustic startle response (García-Hernández and Rubio, 2021). These results emphasize the importance of this subunit in the CAS.

AMPAR-mediated EPSCs in the LSO are characterized by sub-millisecond decay times (0.77 ms) that are slowed upon acoustic trauma, which is mirrored by mRNA decreases for GluA4, yet increases for GluA1 (Pilati et al., 2016). In this line, our transcriptomic results imply different kinetics at glutamate receptors between pLSOs and LOCs. Indeed, our electrophysiological findings demonstrate considerably faster EPSC kinetics in pLSOs (Supplementary Figures S7D,E; Supplementary Table S5.1; Figure 16). The involvement of pLSOs in sound localization requires sub-millisecond precision in signal integration. According to our data, hyper-precise kinetics are not present in LOCs, which seem to protect the auditory nerve fibers from excitotoxic effects of acoustic overexposure, a process occurring in minutes (Accardi, 2014). Notably, pLSOs have myelinated axons, whereas LOC axons are unmyelinated (Brown, 2011).

The sixth Super DEG in pLSOs, *Gabrb3*, codes for GABAARβ3, the β3 subunit of GABA_A_ receptors (DEG#72). GABA_A_ receptors are heteropentamers, most often consisting of subunits from three different families in a 2:2:1 fashion (2α + 2β + 1 of either γ, δ, ε, π). The most common arrangement is 2α_1_2β_2_γ_2_. Intermediate levels of *Gabrb3* mRNA were identified in the LSO of rats and mice (Campos et al., 2001; Fischer et al., 2019), and immunosignals for β3 subunits have been described in the rat LSO (Pirker et al., 2000). Our patch-seq results confirm the expression in pLSOs. *Gabrb3* loss results in severe cochlear histopathology, providing evidence for a functional GABAergic innervation of the cochlea (Maison et al., 2006). The physiological role of β3 has not yet been analyzed in the CAS. The same holds for GABAARα1, whose encoding gene was a Super DEG in LOCs. In a mammalian expression system, receptors containing the α1 subunit responded to GABA application with long-duration openings that were organized in multi-opening bursts, in contrast to brief-duration, isolated openings of receptors containing α6 subunits (Fisher, 2004). The author suggested that GABA acts as a high efficacy agonist at the former receptor type. Consequently, LOCs may be more efficiently inhibited by GABA than pLSOs (see also Fischer et al., 2019). Interestingly, a given pLSO neuron or LOC neuron displays an almost inverse expression pattern for β3 or α1 GABAAR subunits (Figure 13A), indicating substantial architectural and functional differences.

### Super DEGs in LOCs

For LOCs, the Super DEGs *Calca* and *Ucn*, encoding the neuropeptides CGRP and urocortin, respectively, were DEG#1 and DEG#2 (Figure 4B). There is clear evidence that CGRP is a molecular marker for LOCs (mouse: Maison et al., 2003; Wu et al., 2018). Reportedly, CGRP is not associated with pLSOs, and our results confirm this, although we found few CGRP transcripts in 13% of these neurons (< 2 TPM; Figure 14A). Our results document the impressively complex multi-transmitter properties of LOCs at the single-cell level. The cohort displays 12 multi-transmitter variants, more than 90% (28/30) of the neurons express at least two of the seven transmitter genes analyzed, and up to five neurotransmitter genes can be co-expressed by a single neuron (Figure 14B). The dominant combination is GABA + CGRP + urocortin, which was observed in 61% of the LOCs (Figure 16; Supplementary Table S6). In 10 neurons, it was flanked by the expression of a fourth or even a fifth gene. Urocortin demonstrates an even higher cell-type specificity than CGRP, as gene expression was completely absent from pLSOs (Figure 14A). Taken together, our patch-seq results demonstrate high transmitter complexity for LOCs at single-cell resolution. They also imply functional diversity within this relatively small population of efferent neurons.

*Scn3a*|Nav1.3 (DEG#11 in LOCs) encodes an α subunit of Nav1.3 channels generally less common than Nav1.1, Nav1.2, and Nav1.6 channels in the central nervous system (Scott et al., 2010). Nav1.3 channels are more abundant in the fetal than the adult brain (Liao et al., 2023). Nav1.3 currents recover rapidly from inactivation, which results in low AP thresholds and high AP frequencies (Cummins et al., 2001; Liao et al., 2023). However, the distinct expression of *Scn3a*|Nav1.3 in LOCs does not explain the higher AP threshold compared to pLSOs (Supplementary Figure S6J). AMBA shows no *Scn3a* expression in the LSO at P56, and P14 data was not available (Supplementary Figure S5).

As gene expression for Nav1.3 subunits was low in pLSOs, we assessed other Nav genes and found the highest expression level for *Scn8a*|Nav1.6, followed by *Scn1a*|Nav1.1 (66 and 18 TPM; Supplementary Figure S8A; Supplementary Table S6). The LOC cohort displayed considerably lower Nav1.6 expression (12 TPM; < 5-fold). Nav1.6 subunits are abundant in the nervous system and occur in various excitatory and inhibitory neurons. They form dense clusters at axon initial segments and nodes of Ranvier where they are the dominant Nav subtype (Caldwell et al., 2000; Chen et al., 2008; Zybura et al., 2021). For example, they are the major isoform at Calyx of Held-MNTB axon terminals where the abundance increases during development (Leao et al., 2005). In the auditory nerve, Nav1.6 and Nav1.1 are placed at the AP generator site, together with the submembraneous scaffolding protein ankyrin (Kim and Rutherford, 2016). As demonstrated in several brain regions, Nav1.6 channels contribute to peak Na^+^ currents and repetitive AP firing (references summarized in Chen et al., 2008). They display persistent and resurgent Na^+^ currents, lower the AP threshold and mediate repetitive spiking (Zybura et al., 2021). Together, the above findings suggest an important role for Nav1.6 channels in initiation and propagation of high-frequency AP bursts in LSO neurons, particularly in myelinated, mature pLSOs.

Cacna1e, coding for Cav2.3, was DEG#26 in LOCs. Previously, we identified mRNA for Cav2.3 in LSO neurons (Jurkovicova-Tarabova et al., 2012). Cav2.3 is the α1 subunit of R-type Cav channels (Catterall, 2011). Together with PQ-and N-type channels (containing Cav2.1 and Cav2.2 subunits, respectively), R-type channels mediate presynaptic Ca^2+^ influx and neurotransmitter release (Ricoy and Frerking, 2014; Held et al., 2020). Among the 18 Cav genes expressed in the LSO cohort, *Cacna1e*|Cav2.3 demonstrated the highest value by far (mean TPMs: LOCs 1,491; pLSOs 182; Supplementary Figure S9A; Supplementary Table S6). High TPM values also occurred for Cav2.2 in both neuron types (pLSOs 414; LOCs 363). Together, we reason that LOCs likely prioritize Cav2.3 subunits over Cav2.2 subunits, whereas pLSOs favor the reverse order. On the other hand, *Cacna1a*|Cav2.1 expression seems be negligible in both groups (6.1 vs. 2.2 TPM). The functional impact of this needs to be determined.

### Cluster similarity

Our Cluster similarity scoring finally revealed 12 genes, four of which code for TRP channels, another four for glutamate receptor molecules, two for Cav channels and one each for glycine and GABA receptor subunits (Figure 15). Among the four TRP channels were two TRPM channels (melastatin) as well as one TRPML (mucolipin), and one TRPP (polycystin) channel (TRPM2, TRPM7, TRPML1, TRPP5). TRP channels are cation channels that generally cause cell depolarization. However, they can also be associated with intracellular organelles and function as intracellular Ca^2+^ release channels (Gees et al., 2010). They play a crucial role in sensory physiology, possibly contributing to hearing (Venkatachalam and Montell, 2007). TRP channels have been poorly studied in the CAS. In the mouse ventral cochlear nucleus, activation of TRPM2 channels shifts V_rest_ to more positive values (Bal et al., 2020). The authors reasoned that TRPM2 channels, which are induced by oxidative stress, may play a modulatory role in setting the excitability level of stellate cells. To our knowledge, there is no study on TRPM or TRPPP channels in the SOC. Our results show that pLSOs and LOCs are similar in their TRP channel composition, as also evidenced by the absence of any DEG in this group. These results pave the way for further investigations. Of note is the relatively low percentage of neurons expressing a given TRP gene (highest value: 50% for TRPM7 in LOCs), indicating heterogeneity within a given cluster. This issue of inter-cellular heterogeneity is of interest for future studies.

Regarding Cluster similarity for glutamate receptor molecules, ~15% (4/26; including 18 ionotropic and 8 metabotropic) met the criteria, namely GluA2, GluK5, GluN1, and mGluR8. This corresponds to one gene in each major class (AMPA; kainate, NMDA, metabotropic; Figures 15A2,B). Regardless of subtype, ~75% of the LSO neurons showed GluA2 transcripts, the highest proportion across all 32 ligand-gated ion channels (Figure 13A). The Cluster similarity observed for GluA2 contrasts with the Super DEGs GluA1 and GluA4. Edited GluA2 subunits render AMPA receptors Ca^2+^ impermeable and are abundant in the brain. AMPA receptors lacking GluA2 are Ca^2+^-permeable, allowing them to serve as signaling molecules via Ca^2+^ influx, in addition to mediating excitatory neurotransmission (Man, 2011).

Kainate receptor subunits, including GluK5, have been identified in the SOC by immunohistochemistry and pharmacological analysis (Petralia et al., 1994; Löhrke and Friauf, 2002; Vitten et al., 2004). Notably, GluK5 subunits have high agonist affinity and fast kinetics (Dhingra et al., 2022). In summary, there is consistent evidence that both types of LSO neurons are equipped with fast gating glutamate receptors, and pLSOs even more so than LOCs.

Our results on GluN1 confirm immunohistochemical findings in the LSO (Nakagawa et al., 2000) and attribute the subunit to both pLSOs and LOCs. Glycine, a co-agonist at NMDA receptors, binds to GluN1 subunits, whereas glutamate binds to GluN2. Consequently, NMDA receptors require both subunits to be functional. Our study shows similar expression for both isoforms (GluN2B and GluN2D), and the majority of pLSOs express GluN2B, especially the Sustained neurons (Figure 13A). Our GluN2B results are consistent with a pharmacological report suggesting a correspondence of the NMDA receptor subunit with the period of major circuit refinement at MNTB-LSO synapses (Case and Gillespie, 2011). In particular, GluN2B subunits confer relatively long decay kinetics to NMDA receptors, and the developmental shortening of EPSCs likely results from a subunit substitution of GluN2A for GluN2B.

For genes involved in inhibitory neurotransmission, Cluster similarity was demonstrated for the ionotropic GlyR subunit GlyRβ and the metabotropic GABABR1 (Figures 13A2,B2, 14A; Supplementary Figure S12A). For GlyRβ, we found expression in 79% of the pLSOs and 67% of the LOCs. The 79% value was the highest observed in the pLSO cohort. The presence of GlyRβ transcripts is expected, as this subunit is a mandatory component of heteropentameric GlyRs and essential for their postsynaptic localization (Weltzien et al., 2012). The higher TPM values for the adult GlyRα1 compared to the fetal GlyRα2 (Figure 13A) indicate that most of the replacement of the fetal by the adult α subunits (Dutertre et al., 2012) has occurred by the time mice begin to hear, at least in pLSOs (α3 and α4 transcripts were not detected in our sample).

GABABR1 is one of two GPCR molecules activated by GABA (review: Bassetti, 2022). The level for GABABR1 transcripts was moderate in our LSO sample (~25 TPM), >10-fold higher than the very low *Gabbr2*|GABABR2 expression (~1 TPM; Supplementary Figure S12A; Supplementary Table S6). Since GABABRs are obligatory heterodimers composed of GABABR1 and GABABR2 subunits (review: Frangaj and Fan, 2018), the difference in expression is surprising. Based on our transcript results, we cannot localize the GABABR subunits to presynaptic or postsynaptic sites. We have recently shown that postsynaptic GABABRs mediate hyperpolarization in a modulatory manner in mouse pLSOs (Fischer et al., 2019). GABABR signaling has previously been shown to mediate long-lasting depression at inhibitory gerbil MNTB-LSO synapses (Kotak et al., 2001). This form of synaptic depression declines with age and may contribute to the use-dependent reorganization of MNTB axon arbors.

In the Cav channel group (Zamponi et al., 2015), we observed Cluster similarity for two subunits, Cav1.3 and Cavγ2. Cav1.3 plays an important role in the auditory system. It is involved in deafness (Platzer et al., 2000) and in the development of auditory brainstem structures, including the LSO (Hirtz et al., 2011, 2012). Functional Cav1.3 channels have been described in pLSOs (Jurkovicova-Tarabova et al., 2012). Therefore, the present results at the mRNA level are consistent with previous findings at the protein level.

Cavγ2, also known as stargazin or TARPγ2 (most literature refers to stargazin), is one of eight Cavγ isoforms in a functionally diverse family (Chen et al., 2007; Bissen et al., 2019). Six of the eight Cavγ isoforms belong to the TARP subfamily, including Cavγ2 (and Cavγ5). TARP stands for “Transmembrane AMPAR regulatory protein,” implying an interaction with AMPA receptors and not a mandatory role as a true Cav subunit. TARP/AMPAR complexes promote the correct trafficking of AMPARs and their targeting to the synaptic surface (review: Jacobi and von Engelhardt, 2017). TARPγ2-containing AMPARs have been localized in presynaptic axon terminals where they modulate GABA release (Rigby et al., 2015). The expression profile of Cavγ2 (and the other AMPAR regulatory subunits) and the functional characterization in the auditory brainstem await future studies. In this context, it is worth noting that *Cacng5*|Cavγ5 was DEG#12 in pLSOs.

*Kcnq2*|Kv7.2 was not on the top 12 list of genes showing Cluster similarity (its position was 18; Figure 15B2). Nevertheless, we analyzed Kv7.2 in combination with Kv7.3 by means of immunohistochemistry and pharmacology. Kv7.2 and Kv7.3 are primarily found in the brain (Baculis et al., 2020). They are the major molecular correlate of low-threshold, non-inactivating Kv channels that mediate the M current, which is hyperpolarizing and critical for preventing neuronal hyperactivity (Wang et al., 1998; Robbins, 2001; Brown and Passmore, 2009). In the calyx of Held terminals, Kv7 channels, most likely Kv7.5 homomers, control release probability through changes in V_rest_ (Huang and Trussell, 2011). In adition, they function to facilitate reliable high-frequency synaptic signaling (Zhang et al., 2022). Our results suggest a similar function for Kv7.2 and Kv7.3 channels in pLSOs. Whether the channels are localized in pLSO axon terminals and involved in modulating transmitter release requires further analysis.

### Species differences

There is increasing evidence that a variety of characteristics vary considerably among species in the SOC, including the LSO. Species-specific effects (e.g., low-frequency vs. high-frequency hearing) on structure and function are well known (Heffner and Heffner, 1992; Grothe, 2000; Fritzsch et al., 2013) and are evident in the SOC. First, there is considerable variation in the arrangement, shape, and relative size of individual SOC nuclei (Friauf et al., 2019). Second, there are species differences in the location of olivocochlear neurons in the SOC (Aschoff and Ostwald, 1987; Brown, 2011). Third, there are species differences in the quantitative contribution of LOCs and MOCs to olivocochlear fibers (Perrot and Collet, 2014). Fourth, interspecies differences may explain the inconsistencies between reports on the transmitter repertoire of LOCs (Guinan, 2011). Fifth, discrepancies in the excitatory/inhibitory transmitter ratio have been reported for pLSO neurons (Williams et al., 2022). In the heterogeneous LSO, these differences have created multi-scale, long-standing, and persistent puzzles (Guinan, 2011; Williams et al., 2022). Consequently, general statements about the LSO when pooled from different species must be interpreted with caution. We have taken this into account for the summary cartoon (Figure 16), which shows results from mice only.

### Validating the patch-seq results

For a few genes, we exemplarily validated our transcriptomic results on the protein level (Figures 5, 10, 11, 12). Furthermore, we compared them with those of a very recent study using single-nucleus sequencing of LOCs (Frank et al., 2023). The comparison revealed considerable similarity between the genes detected, thus strengthening each study. For example, both studies found expression of Col4a3 and B3galt1 in LOC neurons.

### Methodological considerations

We do not want to hide the fact that the patch-seq method has its limitations. The general limitations of scRNA-seq also apply to the patch-seq variant. Contamination from the extracellular solution can lead to false positives and affect the quality of the results. To minimize this problem, we performed stringent quality controls (see Methods). The low throughput (we patched 263 neurons but collected only 103) reflects our caution in cell harvesting to minimize contamination. In addition, we took advantage of the fact that extracting the cytosol and the nucleus significantly increases the RNA yield and improves the quality of the transcriptomic data. Finally, by including negative controls and carefully monitoring the harvesting process, we were able to gain insight into potential sources of contamination. Our observations indicate that the effects of contaminants on the transcriptomic profiles were generally small. For example, ChAT expression and urocortin expression were absent in the pLSO cohort. Nevertheless, we attribute some of the results on CGRP (in 12.5% of pLSOs) and HCN (in 17% of LOCs) to contamination (see Figure 14; Supplementary Figure S11). To allow a *post hoc* reaction to the effects of the contamination problem, we have designed the gene tables in such a way that the readers can interactively change the TPM thresholds and thus adjust the stringency of the filtering criteria to their personal needs (*cf.* Supplementary Table S6).

The age of the animals is another factor influencing contamination. In patch-seq experiments in adult tissue, where a dense perineuronal network of glial cells is present, the risk of contamination is higher. This was one of the reasons why we used young mice (P10-P12) in the present study. By limiting the sampling to this age, we also obtained a unique database for the cell identity of the developing LSO neurons. Given that numerous electrophysiological, biochemical and anatomical studies have used and continue to use this age in the LSO, we believe that our database will be of great value to the research community.

Other challenges arise from the fact that, due to biological and technical limitations, only a fraction of mRNA is captured, leading to zero inflation and false-negative results. We believe that some of our results, such as not detecting HCN in all pLSOs, are affected by zero inflation.

Comparisons of results at the transcript level with those at the protein level are confronted with an imperfect correlation between the transcriptome and the corresponding proteome (Nie et al., 2007; Schwanhäusser et al., 2011; Liu et al., 2016). The mapping between high-throughput measurements at the level of the transcriptome and the corresponding proteome is complex (Gunawardana et al., 2015). Conclusions based on the transcriptome alone should therefore be treated with caution, and gene expression levels should only be used as a proxy for protein levels (Liu et al., 2016).

We assume that some of our unexpected results have biological causes, in addition to the artifacts mentioned above. For example, the lack of ChAT expression that we observed in most LOCs is consistent with the low *in situ* hybridization signals detected in AMBA LSO P14 sections. However, neurons in the facial nucleus and other motor nuclei are strongly labeled in the AMBA. It is possible that gene expression for ChAT is low in the LSO while protein abundance is high. Taken together, it is advisable for future studies to first extend the transcriptomic findings to the protein level in order to determine whether the described mRNA level is manifested in protein abundance and to localize the proteins in the neurons before starting functional analyses.

## Conclusion and outlook

Our study provides a comprehensive catalog of marker genes for the two major neuron types in the juvenile LSO. It also provides fundamental and comprehensive insights into the molecular composition of the ascending and descending microcircuits associated with the LSO and how this may relate to their function, including cell type-specific maturation. Our study suggests several areas for further investigation. It is also worthwhile to use our extensive data sets in a correlative manner. For example, it would be worthwhile to analyze which gene or gene network affects which physiological parameter (e.g., Kv genes and AP properties). For this purpose, we have added several supplementary files with data for each neuron in our sample (Supplementary Table S3, S6). In addition, the regulatory subunits of ion channels should be analyzed more thoroughly (Yan and Tomita, 2012). Due to space limitations, this was not done in the present study. In particular, the abundance of proteins interacting with AMPARs (Jackson and Nicoll, 2011; Cull-Candy and Farrant, 2021; Jacobi and von Engelhardt, 2021), kainate receptors (Palacios-Filardo et al., 2016), GABARs (Nakamura et al., 2016; Yamasaki et al., 2017), and GlyRs (Liu and Wong-Riley, 2013) deserves special attention. Our multidimensional single-cell expression data provide a valuable foundation for research in this direction. Regulatory subunits contribute to the diversity and function of these macromolecular signaling complexes. This is also true for the voltage-gated ion channels (Figure 9; Supplementary Figures S8–S11). It is very likely that SOC neurons, with their temporally precise, robust, and resilient neurotransmission behavior, contain highly specialized signaling molecules that differ at the single-cell level.

## Data availability statement

The data is available on NCBI GEO under the accession number GSE241761.

## Ethics statement

The animal study was approved by the German Animal Protection Law (TSchG §4/3). The study was conducted in accordance with the local legislation and institutional requirements.

## Author contributions

AM-S: Writing – review & editing, Writing – original draft, Visualization, Validation, Investigation, Formal analysis, Conceptualization. EP: Writing – review & editing, Visualization, Validation, Investigation, Formal analysis, Conceptualization. JF: Writing – review & editing, Visualization, Software, Formal analysis. KK: Writing – review & editing, Investigation, Conceptualization. TR: Writing – review & editing, Visualization, Investigation, Formal analysis. EF: Writing – original draft, Supervision, Resources, Project administration, Funding acquisition, Conceptualization, Writing – review & editing.

## Footnotes

^1^ https://www.bioinformatics.babraham.ac.uk/projects/fastqc/

^2^ http://www.bioinformatics.babraham.ac.uk/projects/trim_galore/

^3^ http://broadinstitute.github.io/picard/

^4^ https://mouse.brain-map.org/

^5^ http://www.genenames.org

## Glossary

AMBA: Allen Mouse Brain Atlas
AP: action potential
AUROC: Area Under the Receiver-Operating Characteristic
CAS: central auditory system
C_m_: membrane capacitance
CNIC: central nucleus of the inferior colliculus
CNS: central nervous system
DAB: 3,3′-Diaminobenzidine
τ_decay_: decay time constant
DEG: differentially expressed gene
DNLL: dorsal nucleus of the lateral lemniscus
FC: fold-change
FDR: false discovery rate
GSEA: Gene Set Enrichment Analysis
GO: Gene Ontology
HVG: highly variable genes
IC: inferior colliculus
INLL: intermediate nucleus of the lateral lemniscus
IQR: interquartile range
IUPHAR/BPS GTP: International Union of Basic and Clinical Pharmacology/British Pharmacological Society Guide to Pharmacology
KEGG: Kyoto Encyclopedia of Genes and Genomes
LOC: lateral olivocochlear neuron
LSO: lateral superior olive
MOC: medial olivocochlear neuron
MNTB: medial nucleus of the trapezoid body
OI: overlapping index
OxPhos: oxidative phosphorylation
P: postnatal day
PBS: phosphate-buffered saline
PCA: principal component analysis
PFA: paraformaldehyde
pLSO: LSO principal neuron
R_in_: input resistance
R_s_: series resistance
RT: room temperature
scRNA-seq: single cell RNA-sequencing
sEPSC: spontaneous excitatory postsynaptic current
sIPSC: spontaneous inhibitory postsynaptic current
SOC: superior olivary complex
SPON: superior paraolivary nucleus
τ_m_: membrane time constant
TPM: transcripts per million
tSNE: t-distributed stochastic neighbor embedding
V_hold_: holding potential
V_rest_: resting membrane potential
