# Supplementary material for: Molecular and functional profiling of cell diversity and identity in the lateral superior olive, an auditory brainstem center with ascending and descending projections
Source: Front Cell Neurosci. 2024 May 23;18:1354520. doi: 10.3389/fncel.2024.1354520 (PMC11153811; doi:10.3389/fncel.2024.1354520)
Supplement: Supplementary file 8 [file Data_Sheet_1.pdf]

Supplementary Figures

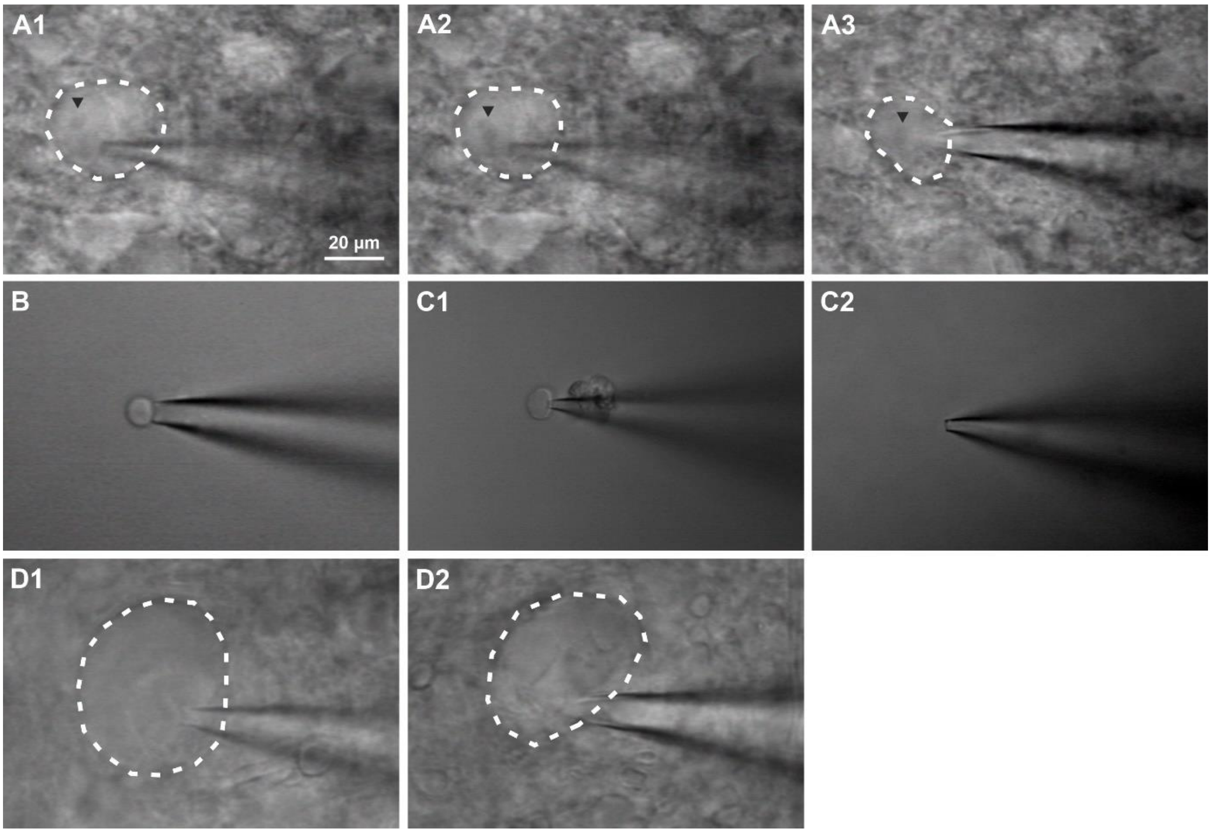

Supplementary Figure S1

Cytoplasm harvesting, and contamination control. **(A)** After completion of electrophysiological recordings, total RNA was extracted into the patch pipette by aspirating the neuron's cytoplasm via gentle suction. Shrinkage of the soma and entering of the nucleus into the pipette were monitored visually. Dashed lines depict outline of the soma. Black arrowheads mark the nucleus moving towards the pipette opening. **(B)** Pipette after cytoplasm harvesting showing no contamination (debris). A membrane patch closes the pipette tip, thus preventing extracellular material from entering the pipette. **(C)** Pipettes with debris **(C1)** and an open tip **(C2)**. **(D)** Two examples of swollen somata from recordings lasting > 15 min. Dashed lines depict outline of the soma.

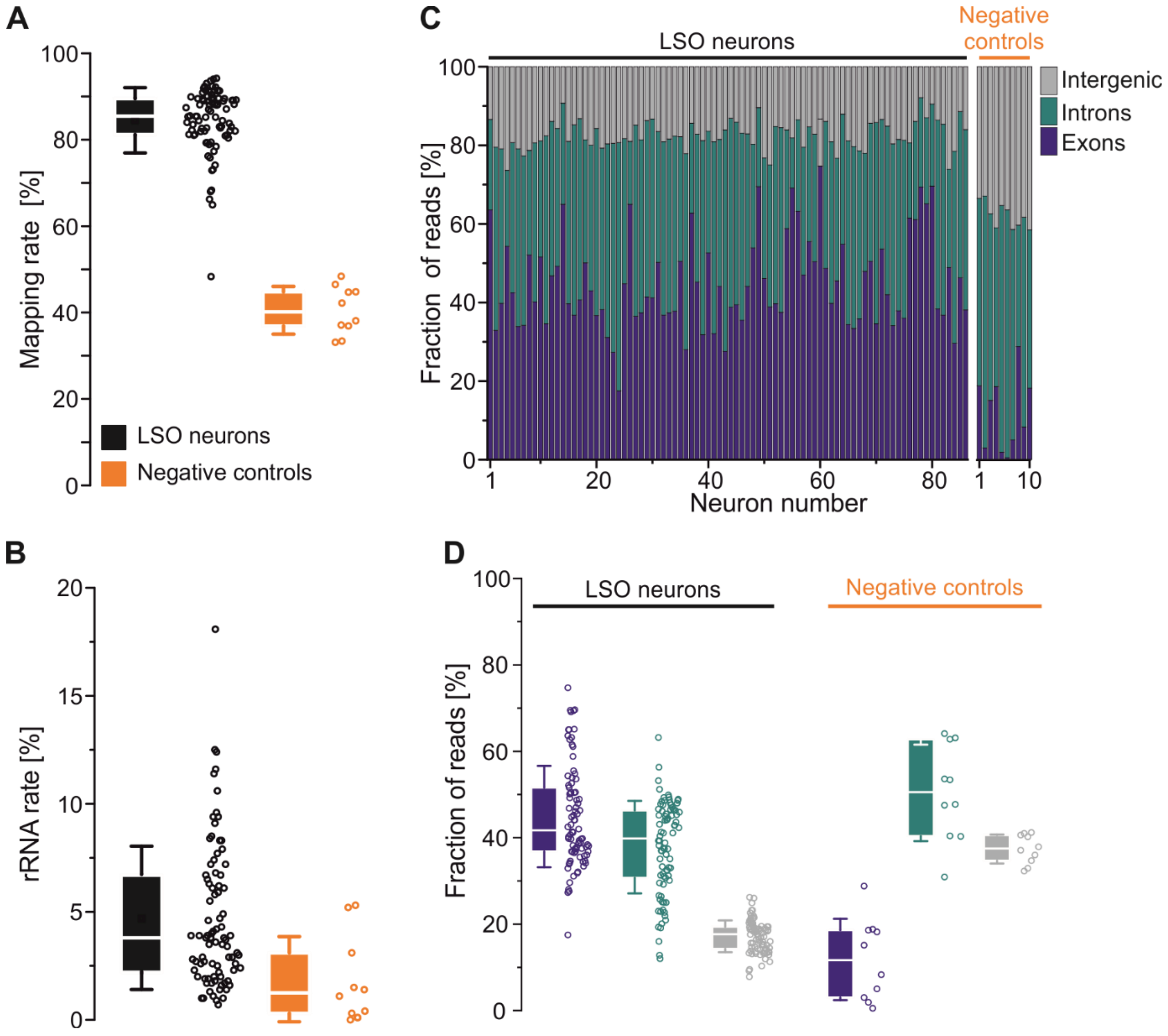

Supplementary Figure S2

Quality analysis of patch-seq data. **(A, B)** Mapping rate **(A)** and rRNA rate **(B)** for LSO neurons passing control criteria (black) and negative control (orange). **(C, D)** Fraction of reads mapped on intergenic parts of the genome (gray), introns (green), and exons (blue) for LSO neurons and for negative controls.

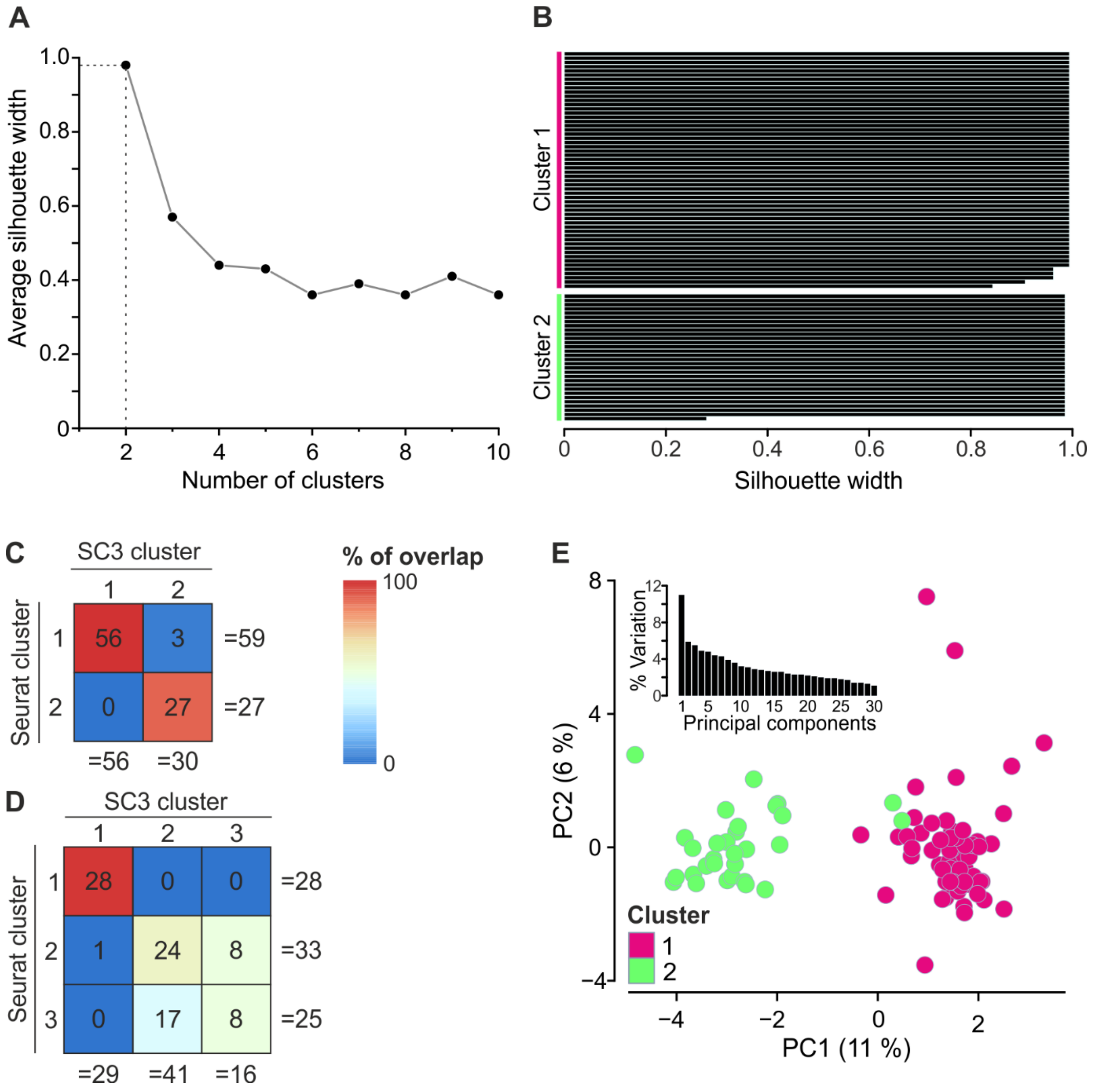

Supplementary Figure S3

Clustering results obtained by SC3 and Seurat. **(A)** Average silhouette widths obtained by unsupervised clustering (number of clusters: 2-10). **(B)** Silhouette widths of single neurons obtained for two clusters. **(C, D)** Comparison of clustering results computed using SC3 vs Seurat for two clusters **(C)** and three clusters **(D)**. Numbers of overlapping neurons are depicted in the matrix. **(E)** Principal component analysis (PCA) based on highly variable genes (HVG). Inset: Percentage of variation explained by each PC (1-30).

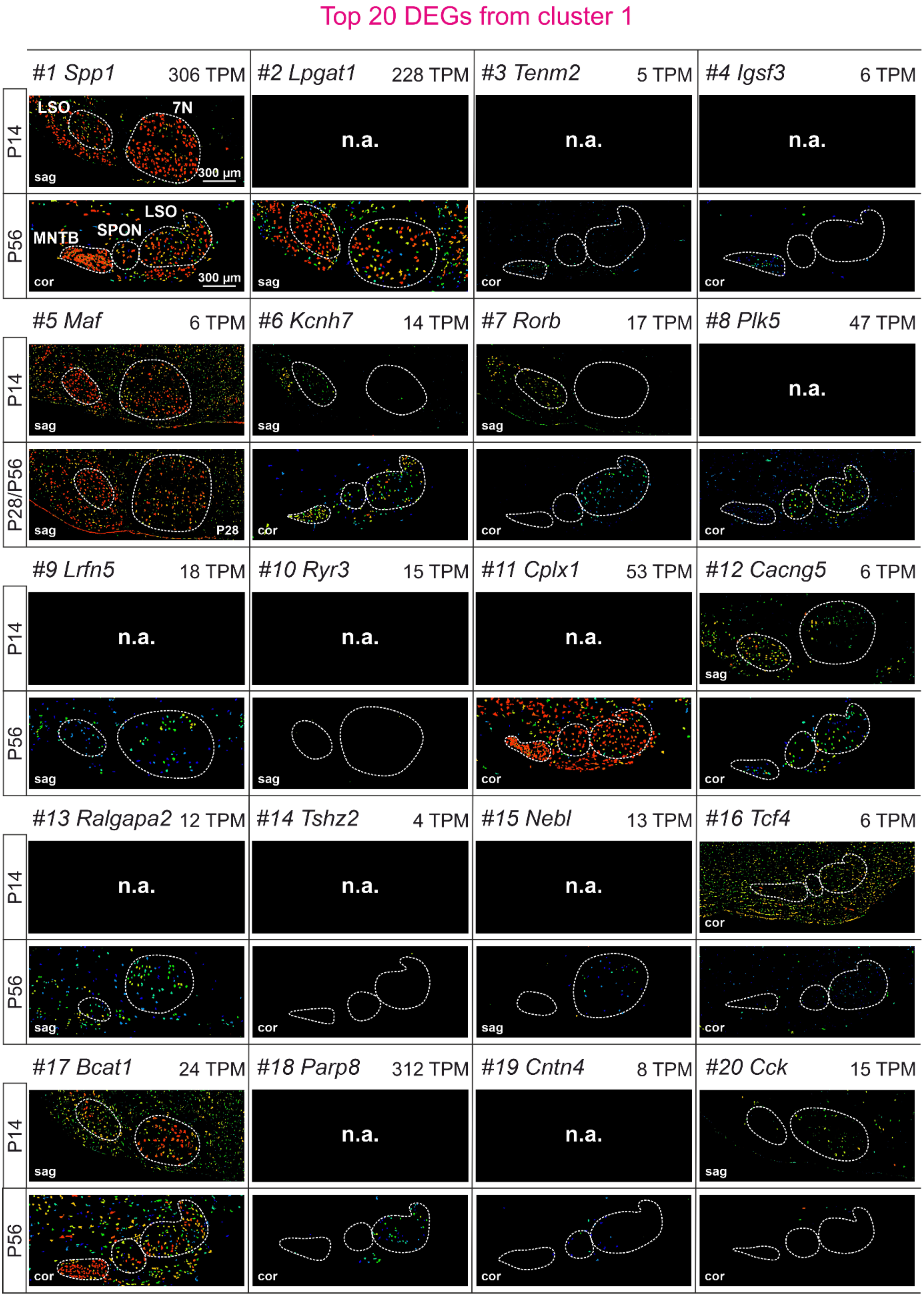

Supplementary Figure S4

Expression pattern of the top 20 DEGs from cluster 1 at P14 and at P28 or P56. *In situ* hybridization images of the SOC for the top 20 DEGs in cluster 1 (modified images from

AMBA, see Supplementary Table S7 for URLs). Cells with the highest probability of gene expression are highlighted in red, those with the lowest probability in blue. The TPM values from our patch-seq analysis at P10-12 are provided in the top right corner. Images depict coronal sections or sagittal sections. Notice that 19 panels are available at P56, one at P28 (#5 *Maf*), whereas only eight panels are available at P14. Abbreviations: cor, coronal; sag, sagittal; n.a., not available.

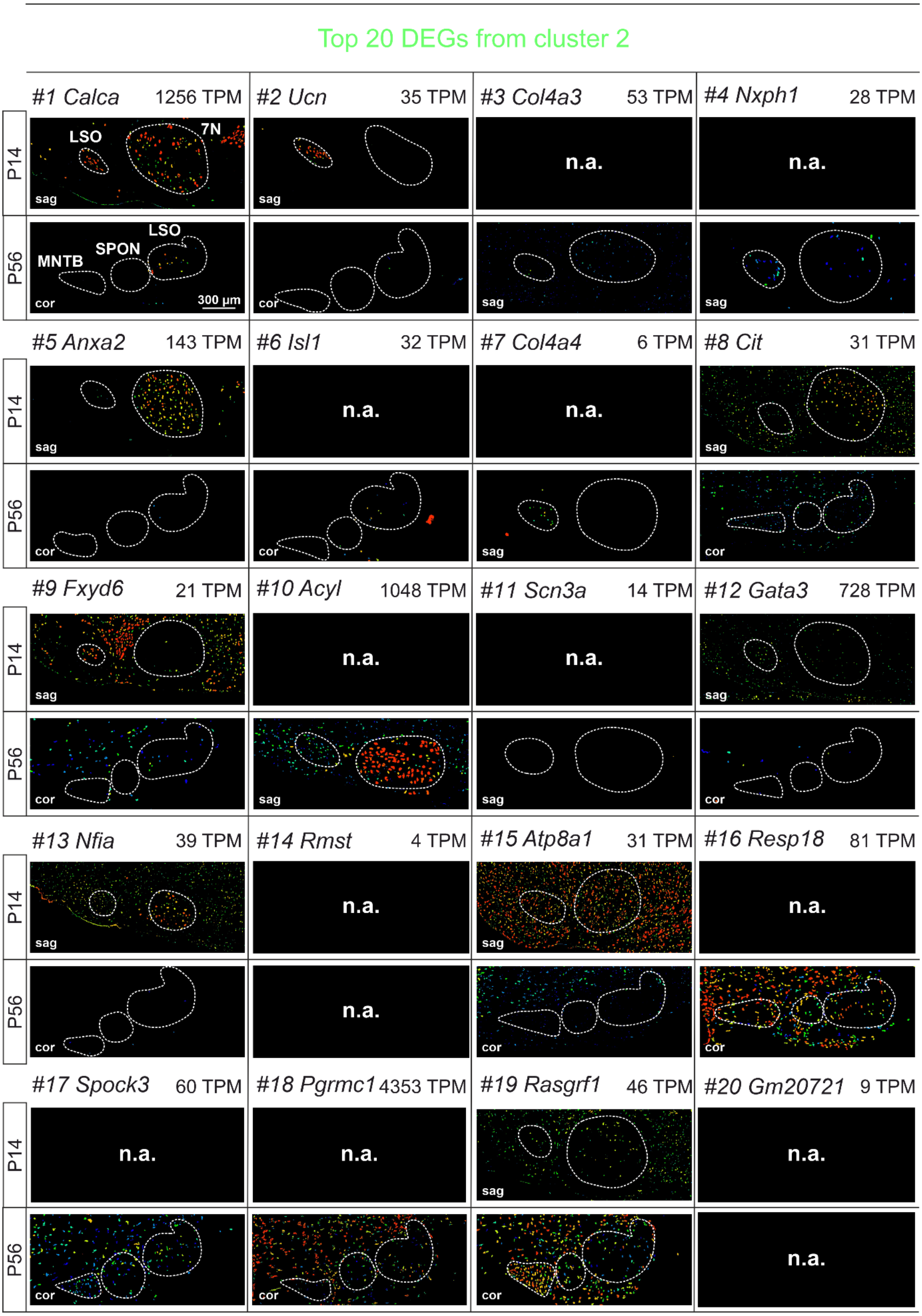

Supplementary Figure S5

Expression pattern of the top 20 DEGs from cluster 2 at P14 and P56. *In situ* hybridization images of the SOC for the top 20 DEGs in cluster 2 (modified images from AMBA, see

Supplementary Table S7 for URLs). Cells with the highest probability of gene expression are highlighted in red, those with the lowest probability in blue. The TPM values from our patch-seq analysis at P10-12 are provided in the top right corner. Images depict coronal sections or sagittal sections. Notice that 18 panels are available at P56, whereas only nine panels are available at P14. Abbreviations: cor, coronal; sag, sagittal; n.a., not available.

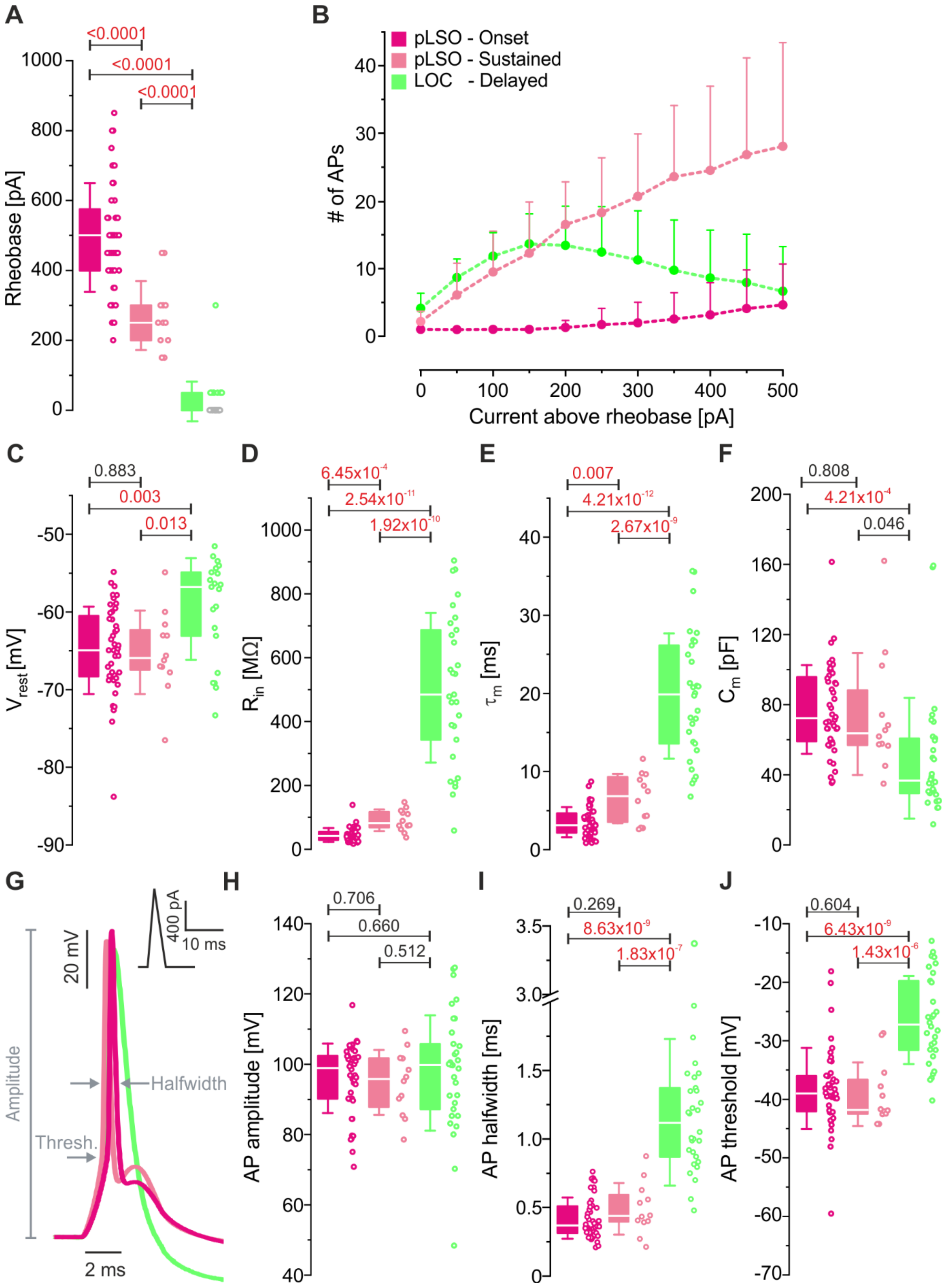

Supplementary Figure S6

Passive and active membrane properties of LSO neurons. **(A)** Statistics of rheobase. Open gray dots depict spontaneously active neurons. **(B)** Mean number of APs as a function of the injected current amplitude above rheobase. Bars depict SD. **(C-F, H-J),** Statistics of V_{rest}, R_{in}, τ_m, C_m, AP amplitude, AP halfwidth, and AP threshold. Open dots depict single neurons. Numbers

above bars represent p-values (in red when significant). **(G)** Representative APs, and relative evaluated parameters, of a pLSO - Onset (magenta), pLSO - Sustained (pink), and LOC - Delayed neuron (green), elicited via a triangular current pulse (inset, 5-ms, 1-nA). See also Supplementary Table S5.

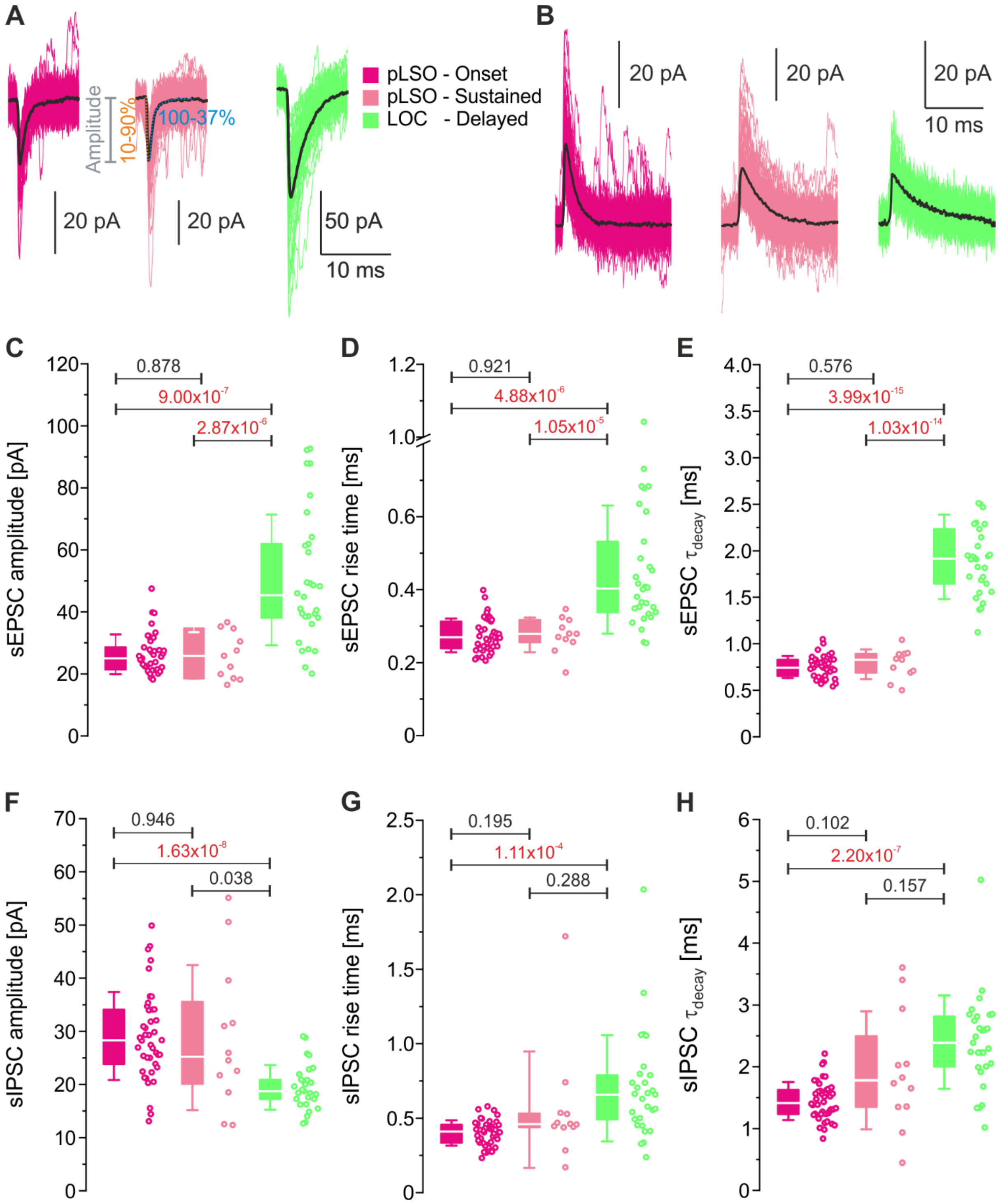

Supplementary Figure S7

Inhibitory and excitatory postsynaptic currents of LSO neurons. **(A, B)** Representative sEPSCs **(A)** and sIPSCs **(B)** of an pLSO - Onset (magenta), pLSO - Sustained (pink), and LOC - Delayed neuron (green). Colored traces are overlays of 100 sEPSCs or sIPSCs and black traces represent the graphical mean. sPSCs evaluated parameters are also shown. **(C-E)** Amplitude (C), rise time (D), and decay time constant (τdecay, E) of sEPSCs. **(F-H)** Amplitude (F), rise time (G) and τdecay (H) of sIPSCs. Open dots depict single neurons. Numbers above bars represent p-values (in red when significant). See also Supplementary Table S5.

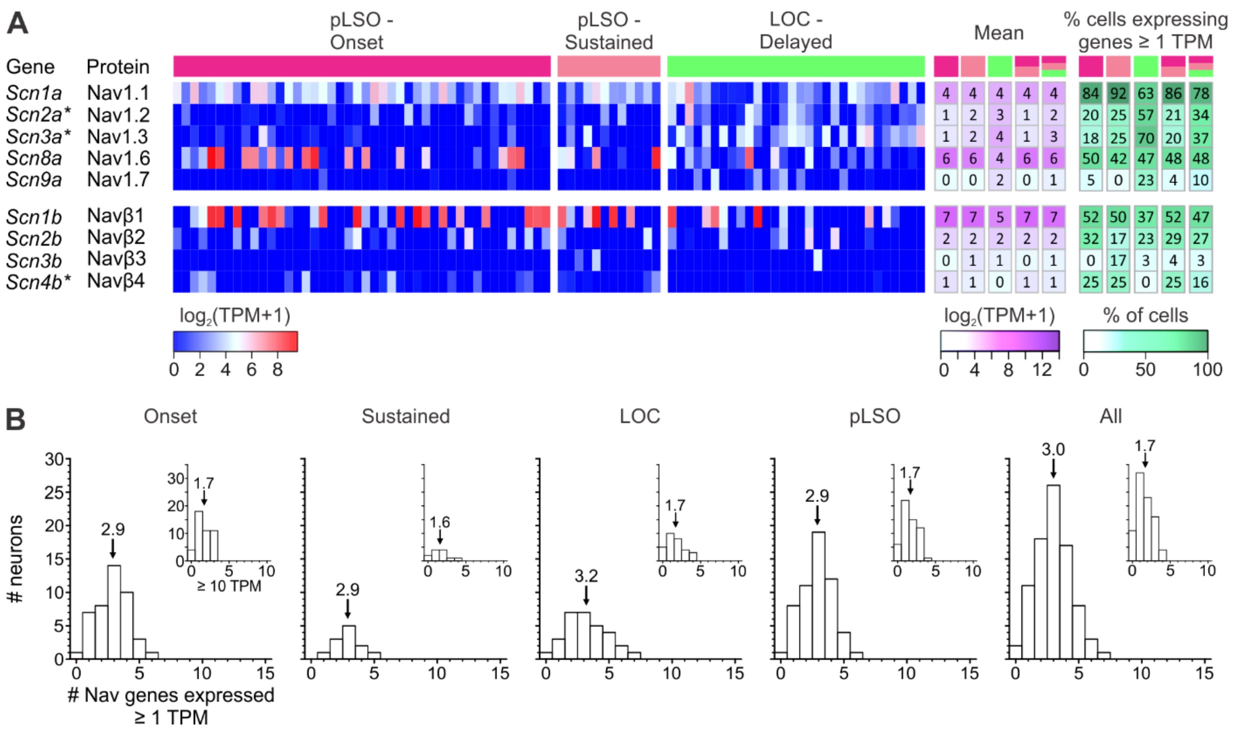

Supplementary Figure S8

Expression of genes encoding voltage gated sodium (Nav) α subunits and regulatory subunits (n = 5, 4). **(A)** Heat maps show expression levels [$\log_2$(TPM+1)] for single neurons (three cohorts). To the right, the mean and the percentage of cells expressing a given gene are depicted for five cohorts (pLSO - Onset, pLSO - Sustained, LOC - Delayed, pLSO, all neurons; n = 44, 12, 30, 56, 86). DEGs are marked with asterisks. **(B)** Frequency histograms showing distribution of gene expression for five cohorts (criteria: ≥ 1 TPM; ≥ 10 TPM for insets). Mean values are marked by arrows. See also Supplementary Table S6.

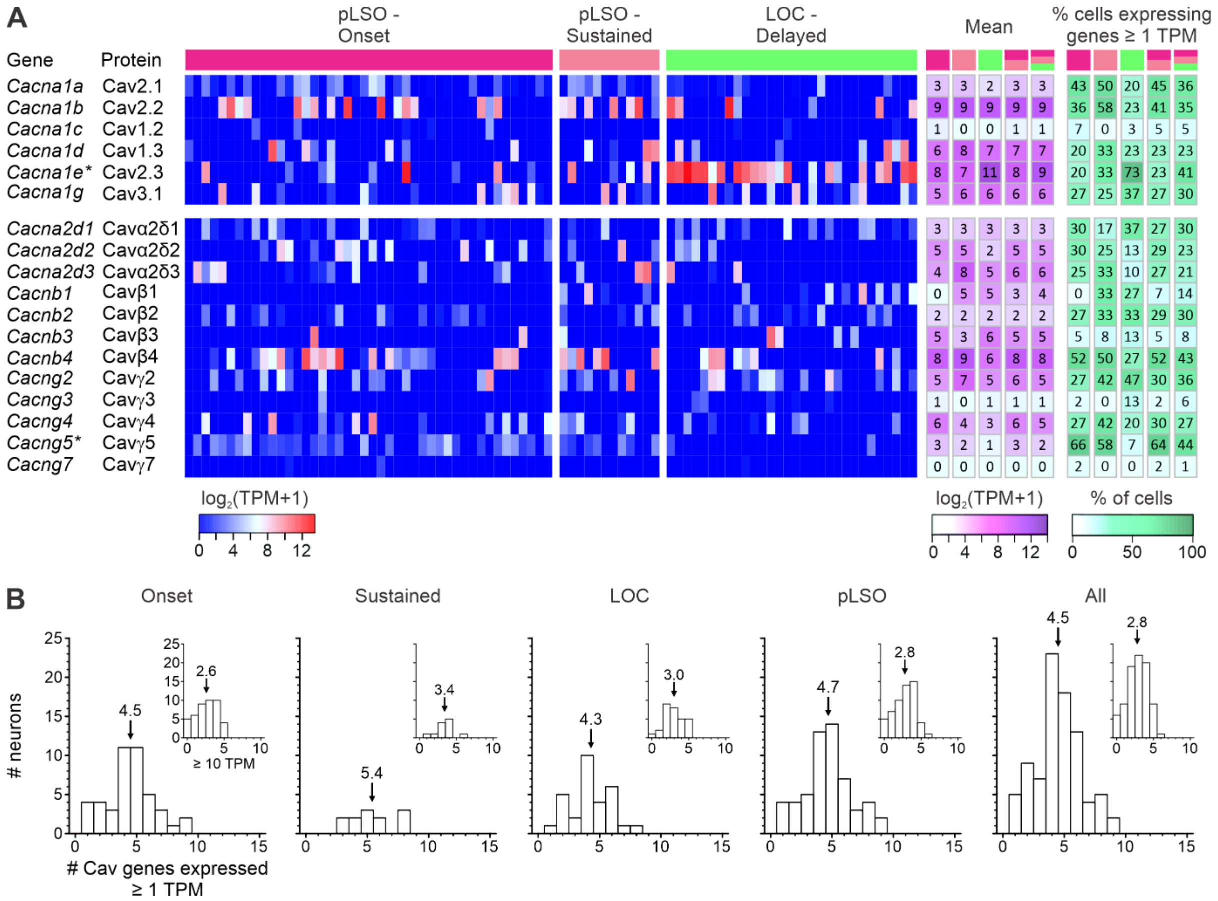

Supplementary Figure S9

Expression of genes encoding voltage-gated calcium (Cav) α subunits and regulatory subunits (n = 6, 12). **(A)** Heat maps show expression levels [$\log_2$(TPM+1)] for single neurons (three cohorts). To the right, the mean and the percentage of cells expressing a given gene are depicted for five cohorts (pLSO - Onset, pLSO - Sustained, LOC - Delayed, pLSO, all neurons; n = 44, 12, 30, 56, 86). DEGs are marked with asterisks. **(B)** Frequency histograms showing distribution of gene expression for five cohorts (criteria: ≥ 1 TPM; ≥ 10 TPM for insets). Mean values are marked by arrows. See also Supplementary Table S6.

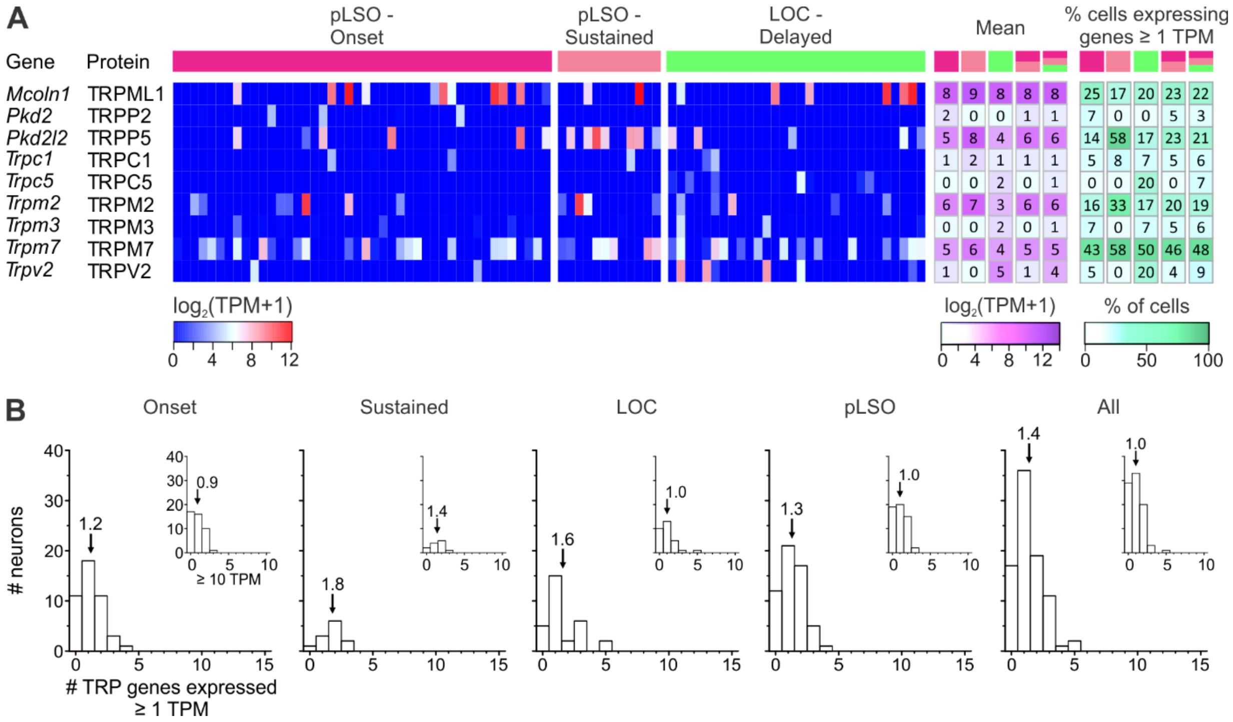

Supplementary Figure S10

Expression of genes encoding transient receptor potential (TRP) channels (n = 9). **(A)** Heat maps show expression levels [log$_2$(TPM+1)] for single neurons (three cohorts). To the right, the mean and the percentage of cells expressing a given gene are depicted for five cohorts (pLSO - Onset, pLSO - Sustained, LOC - Delayed, pLSO, all neurons; n = 44, 12, 30, 56, 86). **(B)** Frequency histograms showing distribution of gene expression for five cohorts (criteria: ≥ 1 TPM; ≥ 10 TPM for insets). Mean values are marked by arrows. See also Supplementary Table S6.

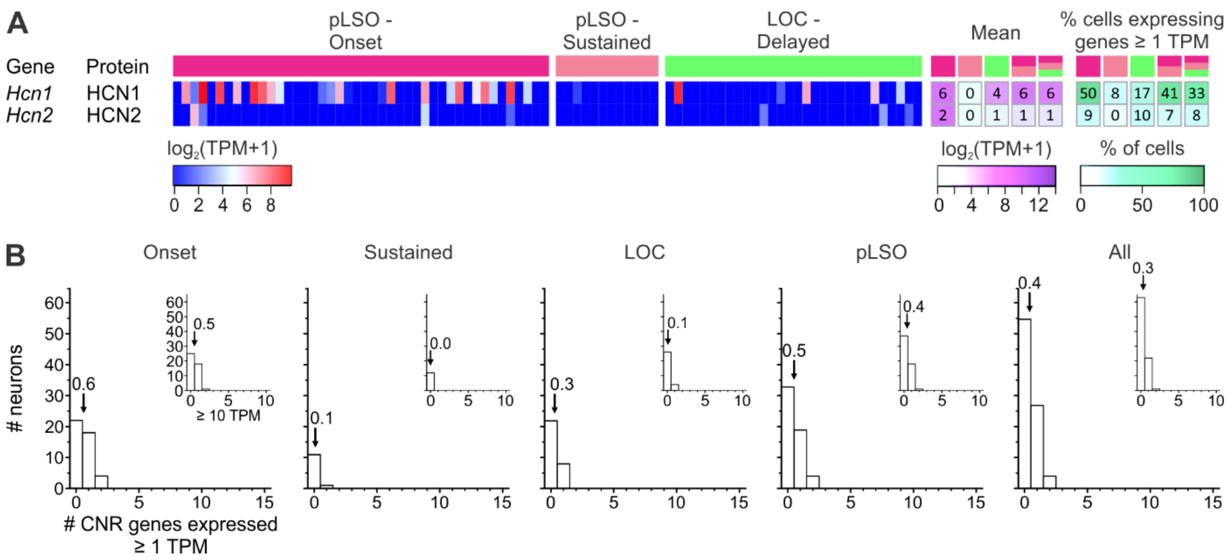

Supplementary Figure S11

Expression of genes encoding hyperpolarization-activated cyclic nucleotide-gated (HCN) channels (n = 2). **(A)** Heat maps show expression levels [log_2(TPM+1)] for single neurons (three cohorts). To the right, the mean and the percentage of cells expressing a given gene are depicted for five cohorts (pLSO - Onset, pLSO - Sustained, LOC - Delayed, pLSO, all neurons; n = 44, 12, 30, 56, 86). **(B)** Frequency histograms showing distribution of gene expression of five cohorts (criteria: ≥ 1 TPM; ≥ 10 TPM for insets). Mean values are marked by arrows. See also Supplementary Table S6.

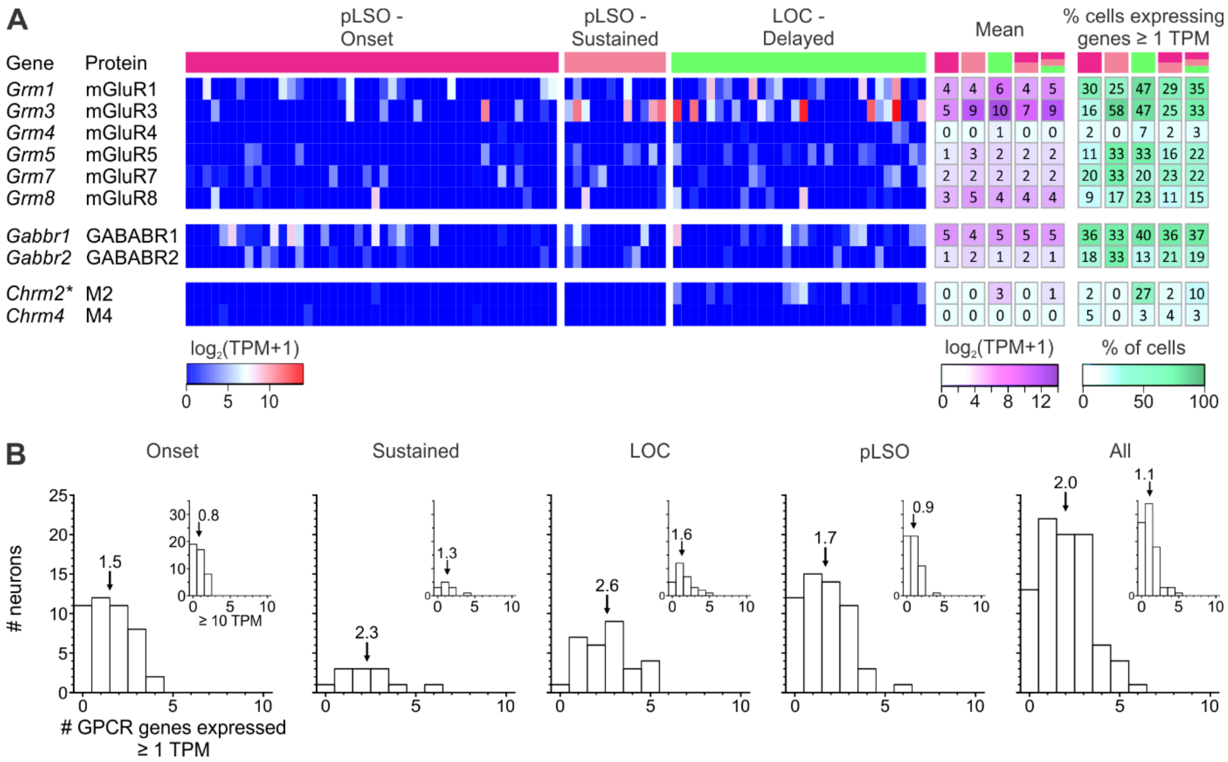

Supplementary Figure S12

Expression of genes encoding G protein-coupled receptors (n = 10). **(A)** Heat maps show expression levels [$\log_2$(TPM+1)] for single neurons (three cohorts). To the right, the mean and the percentage of cells expressing a given gene are depicted for five cohorts (pLSO - Onset, pLSO - Sustained, LOC - Delayed, pLSO, all neurons; n = 44, 12, 30, 56, 86). DEGs are marked with asterisks. **(B)** Frequency histograms showing distribution of gene expression of five cohorts (criteria: ≥ 1 TPM; ≥ 10 TPM for insets). Mean values are marked by arrows. See also Supplementary Table S6.
