# Supplementary material for: Molecular and functional profiling of cell diversity and identity in the lateral superior olive, an auditory brainstem center with ascending and descending projections
Source: Front Cell Neurosci. 2024 May 23;18:1354520. doi: 10.3389/fncel.2024.1354520 (PMC11153811; doi:10.3389/fncel.2024.1354520)
Supplement: Supplementary file 9 [file Data_Sheet_2.pdf]

Supplementary Results

Search for molecular signatures in each cluster

We analyzed the DEGs between pLSOs and LOCs (Figure 4 A, Supplementary Table S3) and identified top DEGs by their AUROC values. In Figure 4 B, the expression of the top 40 DEGs, 20 in each cluster, is visualized in heatmaps (56 neurons in cluster 1, 30 in cluster 2). We provide background information for DEGs#1 -6 of both clusters in the main text file and in the following for DEGs#7 – 20.

Cluster 1

#7 – *Rorb*: RAR-related orphan receptor beta

Rorb (DEG#7) encodes the transcription factor RAR-related orphan receptor beta, aka RORβ (RAR = retinoic acid related). It is a layer-specific marker gene for neocortical layer four (Fernández de Sevilla et al., 2021; Yao et al., 2021) where its expression product is detected in a subtype of glutamatergic excitatory neurons (Hu et al., 2017). RORβ is joined by the isoforms RORα and RORγ. In the fetal mouse cochlea, *Rorb* expression exhibits an intriguing apical-to-basal gradient (Li et al., 2020), implying that it may be involved in the establishment of cochleotopy (= tonotopy). In the central auditory system, *Rorb* expression has been reported in the dorsal cochlear nucleus, the inferior colliculus, the medial geniculate body, and the auditory cortex (Liu et al., 2017). Interestingly, AMBA shows intermediate to high expression at P14 and low to intermediate expression levels for *Rorb* mRNA in the LSO at P56 (Supplementary Figure S4).

#8 – *Plk5*: polo-like kinase 5

Plk5 (DEG#8) represents one of five members in the mammalian *Plk* family. It encodes the polo-like kinase 5. *Plk5* is mainly expressed in differentiated tissues. Immunoreactivity is high in certain CNS regions, mainly in the cytoplasm of cortical neurons and cerebellar granular cells (de Carcer et al., 2011b). *Plk5* is a marker gene for type II spiral ganglion neurons (Petitpré et al., 2018; Shrestha et al., 2018). As to date, there are no published results on central auditory neurons. Although the PLK5 protein shows no catalytic kinase activity, in contrast to the other *Plk* members, it retains important functions (de Carcer et al., 2011a). In neurons, these functions comprise non-proliferative effects and involve modulation of neurite formation or maintenance of neurites. AMBA shows mainly intermediate expression levels at P56, with few cells showing a low signal intensity in the LSO (Supplementary Figure S4, no AMBA data available for P14).

#9 – *Lrfn5*: synaptic adhesion-like molecule 5

Lrfn5 (DEG#9) encodes leucine rich repeat and fibronectin type III domain containing 5, also known as synaptic adhesion-like molecule 5 (SALM5). The SALM family contains five homologous molecules, all of them adhesion molecules that are expressed largely in the CNS (Liu, 2019). The SALM5 protein displays homophilic and trans-cellular adhesion (Seabold et al., 2008). It is a postsynaptic organizer and thus regulates synaptic transmission, particularly via AMPA receptors (Choi et al., 2016). The authors mention that it also regulates inhibitory synaptic transmission. AMBA shows mostly low *Lrfn5* expression in the LSO at P56 (Supplementary Figure S4, no AMBA data available for P14).

#10 – *Ryr3*: ryanodine receptor type 3

Ryr3 (DEG#10) encodes ryanodine receptor type 3 (RyR3). RyRs are intracellular Ca^{2+} channels which mediate Ca^{2+}-induced Ca^{2+} release from internal stores. The RyR3 protein is abundant in specific brain regions, such as hippocampus, corpus striatum, and diencephalon (Murayama and Ogawa, 1996). In the hippocampus, it contributes to long-term potentiation,

dendritic spine formation, and spatial memory (Adasme et al., 2011). Interestingly, outer hair cells feature RyR1 in a pattern like cardiac myocytes do, prompting the idea that Ca^{2+} influx plays a similar role in regulating their contractability (Grant et al., 2006). It has been suggested that the Ca^{2+} effects mediated via RyR1 activate K^{+} channels, thereby inhibiting the hair cells (Lioudyno et al., 2004). To our knowledge, *Ryr3* expression in auditory brainstem neurons has not been described thus far. AMBA shows no expression in the LSO at P56 (Supplementary Figure S4, no AMBA data available for P14).

#11 – *Cplx1*: complexin 1

Cplx1 (DEG#11) codes for the protein complexin 1 (Cplx1). There are four paralogues (Cplx1-4) that cooperate with SNARE complexes at a post-priming step in synaptic vesicle fusion. Their loss causes profound deficits in the release probability (Brose, 2008; Chang et al., 2015). At endbulb of Held synapses between the auditory nerve fibers and CN neurons, it is required for high-fidelity transmission (Strenzke et al., 2009). At calyx of Held synapses, Cplx1 stabilizes newly primed vesicles and prevents their premature fusion. Cplx1-deficient calyces display excessive asynchronous release upon stimulation, thus triggering aberrant action potentials (Chang et al., 2015). Thus far, nothing was known for *Cplx1* expression in the LSO. AMBA shows high expression levels throughout the LSO at P56 (Supplementary Figure S4, no AMBA data available for P14).

#12 – *Cacng5*: transmembrane AMPAR regulatory protein Gamma-5

Cacng5 (DEG#12) encodes the regulatory γ5 subunit of Cav channels, which is also known as TARPγ5 (transmembrane AMPAR regulatory protein Gamma-5). TARPγ5 effects differ considerably from those of the canonical TARPs, as TARPγ5 augments glutamatergic currents only at AMPA receptors that contain the R-edited form of GluA2 subunits (Kato et al., 2008). It is therefore defined as a type II TARP. TARPγ5 increases the rate of GluA2 deactivation and desensitization and decreases glutamate potency. Interestingly, pLSOs of early postnatal mice have Ca^{2+}-impermeable AMPA receptors (Ene et al., 2003; Case et al., 2011) which thus contain most likely GluA2 subunits (Hollmann et al., 1991; Verdoorn et al., 1991). In accordance with the physiological results, immunohistochemical studies have detected GluA2 in the LSO of neonatal rats (Caicedo and Eybalin, 1999). AMBA shows intermediate to high expression at P14 and low to intermediate levels of *Cacng5* expression in the LSO at P56 (Supplementary Figure S4). Collectively, these results provide some evidence for the association of cluster 1 with pLSOs.

#13 – *Ralgapa2*: Ral GTPase activating protein catalytic subunit α2

Ralgapa2 (DEG#13) encodes for the catalytic subunit α2 of the Ral GTPase activating protein. Ral proteins are small GTPases. Their activity is downregulated by GTPase-activating protein for which we have only little information. RALGAPA2 is a negative regulator of glucose transporter type 4 translocation and thus involved in glucose uptake, the first step of glycolysis. There is no knowledge in the literature about Ralgapa2 in auditory neurons and the brain in general. AMBA shows low to intermediate gene expression in the LSO at P56 (Supplementary Figure S4, no AMBA data available for P14).

#14 – *Tshz2*: teashirt 2

Tshz2 (DEG#14) encodes for a zinc-finger homeobox transcription factor named teashirt 2 (TSHZ2). Such zinc-finger proteins can act as transcriptional repressors. Knowledge on *Tshz2* in conjunction with neurons is very sparse. It is differentially and highly expressed in spiral ganglion neurons of the subtype Ia (Petitpré et al., 2018) and a marker gene for layer five and six neocortical cells (Tasic et al., 2018). It was recently speculated that *Tshz* may be a master regulator establishing a medial/lateral gradient during cortical development (Yao et al., 2021).

As to date, nothing is known about TSHZ2 in the brainstem. This contrasts with the isoform *Tshz3*, whose gene product controls the development of the brainstem circuitry required for breathing. AMBA shows virtually no *in situ* signal in the LSO at P56 (Supplementary Figure S4, no AMBA data available for P14).

#15 – *Nebl*: nebulette

Nebl (DEG#15) has two major isoforms differing in their N-terminals. The longer isoform codes for the protein Nebulette, the longer one for the protein Lasp2 (LIM and SH3 protein 2; (Rodriguez et al., 2020). Nebulette appears to be exclusive to the heart, and the protein is functionally related to the larger homologous protein Nebulin, the second-largest protein in myofibers. It is associated with the Z-disks and thought to regulate the stabilization and function of actin filaments (Littlefield and Fowler, 2008). Nebulette acts in synergy with Nebulin to fine-tune muscle relaxation-contraction cycles (Hernandez et al., 2016). Contrary to our knowledge about Nebulette in muscle tissue, very little is known about its presence and function in the nervous system. A link of *Nebl* with Parkinson disease has been established (Licker et al., 2014). The *Lasp-2* gene is highly expressed in the chicken brain (Terasaki et al., 2004). Data from rat hippocampi demonstrate its involvement in synapse formation and the long-term maintenance of dendrites and dendritic spines (Myers et al., 2020). AMBA shows no *Nebl* expression in the LSO at P56 (Supplementary Figure S4, no AMBA data available for P14).

#16 – *Tcf4*: transcription factor 4

Tcf4 (DEG#16). Like *Tshz2* (#14 in this cluster), *Tcf4* codes for transcription factor 4 (TCF4). *Tcf4* is highly expressed in the developing forebrain (Jung et al., 2018). TCF4 protein is also present in the nuclei and cytoplasm of cochlear outer hair cells and considered to control the differentiation of these cells (Wang et al., 2021). It may regulate the formation of inhibitory synapses and the maintenance of excitatory/inhibitory balance (Tang et al., 2021). TCF4 regulates the expression of cell adhesion molecules and thus controls neuronal positioning (Zhang et al., 2021). In *Tcf4* knockout mice, cortical microcircuits are disrupted, and the neocortex resembles the 3-layered entorhinal cortex. Moreover, neuronal functions are disrupted. A *Tcf4*-dependent network of transcription factors has been identified, which cooperatively control the development of interhemispheric connectivity (Wittmann et al., 2021). Information about *Tcf4* on the central auditory system is lacking. AMBA shows low to intermediate expression at P14 and low gene expression in LSO cells at P56 (Supplementary Figure S4).

#17 – *Bcat1*: branched-chain aminotransferase

Bcat1 (DEG#17) is expressed in several organs, including a variety of brain regions, particularly the cerebellum, hippocampus, and ventral midbrain, where the expression is restricted to neurons (Sweatt et al., 2004; Castellano et al., 2007). The gene encodes the branched-chain aminotransferase 1 (BCAT1), a cytosolic enzyme involved in the catabolism of branched-chain amino acids. Therewith, it is involved in the synthesis of glutamate. Remarkably, the anticonvulsant drug gabapentin is a specific inhibitor of BCAT1. Literature data connecting *Bcat1* to auditory neurons are lacking. AMBA shows heterogeneous expression levels in the LSO ranging from low to high at both P14 and P56 (Supplementary Figure S4).

#18 – *Parp8*: poly(ADP-ribose) polymerase family member 8

Parp8 (DEG#18) codes for the eighth member of the poly(ADP-ribose) polymerase family. This family comprises 17 members. The proteins PARP1 and PARP2 have a well-established role in DNA repair. In contrast, the biological pathways PARP8 is involved in remain uncovered, and a cellular function has not been established (Richard et al., 2021). There is no literature data on *Parp8* expression in the auditory system. AMBA shows low to intermediate

signal intensities in the LSO at P56 (Supplementary Figure S4, no AMBA data available for P14).

#19 – *Cntn4*: Contactin-4

Cntn4 (DEG#19) encodes the protein Contactin-4 (aka BIG-2). BIG-2 is a GPI-anchored axonal glycoprotein and belongs to the superfamily of immunoglobulin cell adhesion molecules (Yoshihara et al., 1995; Oguro-Ando et al., 2017). BIG-2 promotes neurite outgrowth and target-specific axon arborization (Mercati et al., 2013; Osterhout et al., 2015). We know of no publications on *Cntn4* in the auditory system. AMBA shows low *in situ* hybridization signals in a few LSO cells at P56 (Supplementary Figure S4, no AMBA data available for P14).

#20 – *Cck*: cholecystokinin

Finally, *Cck* (DEG#20) codes for the neuropeptide cholecystokinin (CCK), which is widely distributed throughout the mammalian brain (Crawley and Corwin, 1994). CCK was described in rat LSOs projecting into the inferior colliculus which therewith are part of the ascending auditory system (Fallon and Seroogy, 1984). This result, together with the observation of medium-to-large soma sizes (13-30 µm), indicates that the CCK-positive neurons are pLSOs. AMBA shows low *Cck* expression only in a few cells in the LSO at P14 and no expression at P56 (Supplementary Figure S4).

Supplementary Figure S4 relevant here

Cluster 2

#7 – *Col4a4*: collagen type IV α4 chain

Col4a4 (DEG#7) encodes the α4 chain of type IV collagen. Notice that *Col4a3* was DEG#3 in cluster 2. The α3/α4/α5 domains have a limited tissue distribution. Mutations in *Col4a3* and *Col4a4* affect type IV collagens, essential components of the basement membranes in various organs. Patients with thin basement disease suffer from hematuria yet have no extrarenal manifestations, whereas those with Alport syndrome often present hearing loss and ocular abnormalities too (Plaisier et al., 2005; Cosgrove and Madison, 2022). AMBA shows intermediate *Col4a3* expression in the LSO at P56 (Supplementary Figure S5, no AMBA data available for P14). *Col4a4* was very recently identified as a marker for LOCs in a study using single-nucleus sequencing in mice, confirming our findings (Frank et al., 2023).

#8 – *Cit*: citron kinase

Cit (DEG#8) encodes citron kinase (CIT-K), a serine/threonine kinase that interacts with the small GTPase Rho in the Rho signaling pathway (D'Avino, 2017). *Cit* is expressed in various organs, including the CNS, and is involved in neuron generation. It also functions in DNA damage control. Mutations have been associated with microencephaly (Basit et al., 2016; Shaheen et al., 2016). AMBA shows low to intermediate *Cit* expression in the LSO at P56 and intermediate expression at P14 (Supplementary Figure S5).

#9 – *Fxyd6*: phosphohippolin

Fxyd6 (DEG#9) encodes a small single-span membrane protein FXYD6 also called phosphohippolin. FXYD6 binds and modulates the function of Na^+/K^+-ATPase (Garty and Karlish, 2006; Delprat et al., 2007b). Within the seven FXYD subfamilies, FXYD6 and FXYD7 are the major isoforms in the CNS (Delprat et al., 2007a). FXYD6 exhibits a unique distribution, with the highest levels in 3-week-old rat brains (Kadowaki et al., 2004). It was identified in glutamatergic synaptosomes from mouse brains (Biesemann et al., 2014) and in somatic and dendritic plasma membranes of spiral ganglion neurons (Delprat et al., 2007a), particularly type

II neurons (Shrestha et al., 2018), as well as in interdental cells of the cochlea (Kolla et al., 2020). The gene is dynamically expressed in the inner ear and may play a role in endolymph homeostasis and neuronal activity (Delprat et al., 2007a). *Fxyd6* expression is negatively correlated with the amplitude of AP afterhyperpolarization, indicating that phosphohippolin is involved in AP repolarization (Bomkamp et al., 2019). In this context, higher *Fxyd6* expression occurs in neurons that fire APs more slowly (Harris et al., 2018), consistent with the idea of cluster 2 comprising LOCs. AMBA shows LSO cells with low to intermediate *Fxyd6* expression levels at P56, but high expression levels at P14 (Supplementary Figure S5).

#10 – *Acly*: ATP citrate lyase

Acly (DEG#10) encodes an ATP citrate lyase. ACLY is a mitochondrial enzyme that catalyzes the conversion of citrate to cytosolic acetyl-CoA, thereby linking glucose metabolism to *de novo* lipid synthesis (Hatzivassiliou et al., 2005). It therewith provides a carbon source for the generation of fatty acids, cholesterol, and keton bodies. Results about neurons are sparse as to date. A recent report on single striatal neurons showed a negative correlation between *Acly* expression and the amplitude of IPSCs, indicating a role for ACLY at inhibitory synapses (Paraskevopoulou et al., 2021). The AMBA shows an intermediate signal intensity for *Acly* mRNA in the SOC at P56. (Supplementary Figure S5, no AMBA data available for P14).

#11 – *Scn3a*: sodium voltage-gated channel alpha subunit 3

Scn3a (DEG#11) codes for the pore-forming α subunit of voltage-activated Nav1.3 channels. AMBA shows no expression of *Scn3a* in the LSO at P54 (Supplementary Figure S5, no AMBA data available for P14). This gene is extensively discussed in the main text file.

#12 – *Gata3*: GATA binding protein 3

Gata3 (*DEG*#12) codes for the zinc-finger transcription factor GATA3. GATA3 immunopositivity has been described in the inner ear and in afferent and efferent auditory neurons of adult mice (Karis et al., 2001). The authors demonstrated GATA3 labeling in the LSO that matches with the pattern of retrogradely labeled LOCs (their Figure 3 g, h). These results suggest that *Gata3* is likely associated with LOCs. *Gata3* null mutants show unusual axonal projections, suggesting that the transcription factor is involved in axonal pathfinding of olivocochlear neurons (Karis et al., 2001). The authors also showed that continued *Gata3* expression is required for normal neurosensory development in the cochlea. AMBA shows only two LSO cells with low *Gata3* expression at P56 (Supplementary Figure S5). In contrast, the expression level at P14 is intermediate.

#13 – *Nfia*: nuclear factor I-A

Nfia (DEG#13) codes for the nuclear factor I-A (NFIA). *Nfia* is a master switch gene that turns on glia generation (Li, 2020). It also plays a role in astrocyte differentiation and continues to be expressed in mature astrocytes across various brain regions. In the hippocampus, it renders region-specific properties to astrocytes. Commissural projections develop abnormally in *Nfia* KO mice (Shu et al., 2003). *Nfia* expression was also described for neurons (Sagner et al., 2021). AMBA shows only one LSO cell with low expression signal at P56 but intermediate expression at P14 (Supplementary Figure S5).

#14 – *Rmst*: rhabdomyosarcoma 2 associated transcript

Rmst (DEG#14) encodes a long non-coding RNA molecule named rhabdomyosarcoma 2 associated transcript (RMST). Such RNAs play important roles in CNS development (Cheng et al., 2019). RMST can directly bind to SOX2 and is thus important for neuronal differentiation (Ng et al., 2013). In accord with this, *Rmst* expression increases during neuronal differentiation. A Pubmed search ‘rmst auditory’ (2024-03-07) yielded no result, and two results of marginal

interest were obtained with 'rmst brainstem'. AMBA provides no data for this gene (Supplementary Figure S5).

#15 – *Atp8a1*: ATPase 8A1

Atp8a1 (DEG#15) encodes the phospholipid transporting ATPase 8A1. The protein is a phospholipid flippase that transports a relatively giant substrate compared to Na^+/K^+-ATPases or Ca^{2+}-ATPases. It maintains a physiological distribution of phosphatidylserine, a glycerophospholipid and component of cell membranes (Bradberry et al., 2022). Via several key signaling pathways, phosphatidylserines indirectly stimulate neuronal survival, neurite outgrowth, and synaptogenesis. *Atp8a1* may also drive the formation of highly curved membranes of synaptic vesicles (Takada et al., 2018). AMBA shows LSO cells with low to intermediate *Atp8a1* expression at P56, but a high expression at P14 (Supplementary Figure S5).

#16 – *Resp18*: regulated endocrine-specific protein 18

Resp18 (DEG#16) encodes regulated endocrine-specific protein 18 (RESP18). Aside from other organs, Resp18 is expressed in the CNS, including the forebrain and brainstem (Atari et al., 2019). Gene expression has been attributed to peptidergic and brainstem catecholaminergic neurons (Darlington et al., 1997). The authors do not mention auditory nuclei. The RESP18 protein may serve as an intracellular signal, but the function is unclear. In the thyroid, RESP18 colocalizes with CGRP, DEG#1 in cluster 2. AMBA shows heterogenous expression levels for *Resp18* in the LSO at P56 (Supplementary Figure S5, no AMBA data available for P14).

#17 – *Spock3*: SPARC/Osteonectin CWCV and Kazal-like domains proteoglycan 3

Spock3 (DEG#17) encodes the protein SPARC, the acronym for secreted heparan sulfate proteoglycan acidic and rich in cysteine. SPARC belongs to the osteonectin family of extracellular Ca^{2+}-binding proteins. *Spock3* itself is the acronym for SPARC/Osteonectin CWCV and Kazal-like domains proteoglycan 3 and had previously been identified as testican-3 (Charbonnier et al., 1998). *Spock3* is broadly expressed in the developing and adult brain, with a bias towards fetal and early postnatal stages. Mutant mice display abnormalities of axonal tracts in the brain (Yamamoto et al., 2014). These results show an important role for *Spock3* in the formation or maintenance of major neuronal structures in the brain and a possible involvement of SPARC in axon guidance. AMBA shows mostly low to intermediate *Spock3* expression at P56 (Supplementary Figure S5, no AMBA data available for P14).

#18 – *Pgrmc1*: progesterone receptor membrane component 1

Pgrmc1 (DEG#18) encodes PGRMC-1, a putative membrane-associated steroid receptor named progesterone receptor membrane component 1. A Pubmed search ('pgrmc brain'; 2023-10-22) revealed 3 results, from which a function or localization of the protein cannot be deduced. AMBA shows heterogenous expression levels in the LSO at P56 (Supplementary Figure S5, no AMBA data available for P14).

#19 – *Rasgrf1*: Ras protein specific guanine nucleotide releasing factor 1

Rasgrf1 (DEG#19) codes for a protein named Ras protein specific guanine nucleotide releasing factor 1 (RasGRF1). RafGRIF1 is a Ca^{2+}-stimulated guanine nucleotide exchange factor (GEF) that stimulates the dissociation of GDP from the Ras protein. RasGRF1 contains a neural domain that allows specific binding to the cytoplasmic C-terminal of GluN2B-subunits in NMDA receptors (Sepulveda et al., 2010; Varbanov and Dityatev, 2017). The GluN2B-NMDAR/RasGRF1/NOX2 pathway promotes dendritogenesis, whereas disruption of the interaction between GluN2B and RasGRF1 decreases dendritic branching (Krapivinsky et al., 2003; Abarzua et al., 2019). Ras-GEF activity can be activated by muscarinic receptors and

Ca^{2+} influx, and it may be important for long-term memory. AMBA shows heterogenous expression levels for *Rasgrf* throughout the LSO at P56 and intermediate expression at P14 (Supplementary Figure S5).

#20 – *Gm20721*: snRNA-activating protein complex subunit 3

Finally, *Gm20721* (DEG#20) encodes the subunit 3 of a snRNA-activating protein complex (SNAPC3). The SNAPC complex is composed of five subunits and regulates the transcription of genes coding for non-coding, small nuclear RNAs (Baillat et al., 2012). The subunits are conserved among multicellular organisms. The SNAP3 subunit can directly bind DNA (Jawdekar et al., 2006) and, together with SNAPC1 and SNAPC4, can form a ‘minimal’ functional SNAPC (Mittal et al., 1999). Several Pubmed searches (e.g., ‘SNAPC3 brain’; ‘SNAPC3 neuron’; 2024-03-07) obtained only one result of marginal interest, and AMBA provides no data for *Gm20721* in the LSO (Supplementary Figure S5). Thus, *Gm2071* is the DEG for which we obtained the least information in the context of neural tissue.

Supplementary Figure S5 relevant here

References

Abarzua, S., Ampuero, E., and Van Zundert, B. (2019). Superoxide generation via the NR2B-NMDAR/RasGRF1/NOX2 pathway promotes dendritogenesis. *J. Cell Physiol.* 234**,** 22985-22995. doi: 10.1002/jcp.28859

Adasme, T., Haeger, P., Paula-Lima, A. C., Espinoza, I., Casas-Alarcón, M. M., Carrasco, M. A., and Hidalgo, C. (2011). Involvement of ryanodine receptors in neurotrophin-induced hippocampal synaptic plasticity and spatial memory formation. *Proc. Natl. Acad. Sci. U.S.A.* 108**,** 3029-3034. doi: 10.1073/pnas.1013580108

Andrews, T. S., and Hemberg, M. (2018). False signals induced by single-cell imputation. *F1000Res* 7**,** 1740. doi: 10.12688/f1000research.16613.2

Atari, E., Perry, M. C., Jose, P. A., and Kumarasamy, S. (2019). Regulated endocrine-specific protein-18, an emerging endocrine protein in physiology: A literature review. *Endocrinology* 160**,** 2093-2100. doi: 10.1210/en.2019-00397

Baillat, D., Gardini, A., Cesaroni, M., and Shiekhattar, R. (2012). Requirement for SNAPC1 in transcriptional responsiveness to diverse extracellular signals. *Mol. Cell Biol.* 32**,** 4642-4650. doi: 10.1128/MCB.00906-12

Basit, S., Al-Harbi, K. M., Alhijji, S. A., Albalawi, A. M., Alharby, E., Eldardear, A., and Samman, M. I. (2016). CIT, a gene involved in neurogenic cytokinesis, is mutated in human primary microcephaly. *Hum. Genet.* 135**,** 1199-1207. doi: 10.1007/s00439-016-1724-0

Biesemann, C., Gronborg, M., Luquet, E., Wichert, S. P., Bernard, V., Bungers, S. R., Cooper, B., Varoqueaux, F., Li, L., Byrne, J. A., Urlaub, H., et al. (2014). Proteomic screening of glutamatergic mouse brain synaptosomes isolated by fluorescence activated sorting. *EMBO J.* 33**,** 157-170. doi: 10.1002/embj.201386120

Bomkamp, C., Tripathy, S. J., Bengtsson Gonzales, C., Hjerling-Leffler, J., Craig, A. M., and Pavlidis, P. (2019). Transcriptomic correlates of electrophysiological and morphological diversity within and across excitatory and inhibitory neuron classes. *PLoS Comput. Biol.* 15**,** e1007113. doi: 10.1371/journal.pcbi.1007113

Bradberry, M. M., Mishra, S., Zhang, Z., Wu, L., Mcketney, J. M., Vestling, M. M., Coon, J. J., and Chapman, E. R. (2022). Rapid and gentle immunopurification of brain synaptic vesicles. *J. Neurosci.* doi: 10.1523/jneurosci.2521-21.2022

Brose, N. (2008). For better or for worse: complexins regulate SNARE function and vesicle fusion. *Traffic* 9**,** 1403-1413. doi: 10.1111/j.1600-0854.2008.00758.x

Caicedo, A., and Eybalin, M. (1999). Glutamate receptor phenotypes in the auditory brainstem and mid-brain of the developing rat. *Eur. J. Neurosci.* 11**,** 51-74. doi:

Case, D. T., Zhao, X., and Gillespie, D. C. (2011). Functional refinement in the projection from ventral cochlear nucleus to lateral superior olive precedes hearing onset in rat. *PLoS One* 6**,** e20756. doi: 10.1371/journal.pone.0020756

Castellano, S., Casarosa, S., Sweatt, A. J., Hutson, S. M., and Bozzi, Y. (2007). Expression of cytosolic branched chain aminotransferase (BCATc) mRNA in the developing mouse brain. *Gene Expr Patterns* 7**,** 485-490. doi: 10.1016/j.modgep.2006.10.010

Chang, S., Reim, K., Pedersen, M., Neher, E., Brose, N., and Taschenberger, H. (2015). Complexin stabilizes newly primed synaptic vesicles and prevents their premature fusion at the mouse calyx of Held synapse. *J. Neurosci.* 35**,** 8272-8290. doi: 10.1523/jneurosci.4841-14.2015

Charbonnier, F., Perin, J. P., Mattei, M. G., Camuzat, A., Bonnet, F., Gressin, L., and Alliel, P. M. (1998). Genomic organization of the human SPOCK gene and its chromosomal localization to 5q31. *Genomics* 48**,** 377-380. doi: 10.1006/geno.1997.5199

Cheng, X., Li, H., Zhao, H., Li, W., Qin, J., and Jin, G. (2019). Function and mechanism of long non-coding RNA Gm21284 in the development of hippocampal cholinergic neurons. *Cell Biosci.* 9**,** 72. doi: 10.1186/s13578-019-0336-5

Choi, Y., Nam, J., Whitcomb, D. J., Song, Y. S., Kim, D., Jeon, S., Um, J. W., Lee, S. G., Woo, J., Kwon, S. K., Li, Y., et al. (2016). SALM5 trans-synaptically interacts with LAR-RPTPs in a splicing-dependent manner to regulate synapse development. *Sci. Rep.* 6**,** 26676. doi: 10.1038/srep26676

Cosgrove, D., and Madison, J. (2022). Molecular and cellular mechanisms underlying the initiation and progression of Alport glomerular pathology. *Front. Med.* 9**,** 846152. doi: 10.3389/fmed.2022.846152

Crawley, J. N., and Corwin, R. L. (1994). Biological actions of cholecystokinin. *Peptides* 15**,** 731-755. doi: 10.1016/0196-9781(94)90104-x

D'avino, P. P. (2017). Citron kinase - renaissance of a neglected mitotic kinase. *J. Cell Sci.* 130**,** 1701-1708. doi: 10.1242/jcs.200253

Darlington, D. N., Schiller, M. R., Mains, R. E., and Eipper, B. A. (1997). Expression of RESP18 in peptidergic and catecholaminergic neurons. *J. Histochem. Cytochem.* 45**,** 1265-1277. doi: 10.1177/002215549704500910

De Carcer, G., Escobar, B., Higuero, A. M., Garcia, L., Anson, A., Perez, G., Mollejo, M., Manning, G., Melendez, B., Abad-Rodriguez, J., and Malumbres, M. (2011a). Plk5, a polo box domain-only protein with specific roles in neuron differentiation and glioblastoma suppression. *Mol. Cell Biol.* 31**,** 1225-1239. doi: 10.1128/MCB.00607-10

De Carcer, G., Manning, G., and Malumbres, M. (2011b). From Plk1 to Plk5: functional evolution of polo-like kinases. *Cell Cycle* 10**,** 2255-2262. doi: 10.4161/cc.10.14.16494

Delprat, B., Puel, J. L., and Geering, K. (2007a). Dynamic expression of FXYD6 in the inner ear suggests a role of the protein in endolymph homeostasis and neuronal activity. *Dev. Dyn.* 236**,** 2534-2540. doi: 10.1002/dvdy.21269

Delprat, B., Schaer, D., Roy, S., Wang, J., Puel, J. L., and Geering, K. (2007b). FXYD6 is a novel regulator of Na,K-ATPase expressed in the inner ear. *J. Biol. Chem.* 282**,** 7450-7456. doi: 10.1074/jbc.M609872200

Ene, F. A., Kullmann, P. H. M., Gillespie, D. C., and Kandler, K. (2003). Glutamatergic calcium responses in the developing lateral superior olive: receptor types and their specific activation by synaptic activity patterns. *J. Neurophysiol.* 90**,** 2581-2591. doi:

Fallon, J. H., and Seroogy, K. B. (1984). Visual and auditory pathways contain cholecystokinin: evidence from immunofluorescence and retrograde tracing. *Neurosci. Lett.* 45**,** 81-87. doi: 10.1016/0304-3940(84)90333-1

Fernández De Sevilla, D., Nuñez, A., Araque, A., and Buño, W. (2021). Metabotropic regulation of synaptic plasticity. *Neuroscience* 456**,** 1-3. doi: 10.1016/j.neuroscience.2020.10.006

Frank, M. M., Sitko, A. A., Suthakar, K., Cadenas, L. T., Hunt, M., Yuk, M. C., Weisz, C. J. C., and Goodrich, L. V. (2023). Experience-dependent flexibility in a molecularly diverse central-to-peripheral auditory feedback system. *Elife* 12:e83855**,** e83855. doi: 10.7554/eLife.83855

Garty, H., and Karlish, S. J. (2006). Role of FXYD proteins in ion transport. *Annu. Rev. Physiol.* 68**,** 431-459. doi: 10.1146/annurev.physiol.68.040104.131852

Grant, L., Slapnick, S., Kennedy, H., and Hackney, C. (2006). Ryanodine receptor localisation in the mammalian cochlea: an ultrastructural study. *Hear. Res.* 219**,** 101-109. doi: 10.1016/j.heares.2006.06.002

Harris, K. D., Hochgerner, H., Skene, N. G., Magno, L., Katona, L., Bengtsson Gonzales, C., Somogyi, P., Kessaris, N., Linnarsson, S., and Hjerling-Leffler, J. (2018). Classes and continua of hippocampal CA1 inhibitory neurons revealed by single-cell transcriptomics. *PLoS Biol* 16**,** e2006387. doi: 10.1371/journal.pbio.2006387

Hatzivassiliou, G., Zhao, F., Bauer, D. E., Andreadis, C., Shaw, A. N., Dhanak, D., Hingorani, S. R., Tuveson, D. A., and Thompson, C. B. (2005). ATP citrate lyase inhibition can suppress tumor cell growth. *Cancer Cell* 8**,** 311-321. doi: 10.1016/j.ccr.2005.09.008

Hernandez, D. A., Bennett, C. M., Dunina-Barkovskaya, L., Wedig, T., Capetanaki, Y., Herrmann, H., and Conover, G. M. (2016). Nebulette is a powerful cytolinker organizing desmin and actin in mouse hearts. *Mol. Biol. Cell* 27**,** 3869-3882. doi: 10.1091/mbc.E16-04-0237

Hollmann, M., Hartley, M., and Heinemann, S. (1991). Ca^{2+} permeability of KA-AMPA-gated glutamate receptor channels depends on subunit composition. *Science* 252**,** 851-853. doi: 10.1126/science.1709304

Hu, P., Fabyanic, E., Kwon, D. Y., Tang, S., Zhou, Z., and Wu, H. (2017). Dissecting cell-type composition and activity-dependent transcriptional state in mammalian brains by massively parallel single-nucleus RNA-Seq. *Mol. Cell* 68**,** 1006-1015 e1007. doi: 10.1016/j.molcel.2017.11.017

Jawdekar, G. W., Hanzlowsky, A., Hovde, S. L., Jelencic, B., Feig, M., Geiger, J. H., and Henry, R. W. (2006). The unorthodox SNAP50 zinc finger domain contributes to cooperative promoter recognition by human SNAPC. *J. Biol. Chem.* 281**,** 31050-31060. doi: 10.1074/jbc.M603810200

Jung, M., Haberle, B. M., Tschaikowsky, T., Wittmann, M. T., Balta, E. A., Stadler, V. C., Zweier, C., Dorfler, A., Gloeckner, C. J., and Lie, D. C. (2018). Analysis of the expression pattern of the schizophrenia-risk and intellectual disability gene TCF4 in the developing and adult brain suggests a role in development and plasticity of cortical and hippocampal neurons. *Mol. Autism* 9**,** 20. doi: 10.1186/s13229-018-0200-1

Kadowaki, K., Sugimoto, K., Yamaguchi, F., Song, T., Watanabe, Y., Singh, K., and Tokuda, M. (2004). Phosphohippolin expression in the rat central nervous system. *Brain Res. Dev. Brain Res.* 125**,** 105-112. doi: 10.1016/j.molbrainres.2004.03.021

Karis, A., Pata, I., Van Doorninck, J. H., Grosveld, F., De Zeeuw, C. I., De Caprona, D., and Fritzsch, B. (2001). Transcription factor GATA-3 alters pathway selection of olivocochlear neurons and affects morphogenesis of the ear. *J. Comp. Neurol.* 429**,** 615-630. doi: 10.1002/1096-9861(20010122)429:4<615::aid-cne8>3.0.co;2-f

Kato, A. S., Siuda, E. R., Nisenbaum, E. S., and Bredt, D. S. (2008). AMPA receptor subunit-specific regulation by a distinct family of type II TARPs. *Neuron* 59**,** 986-996. doi: 10.1016/j.neuron.2008.07.034

Kolla, L., Kelly, M. C., Mann, Z. F., Anaya-Rocha, A., Ellis, K., Lemons, A., Palermo, A. T., So, K. S., Mays, J. C., Orvis, J., Burns, J. C., et al. (2020). Characterization of the development of the mouse cochlear epithelium at the single cell level. *Nat. Commun.* 11**,** 2389. doi: 10.1038/s41467-020-16113-y

Krapivinsky, G., Krapivinsky, L., Manasian, Y., Ivanov, A., Tyzio, R., Pellegrino, C., Ben-Ari, Y., Clapham, D. E., and Medina, I. (2003). The NMDA receptor is coupled to the ERK pathway by a direct interaction between NR2B and RasGRF1. *Neuron* 40**,** 775-784. doi: 10.1016/s0896-6273(03)00645-7

Li, C., Wang, Y., Wang, G., Lu, Y., He, S., Sun, Y., and Liu, Z. (2020). Fate-mapping analysis using Rorb-IRES-Cre reveals apical-to-basal gradient of Rorb expression in mouse cochlea. *Dev. Dyn.* 249**,** 173-186. doi: 10.1002/dvdy.111

Li, Q. (2020). Overseeing memory circuits by NFIA: new face in astrocytes. *Neuron* 106**,** 878-878. doi: 10.1016/j.neuron.2020.05.027

Licker, V., Turck, N., Kövari, E., Burkhardt, K., Côte, M., Surini-Demiri, M., Lobrinus, J. A., Sanchez, J. C., and Burkhard, P. R. (2014). Proteomic analysis of human substantia nigra identifies novel candidates involved in Parkinson's disease pathogenesis. *Proteomics* 14**,** 784-794. doi: 10.1002/pmic.201300342

Lioudyno, M., Hiel, H., Kong, J. H., Katz, E., Waldman, E., Parameshwaran-Iyer, S., Glowatzki, E., and Fuchs, P. A. (2004). A "synaptoplasmic cistern" mediates rapid inhibition of cochlear hair cells. *J. Neurosci.* 24**,** 11160-11164. doi: 10.1523/jneurosci.3674-04.2004

Littlefield, R. S., and Fowler, V. M. (2008). Thin filament length regulation in striated muscle sarcomeres: pointed-end dynamics go beyond a nebulin ruler. *Semin. Cell. Dev. Biol.* 19, 511-519. doi: 10.1016/j.semcdb.2008.08.009

Liu, H. (2019). Synaptic organizers: synaptic adhesion-like molecules (SALMs). *Curr. Opin. Struct. Biol.* 54, 59-67. doi: 10.1016/j.sbi.2019.01.002

Liu, H., Aramaki, M., Fu, Y., and Forrest, D. (2017). Retinoid-related orphan receptor beta and transcriptional control of neuronal differentiation. *Curr. Top. Dev. Biol.* 125, 227-255. doi: 10.1016/bs.ctdb.2016.11.009

Macosko, E. Z., Basu, A., Satija, R., Nemesh, J., Shekhar, K., Goldman, M., Tirosh, I., Bialas, A. R., Kamitaki, N., Martersteck, E. M., Trombetta, J. J., et al. (2015). Highly parallel genome-wide expression profiling of individual cells using nanoliter droplets. *Cell* 161, 1202-1214. doi: 10.1016/j.cell.2015.05.002

Mercati, O., Danckaert, A., Andre-Leroux, G., Bellinzoni, M., Gouder, L., Watanabe, K., Shimoda, Y., Grailhe, R., De Chaumont, F., Bourgeron, T., and Cloez-Tayarani, I. (2013). Contactin 4, -5 and -6 differentially regulate neuritogenesis while they display identical PTPRG binding sites. *Biol. Open* 2, 324-334. doi: 10.1242/bio.20133343

Mittal, V., Ma, B., and Hernandez, N. (1999). SNAP(c): a core promoter factor with a built-in DNA-binding damper that is deactivated by the Oct-1 POU domain. *Genes Dev.* 13, 1807-1821. doi: 10.1101/gad.13.14.1807

Murayama, T., and Ogawa, Y. (1996). Properties of Ryr3 ryanodine receptor isoform in mammalian brain. *J. Biol. Chem.* 271, 5079-5084. doi: 10.1074/jbc.271.9.5079

Myers, K. R., Yu, K., Kremerskothen, J., Butt, E., and Zheng, J. Q. (2020). The nebulin family LIM and SH3 proteins regulate postsynaptic development and function. *J. Neurosci.* 40, 526-541. doi: 10.1523/jneurosci.0334-19.2019

Ng, S. Y., Bogu, G. K., Soh, B. S., and Stanton, L. W. (2013). The long noncoding RNA RMST interacts with SOX2 to regulate neurogenesis. *Mol. Cell* 51, 349-359. doi: 10.1016/j.molcel.2013.07.017

Oguro-Ando, A., Zuko, A., Kleijer, K. T. E., and Burbach, J. P. H. (2017). A current view on contactin-4, -5, and -6: Implications in neurodevelopmental disorders. *Mol. Cell Neurosci.* 81, 72-83. doi: 10.1016/j.mcn.2016.12.004

Osterhout, J. A., Stafford, B. K., Nguyen, P. L., Yoshihara, Y., and Huberman, A. D. (2015). Contactin-4 mediates axon-target specificity and functional development of the accessory optic system. *Neuron* 86, 985-999. doi: 10.1016/j.neuron.2015.04.005

Paraskevopoulou, F., Parvizi, P., Senger, G., Tuncbag, N., Rosenmund, C., and Yildirim, F. (2021). Impaired inhibitory GABAergic synaptic transmission and transcription studied in single neurons by Patch-seq in Huntington's disease. *Proc. Natl. Acad. Sci. U.S.A.* 118. doi: 10.1073/pnas.2020293118

Petitpré, C., Wu, H., Sharma, A., Tokarska, A., Fontanet, P., Wang, Y., Helmbacher, F., Yackle, K., Silberberg, G., Hadjab, S., and Lallemend, F. (2018). Neuronal heterogeneity and stereotyped connectivity in the auditory afferent system. *Nat. Commun.* 9, 3691. doi: 10.1038/s41467-018-06033-3

Plaisier, E., Alamowitch, S., Gribouval, O., Mougenot, B., Gaudric, A., Antignac, C., Roullet, E., and Ronco, P. (2005). Autosomal-dominant familial hematuria with retinal arteriolar tortuosity and contractures: a novel syndrome. *Kidney Int.* 67, 2354-2360. doi: 10.1111/j.1523-1755.2005.00341.x

Richard, I. A., Burgess, J. T., O'byrne, K. J., and Bolderson, E. (2021). Beyond PARP1: The potential of other members of the poly (ADP-ribose) polymerase family in DNA repair and cancer therapeutics. *Front. Cell Dev. Biol.* 9, 801200. doi: 10.3389/fcell.2021.801200

Rodriguez, J. M., Pozo, F., Di Domenico, T., Vazquez, J., and Tress, M. L. (2020). An analysis of tissue-specific alternative splicing at the protein level. *PLoS Comput. Biol.* 16, e1008287. doi: 10.1371/journal.pcbi.1008287

Rosenberg, A. B., Roco, C. M., Muscat, R. A., Kuchina, A., Yao, Z., Graybuck, L. T., Peeler, D. J., Mukherjee, S., Chen, W., Pun, S. H., Sellers, D. L., et al. (2018). Single-cell profiling of the developing mouse brain and spinal cord with split-pool barcoding. *Science* 360**,** 176-182. doi: 10.1126/science.aam8999

Sagner, A., Zhang, I., Watson, T., Lazaro, J., Melchionda, M., and Briscoe, J. (2021). A shared transcriptional code orchestrates temporal patterning of the central nervous system. *PLoS Biol* 19**,** e3001450. doi: 10.1371/journal.pbio.3001450

Seabold, G. K., Wang, P. Y., Chang, K., Wang, C. Y., Wang, Y. X., Petralia, R. S., and Wenthold, R. J. (2008). The SALM family of adhesion-like molecules forms heteromeric and homomeric complexes. *J. Biol. Chem.* 283**,** 8395-8405. doi: 10.1074/jbc.M709456200

Sepulveda, F. J., Bustos, F. J., Inostroza, E., Zuniga, F. A., Neve, R. L., Montecino, M., and Van Zundert, B. (2010). Differential roles of NMDA Receptor Subtypes NR2A and NR2B in dendritic branch development and requirement of RasGRF1. *J. Neurophysiol.* 103**,** 1758-1770. doi: 10.1152/jn.00823.2009

Shaheen, R., Hashem, A., Abdel-Salam, G. M., Al-Fadhli, F., Ewida, N., and Alkuraya, F. S. (2016). Mutations in CIT, encoding citron rho-interacting serine/threonine kinase, cause severe primary microcephaly in humans. *Hum. Genet.* 135**,** 1191-1197. doi: 10.1007/s00439-016-1722-2

Shrestha, B. R., Chia, C., Wu, L., Kujawa, S. G., Liberman, M. C., and Goodrich, L. V. (2018). Sensory neuron diversity in the inner ear is shaped by activity. *Cell* 174**,** 1229-1246 e1217. doi: 10.1016/j.cell.2018.07.007

Shu, T., Butz, K. G., Plachez, C., Gronostajski, R. M., and Richards, L. J. (2003). Abnormal development of forebrain midline glia and commissural projections in Nfia knock-out mice. *J. Neurosci.* 23**,** 203-212. doi: 10.1523/jneurosci.23-01-00203.2003

Strenzke, N., Chanda, S., Kopp-Scheinpflug, C., Khimich, D., Reim, K., Bulankina, A. V., Neef, A., Wolf, F., Brose, N., Xu-Friedman, M. A., and Moser, T. (2009). Complexin-I is required for high-fidelity transmission at the endbulb of Held auditory synapse. *J. Neurosci.* 29**,** 7991-8004. doi:

Sweatt, A. J., Garcia-Espinosa, M. A., Wallin, R., and Hutson, S. M. (2004). Branched-chain amino acids and neurotransmitter metabolism: expression of cytosolic branched-chain aminotransferase (BCATc) in the cerebellum and hippocampus. *J. Comp. Neurol.* 477**,** 360-370. doi: 10.1002/cne.20200

Takada, N., Naito, T., Inoue, T., Nakayama, K., Takatsu, H., and Shin, H. W. (2018). Phospholipid-flipping activity of P4-ATPase drives membrane curvature. *EMBO J.* 37. doi: 10.15252/embj.201797705

Tang, X., Jaenisch, R., and Sur, M. (2021). The role of GABAergic signalling in neurodevelopmental disorders. *Nat. Rev. Neurosci.* doi: 10.1038/s41583-021-00443-x

Tasic, B., Yao, Z., Graybuck, L. T., Smith, K. A., Nguyen, T. N., Bertagnolli, D., Goldy, J., Garren, E., Economo, M. N., Viswanathan, S., Penn, O., et al. (2018). Shared and distinct transcriptomic cell types across neocortical areas. *Nature* 563**,** 72-78. doi: 10.1038/s41586-018-0654-5

Terasaki, A. G., Suzuki, H., Nishioka, T., Matsuzawa, E., Katsuki, M., Nakagawa, H., Miyamoto, S., and Ohashi, K. (2004). A novel LIM and SH3 protein (lasp-2) highly expressing in chicken brain. *Biochem Biophys Res Commun* 313**,** 48-54. doi: 10.1016/j.bbrc.2003.11.085

Van Den Hurk, M., Erwin, J. A., Yeo, G. W., Gage, F. H., and Bardy, C. (2018). Patch-seq protocol to analyze the electrophysiology, morphology and transcriptome of whole single neurons derived from human pluripotent stem cells. *Front. Mol. Neurosci.* 11**,** 261. doi: 10.3389/fnmol.2018.00261

Varbanov, H., and Dityatev, A. (2017). Regulation of extrasynaptic signaling by polysialylated NCAM: Impact for synaptic plasticity and cognitive functions. *Mol. Cell Neurosci.* 81**,** 12-21. doi: 10.1016/j.mcn.2016.11.005

Verdoorn, T. A., Burnashev, N., Monyer, H., Seeburg, P. H., and Sakmann, B. (1991). Structural determinants of ion flow through recombinant glutamate receptor channels. *Science* 252**,** 1715-1718. doi: 10.1126/science.1710829

Wang, S., Lee, M. P., Jones, S., Liu, J., and Waldhaus, J. (2021). Mapping the regulatory landscape of auditory hair cells from single-cell multi-omics data. *Genome Res.* 31**,** 1885-1899. doi: 10.1101/gr.271080.120

Wittmann, M. T., Katada, S., Sock, E., Kirchner, P., Ekici, A. B., Wegner, M., Nakashima, K., Lie, D. C., and Reis, A. (2021). scRNA sequencing uncovers a TCF4-dependent transcription factor network regulating commissure development in mouse. *Development* 148. doi: 10.1242/dev.196022

Wu, Y., and Zhang, K. (2020). Tools for the analysis of high-dimensional single-cell RNA sequencing data. *Nat. Rev. Nephrol.* 16**,** 408-421. doi: 10.1038/s41581-020-0262-0

Yamamoto, A., Uchiyama, K., Nara, T., Nishimura, N., Hayasaka, M., Hanaoka, K., and Yamamoto, T. (2014). Structural abnormalities of corpus callosum and cortical axonal tracts accompanied by decreased anxiety-like behavior and lowered sociability in spock3- mutant mice. *Dev. Neurosci.* 36**,** 381-395. doi: 10.1159/000363101

Yao, Z., Van Velthoven, C. T. J., Nguyen, T. N., Goldy, J., Sedeno-Cortes, A. E., Baftizadeh, F., Bertagnolli, D., Casper, T., Chiang, M., Crichton, K., Ding, S. L., et al. (2021). A taxonomy of transcriptomic cell types across the isocortex and hippocampal formation. *Cell*. doi: 10.1016/j.cell.2021.04.021

Yoshihara, Y., Kawasaki, M., Tamada, A., Nagata, S., Kagamiyama, H., and Mori, K. (1995). Overlapping and differential expression of BIG-2, BIG-1, TAG-1, and F3: four members of an axon-associated cell adhesion molecule subgroup of the immunoglobulin superfamily. *J. Neurobiol.* 28**,** 51-69. doi: 10.1002/neu.480280106

Zhang, L., and Zhang, S. (2021). Imputing single-cell RNA-seq data by considering cell heterogeneity and prior expression of dropouts. *J. Mol. Cell Biol.* 13**,** 29-40. doi: 10.1093/jmcb/mjaa052

Zhang, Y., Cai, Z., Hu, G., Hu, S., Wang, Y., Li, N., Chen, S., Liu, Q., Zeng, L., Tang, T., Zhang, Y., et al. (2021). Transcription factor 4 controls positioning of cortical projection neurons through regulation of cell adhesion. *Mol. Psychiatry* 26**,** 6562-6577. doi: 10.1038/s41380-021-01119-9

Zheng, G. X., Terry, J. M., Belgrader, P., Ryvkin, P., Bent, Z. W., Wilson, R., Ziraldo, S. B., Wheeler, T. D., Mcdermott, G. P., Zhu, J., Gregory, M. T., et al. (2017). Massively parallel digital transcriptional profiling of single cells. *Nat. Commun.* 8**,** 14049. doi: 10.1038/ncomms14049
